# Optimal and Secure Operation of a μ‑CHP Solid Oxide Fuel Cell System

**DOI:** 10.1021/acsomega.6c08789

**Published:** 2026-10-20

**Authors:** Hangyu Yu, Florian Bernard Berset, Tafarel de Avila Ferreira, Arne Sommerfeld, Gregor Holstermann, Cédric Frantz, Philippe Aubin, Guillaume Jeanmonod, Ligang Wang, Matthias Boltze, Grégory François, Jan Van herle

**Affiliations:** † Group of Energy Materials, 729280 Swiss Federal Institute of Technology in Lausanne (EPFL), Rue de l’Industrie 17, 1951 Sion, Valais, Switzerland; ‡ 111844 HES-SO Valais-Wallis, 1951 Sion, Valais, Switzerland; § New Enerday GmbH, Lindenstraße 45, 17033 Neubrandenburg, Germany; ∥ Hydro-Québec (IREQ), J3X1S1, Montreal, QC, Canada; ⊥ National Energy Storage Industry-Education Platform, 47840 North China Electric Power University, 102206 Beijing, China; # Beijing Laboratory of New Energy Storage Technology, North China Electric Power University, 102206 Beijing, China

## Abstract

Performance optimization and operational safety of solid oxide fuel cell (SOFC) systems are crucial for long-term operation and lifespan prolongation. However, optimal and secure operation under real-world conditions remains challenging. This study demonstrates a fast constraint-adaptation real-time optimization methodology that optimizes the electrical and combined-heat-and-power (CHP) efficiencies of a μ-CHP SOFC system across power targets, using transient measurements and a simplified dynamic model despite significant plant-model mismatch. The algorithm converged to power targets within four iterations (15 min), achieving at least 5% relative improvement in electrical efficiency through multi-input manipulation. Optimizing CHP efficiency increased hot water production by 20%. The algorithm remained robust against natural gas grid fluctuations and programmed control-subsystem interruptions while maintaining secure operation without safety shutdowns, demonstrating its maturity for optimal and safe operation of real-world SOFC systems.

## Graphical abstract

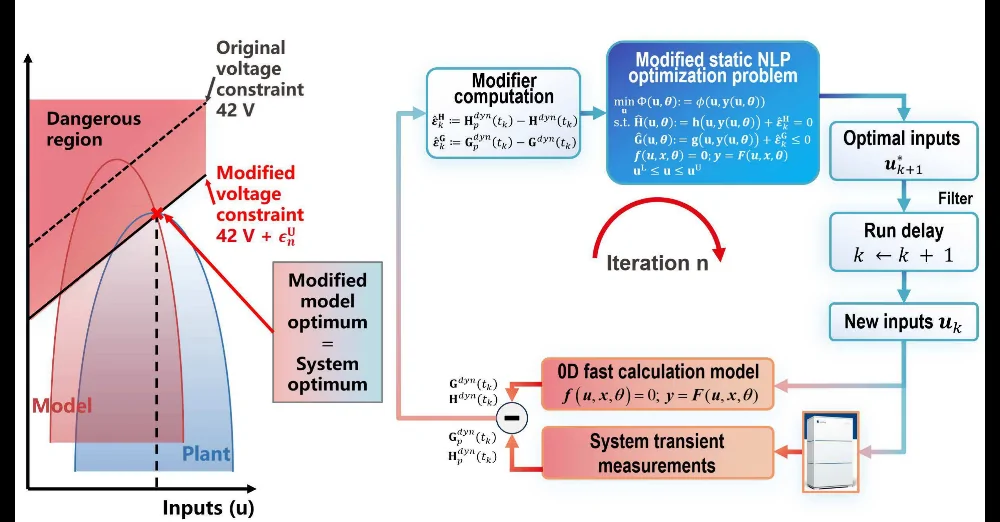

## Introduction

Solid oxide fuel cell (SOFC) technology emerges as a promising candidate for addressing global energy transition challenges and mitigating inefficiencies in nonrenewable energy utilization, demonstrating distinct advantages over alternative electrochemical energy systems.^1–3^ Modern engineering advances in compact balance-of-plant (BoP) design enable residential deployment of natural gas grid-connected microcombined-heat-and-power (μ-CHP) SOFC systems, which fully utilize natural gas infrastructure for simultaneous electricity generation and domestic hot water provision.^4,5^

Operating at temperatures higher than 700 °C, SOFC systems demand well-designed thermal management to prevent hazardous states such as local overheating or suboptimal cooling while sustaining decent electrical efficiency.^6^ These systems face operational challenges arising from exogenous perturbations, frequent power-demand transients, and thermal inertia. These factors complicate the reconciliation of efficiency optimization with operational safety. Hence, the research on SOFC system control and optimization lags behind other fuel cell technologies.^7–10^ Initial research efforts focused on dynamic modeling frameworks to enable real-time control. Gupta et al.^11,12^ applied an adaptive model predictive controller to improve the performance of SOFC system. Gao et al.^13^ developed a quasi-two-dimensional tubular SOFC model with rapid fault-detection capabilities. However, the dependency on precise plant parametrization limited the robustness against inherent plant-model mismatches. Cheddie et al.^14^ advanced this domain through a computationally efficient 21-node dynamic 1D model, though unresolved discrepancies between simulated and actual operating conditions persisted. To address these limitations, modifier adaptation (MA)-based real-time optimization (RTO) algorithms emerged, integrating numerical optimization with plant measurements to systematically counteract model inaccuracies.^15^

The cornerstone of MA RTO lies in its iterative integration of process measurements to refine operational performance, as described in the tutorial literature.^16–19^ As the optimum of SOFC systems is generally determined by the operating boundary, for example, the highest fuel utilization or the highest stack temperature, constraint adaptation (CA) RTO algorithm can be used.^20^ The methodological evolution of CA-RTO in SOFC applications traces back to 2011, when Marchetti et al.^21^ pioneered its implementation using a dual-model framework: a high-fidelity ‘plant’ simulator and a purposefully parametrized ‘inaccurate’ model with deviations in critical parameters. Their work demonstrated CA-RTO’s capacity to drive the plant toward optimum on the constraints despite significant plant-model mismatch. Subsequent experimental validation by Bunin et al.^22^ confirmed CA-RTO’s efficacy in converging SOFC stacks to specified power demands and active constraints using steady-state measurements and static models. de Avila Ferreira et al.^23^ then conducted hardware-in-the-loop validation, applying the hardware of an SOFC stack, a prereformer and an electrical heater with the software to simulate the afterburner and heat exchanger behavior. Their fast CA-RTO algorithm, using transient measurements and dynamic models, reduced optimization settling times from 30 to 120 min (conventional static approaches) to 3–21 min. This advancement was further substantiated through deployment on the BlueGen SOFC system,^24^ validating that fast CA-RTO algorithm can drive the system to its optimum on the constraints and reach the power set points within 15 min.

The previous research considered detailed electrochemical terms in the stack model, which potentially lagged the optimization process with long computation time, and a detailed plate heat exchanger model that might cause temperature-cross problem, destabilizing the optimization process. Furthermore, experimental validations remained confined to controlled laboratory environments, where disturbances were limited to sensor noise—an inadequate proxy for the stochastic perturbations inherent to field deployments. To rigorously assess algorithm maturity for industrial SOFC integration, this study implemented a μ-CHP SOFC system (New Enerday Home750) connected to the regional natural gas infrastructure and residential thermal networks in GazNat, Switzerland. A simplified model with improved reliability was built and validated. Unpredictable external and predictable internal disturbances were present to validate the robustness of fast CA-RTO algorithm. The fast CA-RTO algorithm’s robustness was systematically evaluated under both internal disturbances (e.g., cyclical activation of proportional-integral-derivative (PID) controller) and external perturbations (e.g., gas composition fluctuations), thereby bridging critical gaps between theoretical optimization and real-world operational resilience.

Notably, we specifically used the terms **negatrode** for the fuel electrode and **positrode** for the air electrode, to avoid any potential misunderstanding, since solid oxide cell technology is known for its reversible operation capability.^25^

## Home750 SOFC System

### System Description

The New Enerday Home750 μ-CHP SOFC system comprises six core components: (1) a catalytic partial oxidation reactor (CPOX), (2) an afterburner to burn the unreacted fuel, (3) an air heat exchanger to preheat the sweep air of the stack, (4) an air mixing valve to control the ratio of nonsuperheated air, (5) a water heat exchanger for waste heat recovery, and (6) a stack with power capacity from 550 to 850 W at nominal condition. As is depicted in Figure 1a, natural gas (mainly CH_4_) is partially oxidized in the CPOX with air to maintain the stack negatrode inlet temperature at around 790 °C. The positrode air first passes through the air mixing valve, with a portion of the cold air bypassing the air heat exchanger and instead passing through the startup burner at around 100 °C, while the remaining air is heated by the air heat exchanger. Both air flows are then mixed before entering the stack positrode at around 750 °C. Adjusting the air mixing valve ratio allows for rapid control of the stack inlet air temperature, ensuring a safe stack operating temperature. Unreacted fuel from the negatrode was completely burned with the surplus O_2_ from the positrode in the afterburner, and the exhaust was used as the heating medium to heat up both air and water for waste heat recovery. Five temperatures were measured during the tests, including (1) CPOX outlet gas temperature (*T*_*cpox*_), i.e., stack negatrode inlet temperature, (2) stack inlet air temperature (*T*_*air*_), (3) stack outlet temperature (*T*_*stack*_), (4) afterburner outlet temperature (*T*_*burn*_), and (5) inlet and outlet temperature of water (*T*_*H*_2_ *O*,*in*_ and *T*_*H*_2_ *O*,*out*_). According to Figure 1b, the system was placed in a factory with direct connection to the regional gas grid and the in-house hot water line.

**Figure 1:**
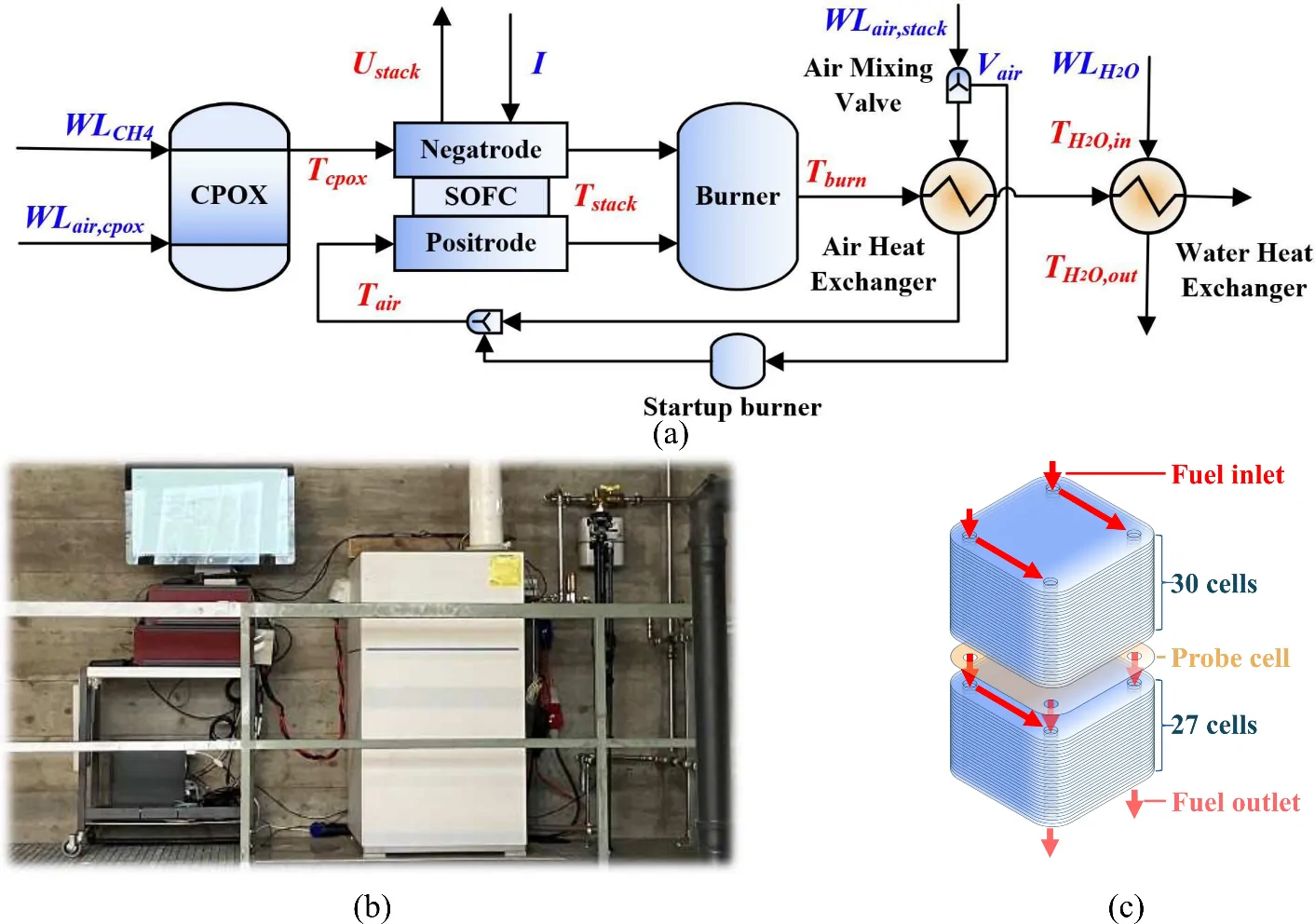
(a) Process flow diagram of the system where the blue symbols are the system inputs and the red symbols stand for the system output measurements, (b) exterior view of the system, (c) the configuration of the stack, which is composed of top half stack with 30 cells closer to the fuel inlet, one probe cell in the middle indicating the gas composition with open circuit voltage (OCV), and bottom half stack with 27 cells. Adapted from ref 26. Copyright 2025 The Authors. Published by Elsevier under a CC BY 4.0 license.

As is shown in Figure 1a, the system comprises five controlled inputs, including the negatrode-related inputs, (1) natural gas blower workload (*WL*_*CH*_4__), (2) CPOX air blower workload (*WL*_*air*,*cpox*_), (3) stack current (*C*), and the positrode-related inputs (4) positrode air blower workload (*WL*_*air*,*stack*_) and (5) air mixing valve ratio (*V*_*air*_). Natural gas blower workload, positrode air blower workload, stack current, and air mixing valve ratio can be directly controlled, while the CPOX air blower workload must be changed via a PID controller due to control system limitation of a commercial product.

#### PID Controllers

The Home750 SOFC system employs multiple PID controllers for operational regulation and safety assurance. During experimental campaigns, noncritical control loops were deactivated, retaining only safety-critical controllers to maintain system integrity. The key PID controllers during the experiments are described below:

- **CPOX AIR PID** changed the CPOX air blower workload to reach the set CPOX outlet temperature. Hence, this PID controller was used to indirectly change the CPOX air blower workload set point during the test.
- **STACK AIR PID** changed the positive electrode air blower workload to maintain the set stack outlet temperature.
- **AIR VALVE PID** changed the air mixing valve ratio to maintain the set stack outlet temperature, which was only activated when the positrode air blower workload reached its lower bound of 28%.
- **H2O PID** changed the water pump workload to maintain the water temperature difference between inlet and outlet at 10 °C.

#### System Disturbances

The system was directly connected to the regional natural gas grid operated by GazNat SA (Aigle, Switzerland), thereby closely emulating real-world operational conditions. The composition fluctuation of the natural gas grid served as an unpredictable external disturbance to the system operation. As is shown in the Supporting Information Figures S3–S7, the CPOX outlet temperature could suffer from temperature fluctuation exceeding 30 °C without any change upon the natural gas blower workload and the CPOX air blower workload, indicating occasional considerable gas composition change in the natural gas grid.

Internal predictable operational disturbance arose from the automated 23-h self-check action of the control subsystem. Every 23 h, the positrode air blower workload and the air mixing valve ratio underwent predefined step perturbations via temporary activation of the **STACK AIR PID** and **AIR VALVE PID** controllers, irrespective of their operational state. This protocol verified the actuator integrity and dynamic response fidelity under load transients. During the RTO test, the 23-h self-check action was interrupted by automatically closing the PID controller, which consequently affected the positrode blower performance curve.

### System Model

A main advantage of the RTO algorithm is the use of a simplified dynamic system model with low computational complexity and good trend description of the real system, where considerable plant-model mismatch was compensated by the algorithm. CPOX, SOFC stack, afterburner, and startup burner were modeled as continuous stirred-tank reactors (CSTR) with spatially homogeneous temperature and composition profiles. The downstream was assumed to exhibit instantaneous equilibrium with bulk reactor conditions, obviating the spatial resolution of transport phenomena. Conventional CSTR implementations include the reaction kinetics equations to determine the mass balance.^24,27–29^ In this study, the reaction kinetic equations were replaced with simplified assumptions, which accelerated the model computation but were still adequate for performance optimization based on the CA-RTO algorithm. The model description with simplification details and the parameter estimation process are elaborated in Supporting Information Parts A and B.

## Real-Time Optimization Approach

As shown in the Supporting Information Part C, the aforementioned simplified model showed similar dynamic trends to the actual system, but a large plant-model mismatch still existed regarding different variables. On the basis of the RTO algorithm, the inaccurate model was applied to optimize the system performance, along with the real-time plant measurements.^16,17^

### Problem Formulation

The steady-state optimization problem for the plant can be formulated as the following nonlinear programming (NLP) problem:^20^

1a $$\underset{u}{\min}\Phi_{p}\left(u\right)≔\phi \left(u,y_{p}\left(u\right)\right)$$

1b $$s.t.H_{p}\left(u\right)≔h\left(u,y_{p}\left(u\right)\right)=0$$

1c $$G_{p}\left(u\right)≔g\left(u,y_{p}\left(u\right)\right)\leq 0$$

1d $$u^{L}\leq u\leq u^{U}$$

where $u\in R^{n_{u}}$ denotes the input variables as an *n*_*u*_-dimensional vector; $y_{p}\left(u\right)\in R^{n_{y}}$ is the steady-state input-output map of the plant; $\phi :R^{n_{u}}\times$ $R^{n_{y}}\to R$ denotes the cost function to be minimized; $h:R^{n_{u}}\times$ $R^{n_{y}}\to R^{n_{h}}$ is the set of process-dependent equality constraints involving the inputs and the outputs; $g:R^{n_{u}}\times$ $R^{n_{y}}\to R^{n_{g}}$ is the set of process-dependent inequality constraints; **u** ^*L*^ and **u** ^*U*^ are the lower and upper bounds on the inputs, respectively. Note that (·)_*p*_ denotes the quantities related to the plant.

In any practical application, the plant variables **y**_*p*_(**u**) can not be accurately known. In this case, an approximate model is used in the form

2a f(u,x,θ)=0

2b y=F(u,x,θ)

where $f\in R^{n_{x}}$ is a set of process model equations including mass and energy balances and thermodynamic relationships, $x\in R^{n_{x}}$ are the state variables, $y\in R^{n_{y}}$ are the output variables predicted by the model, **F** is the input-output mapping model, and $\theta \in R^{n_{\theta }}$ is a set of adjustable model parameters that have been estimated.

For given **u** and **θ**, the solution to problem [Disp-formula eq2a] can be expressed as the steady-state mapping **x** = **ξ** (**u**, **θ**), and the input-output mapping predicted by the model can be written as **y** (**u**, **θ**) = **F**(**ξ** (**u**, **θ**), **u**, **θ**).

Using the model introduced in eq 2a, the solution of the original problem [Disp-formula eq1a] can be changed into the following NLP problem:

3a $$\underset{u}{\min}\Phi \left(u,\theta \right)≔\phi \left(u,y\left(u,\theta \right)\right)$$

3b s.t.H(u,θ)≔h(u,y(u,θ))=0

3c G(u,θ)≔g(u,y(u,θ))≤0

3d $$u^{L}\leq u\leq u^{U}$$

Assuming that the feasible set *U* ≔ {**u**: **H**(**u**, **θ**) = 0; **G**(**u**, **θ**) ≤ 0} is nonempty and compact for **θ** given and that Φ (**u**, **θ**) is continuous on *U*, a minimizing solution, u*, of problem [Disp-formula eq3a] is guaranteed to exist. Moreover, the set of active inequality constraints at u* is denoted by **A** ≔ {*i*: *G*_*i*_(u*, **θ**) = 0, *i* = 1, ..., *n*_*g*_}.

The solutions between problem [Disp-formula eq1a] and problem [Disp-formula eq3a] are different. Internally, no process model can perfectly describe the real system, i.e., Φ ≠ Φ_*p*_, **G** ≠ **G**_*p*_, **H** ≠ **H**_*p*_. Externally, the system operation under real-world condition experiences disturbances. Instead of iteratively updating the process model like the two-step approach,^30,31^ MA RTO is proposed to reach plant optimality by iteratively adapting the NLP problem [Disp-formula eq3a] based on the process model, which can enforce the convergence to the plant optimum in the presence of plant-model mismatch.^32^ MA RTO applies additive correction terms to the objective and constraint functions of the optimization NLP problem [Disp-formula eq3a] based on the process model.^16–19^ In the context of SOFC systems, the plant optimality is expected to be fully determined by active plant constraints, e.g., upper bound of fuel utilization (FU) or BoP component temperatures. Hence, CA-RTO method can be applied, which is a simpler version of MA RTO without estimating the plant gradients.^16,24,27,33^

### Constraint Adaptation-Based RTO

The first principle of the CA-RTO method is to enforce the correct set of active constraints without relying on the model accuracy. CA-RTO method depends on the iterative updates of the NLP problem [Disp-formula eq3a] constraints via the process model. Plant measurements are taken continuously during the optimization to track the active plant constraints.^21,22^ With the set of active constraints unknown during the switch between different operating conditions, feedback control via optimization is implemented, where the optimal inputs are determined by solving the iteratively updated NLP problem [Disp-formula eq3a]. The optimization process generates an *n*_*u*_-dimensional input vector, which results in a minimal cost and *n*_*u*_ active constraints from the collections of *n*_*h*_ + *n*_*g*_ ≥ *n*_*u*_ potential constraints.

At the *k* ^*th*^ RTO iteration, solving the following modified optimization problem generates the optimal inputs:

4a $$u_{k+1}^{*}=\arg\underset{u}{\min}\Phi \left(u\right)$$

4b $$s.t.H\left(u_{k}\right)+\varepsilon_{k}^{H}=0$$

4c $$G\left(u_{k}\right)+\varepsilon_{k}^{G}\leq 0$$

4d $$u^{L}\leq u\leq u^{U}$$

where ***ε***_*k*_^**H**^ are the equality constraint modifiers and ***ε***_*k*_^**G**^ are the inequality constraint modifiers. These modifiers are used to update the model by comparison with the real plant measurements.

Considering that the plant optimum operating point usually lies on the constraints, there is the possibility that **u**_*k*+1_^*^ are in the infeasible region of the plant, which will usually lead to stack degradation or even failure. In this case, constraint adaptation is implemented with exponential filtering of the modifiers or the inputs, which also reduces the impact of measurement noise and provides additional handles for enforcing convergence. When applying exponential filtering to the inputs, the optimized inputs of the next iteration can be defined with the filter diagonal matrix **K** in the interval (0,1]:

5 $$u_{k+1}={\mathrm{Ku}}_{k+1}^{*}+\left(I-K\right)u_{k}$$

where $I\in R^{n_{u}\times n_{u}}$ is the identity matrix.

### Fast Constraint Adaptation

As elaborated by de Avila Ferreira et al.,^24^ problem [Disp-formula eq1a] considers the plant at steady state, where the constraint modifiers are calculated as the difference between the steady-state plant measurements (**H**_*p*_ (**u**_*k*_), **G**_*p*_ (**u**_*k*_)), and the model predictions (**H** (**u**_*k*_), **G** (**u**_*k*_)). This solution is feasible upon the convergence of the iterative scheme, but the time between two consecutive RTO iterations depends on the time duration toward steady state of the system. As is indicated in Supporting Information Part C Table S2, the system thermal stabilization time caused by the change of different inputs ranges from 2 to 28 h. In contrast, the electrical response of the system can be short based on the ramping speed of the current and the fuel flow rates. In this case, using steady-state constraint adaptation cannot ensure an efficient operating condition switching.

A simplified model enables the rapid computation of dynamic behavior. Combined with the transient plant measurements, a fast CA-RTO approach was proposed to cope with the different time scales between the fast power demand change and the slow thermal inertia.^27^ This approach estimates the plant steady state from transient measurements based on the dynamic model whose outputs at steady state stay consistent with the steady-state model, as expressed below:

6a ẋ(t)=f(x(t),u(t),θ)

6b $$x\left(0\right)=x_{0}$$

6c y(t)=F(x(t),u(t),θ)

Given the predicted dynamic model constraint values, **H** ^*dyn*^ (*t*_*k*_) ≔ **h** (**u**_*k*_, **y** (*t*_*k*_)) and **G** ^*dyn*^ (*t*_*k*_) ≔ **g** (**u**_*k*_, **y** (*t*_*k*_)), the plant steady-state constraint values at time *t*_*k*_ are estimated as follows:

7a $${Ĥ}_{p}\left(u_{k}\right)=H_{p}^{dyn}\left(t_{k}\right)+\left(H\left(u_{k}\right)-H^{dyn}\left(t_{k}\right)\right)$$

7b $${Ĝ}_{p}\left(u_{k}\right)=G_{p}^{dyn}\left(t_{k}\right)+\left(G\left(u_{k}\right)-G^{dyn}\left(t_{k}\right)\right)$$

Hence the modifiers shown in problem [Disp-formula eq4a] can be calculated as below:

8a $${\mathrm{\varepsilon ̂}}_{k}^{H}≔{Ĥ}_{p}\left(u_{k}\right)-H\left(u_{k}\right)=H_{p}^{dyn}\left(t_{k}\right)-H^{dyn}\left(t_{k}\right)$$

8b $${\mathrm{\varepsilon ̂}}_{k}^{G}≔{Ĝ}_{p}\left(u_{k}\right)-G\left(u_{k}\right)=G_{p}^{dyn}\left(t_{k}\right)-G^{dyn}\left(t_{k}\right)$$

where **H** ^*dyn*^ (*t*) → **H**(**u**_*k*_) and **G** ^*dyn*^ (*t*) → **G**(**u**_*k*_) when *t* → ∞, transforming the fast CA-RTO approach into the steady state CA-RTO approach.

The fast CA-RTO approach is illustrated in Figure 2. The time interval between two iterations in steady-state CA-RTO scheme, δ = *t*_*k*+1_ – *t*_*k*_, is completely determined by the time duration of switching between two steady states, which is longer than 12 h in our case. However, the time interval in the fast CA-RTO scheme is determined by the operator, which can ideally be the minimal value between the time for optimization problem [Disp-formula eq4a] computation and the limitation of input ramping speed, i.e., δ = min [δ_*NLP*_, **δ**_*input*_].

**Figure 2:**
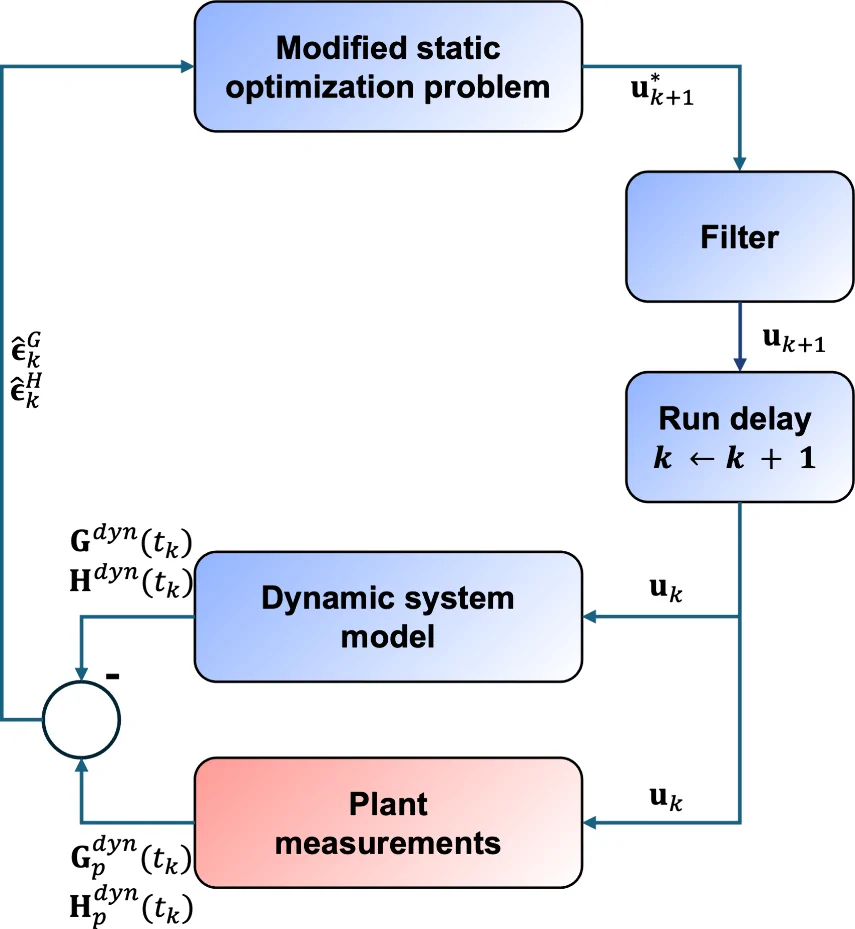
Fast CA-RTO scheme based on the plant transient measurements and a simplified dynamic model.

## Experimental Setup

The SOFC system was controlled by an application programming interface (API) gateway provided by New Enerday GmbH. The fast CA-RTO algorithm was implemented in MATLAB. Considering both electrical efficiency and CHP efficiency as the cost functions, the plant optimization problem (eqs 1a) is formulated as follows:

9 $$\begin{array}{c} \underset{u}{\max}\eta_{el}=\frac{P_{el}-P_{loss}}{Q_{CH_{4}}\left(WL_{CH_{4}}\right)\times LHV_{CH_{4}}},\mathrm{or},\eta_{CHP}=\frac{P_{el}-P_{loss}+q_{H_{2}O}C_{p,H_{2}O}\times \left(T_{H_{2}O,\mathrm{out}}-T_{H_{2}O,\mathrm{in}}\right)}{Q_{CH_{4}}\left(WL_{CH_{4}}\right)\times LHV_{CH_{4}}}\\ \mathrm{s.t.}P_{el}=P_{el}^{set}\left[W\right]\\ 700\leq T_{cpox}\leq 850{[}^{o}C]\\ 755\leq T_{stack}\leq 800{[}^{o}C]\\ 42\leq U_{stack}\leq 60\left[V\right]\\ 710\leq T_{burn}\leq 900{[}^{o}C]\\ 720\leq T_{air}\leq 810{[}^{o}C]\\ FU\leq 79\%\\ AU\leq 25\%\\ COR\leq 0.75\\ 50\%\leq WL_{CH_{4}}\leq 100\%\\ 60\%\leq WL_{air,\mathrm{cpox}}\leq 80\%\\ 28.5\%\leq WL_{air,\mathrm{stack}}\leq 49.5\%\\ 0.1\leq C\leq 21\left[A\right]\\ 10\%\leq V_{air}\leq 30\% \end{array}$$

where *LHV*_*CH*_4__ is the lower heating value of CH_4_, *q*_*H*_2_ *O*_ is the flow rate of water, *C*_*p*,*H*_2_ *O*_ is the heat capacity of water, *T*_*H*_2_ *O*,*out*_ is the outlet water temperature, *T*_*H*_2_ *O*,*in*_ is the inlet water temperature, *T*_*cpox*_ is the CPOX outlet temperature, *T*_*stack*_ is the stack outlet temperature, *U*_*stack*_ is the stack voltage, *T*_*burn*_ is the afterburner outlet temperature, *T*_*air*_ is the inlet positrode air temperature, *FU* is the stack fuel utilization, *AU* is the air utilization, *COR* is the carbon to oxygen ratio of the CPOX, and *P*_*loss*_ is the power consumption of the inverter, blowers and pumps, as is expressed below:

10 $$P_{loss}=P_{conv}+P_{\max,{\mathrm{CH}}_{4}}\times WL_{CH_{4}}+P_{\max,\mathrm{cpox}}\times WL_{air,\mathrm{cpox}}+P_{\max,\mathrm{pos}}\times WL_{air,\mathrm{stack}}+P_{\max,H_{2}O}\times WL_{H_{2}O}$$

where *P*_*conv*_ is the power loss from the converter, fixed at 8 W, *P*_*max*,*CH*_4__ is the maximal power of the natural gas blower, fixed at 9.6 W, *P*_*max*,*cpox*_ is the maximal power of the CPOX air blower, fixed at 16.32 W, *P*_*max*,*pos*_ is the maximal power of the positrode air blower, fixed at 70 W, *P*_*max*,*H*_2_ *O*_ is the maximal power of the water pump, fixed at 50 W, and *WL*_*H*_2_ *O*_ is the water pump workload.

Considering the electrical efficiency penalty due to the CPOX, the stack efficiency was calculated as a key indicator of the system performance and the *FU* considers the fuel consumption of the CPOX, as expressed below:

11a $$\eta_{el,\mathrm{stack}}=\frac{P_{el}}{\left(Q_{CH_{4}}\left(WL_{CH_{4}}\right)-0.5\times Q_{O_{2},\mathrm{cpox}}\left(WL_{air,\mathrm{cpox}}\right)\right)\times LHV_{CH_{4}}}$$

11b $$\mathrm{FU}=\frac{C\times N_{cell}÷8F}{Q_{CH_{4}}\left(WL_{CH_{4}}\right)-0.5\times Q_{O_{2},\mathrm{cpox}}\left(WL_{air,\mathrm{cpox}}\right)}$$

where *Q*_*CH*_4__ is the CH_4_ flow rate converted from *WL*_*CH*_4__ and *Q*_*O*_2_,*cpox*_ is the O_2_ flow rate converted from *WL*_*air*,*cpox*_, which are elaborated in Supporting Information Part A. The conversion between the natural gas blower workload and the flow rate was calibrated against an in-house gas meter; the raw calibration data are provided in the Supporting Information Excel datasheet.

The proposed inequality constraints were set based on the system limitation proposed by New Enerday GmbH, as shown in Supporting Information Part E Table S6. Any violation of the system limitations would trigger an emergency shutdown. Hence, the temperature and voltage constraints proposed in problem [Disp-formula eq9] were very conservative to avoid any potential risks of shutdowns. In our previous research regarding the SOFC system characterization, we determined that the FU threshold to avoid fuel starvation is 81%.^26^ In this case, the FU constraint was limited to 79% of the total. Once the positrode air blower workload reached the maximal or minimal values set in the control subsystem, **STACK AIR PID** and **AIR VALVE PID** were automatically activated to interrupt the RTO test. In this case, the upper and lower bounds of *WL*_*air*,*stack*_ were set at 28.5% and 49.5%, respectively. The lower bound of the CPOX air blower workload was set at 60% to ensure that the OCV was lower than 1.07 V, which guaranteed that there was no carbon deposition inside the CPOX or the stack.

### Control Strategies

Home750 SOFC system was initialized at the nominal operating condition with power output around 790 W. When applying the fast CA-RTO approach to the system, the power targets followed the pattern, 840 W → 800 W → 760 W → 720 W → 800 W. The power target returned to 800 W from 720 W in the end to test the algorithm’s reproducibility at doubled power change.

As the system was continuously disturbed by the composition fluctuation of the natural gas from the grid, the CA-RTO algorithm was always activated during each power target to validate its robustness against external perturbation.

All the inputs except for the CPOX air blower workload were directly changed. CPOX air blower workload was indirectly controlled by the **CPOX AIR PID**, where the PID’s temperature target was changed iteratively to reach the specified CPOX air blower workload at fixed natural gas blower workload. The applied current fluctuated between the set point and the value with 0.1 A lower than the set point, corresponding to a power output fluctuation of around 5 W. Considering the sampling rate of the data acquisition system was 20 s, the measurement of the system power output was the average between the maximum power and minimum power of the last 6 samples. Similarly, the temperature measurements of different components were the average values of the last 6 samples. To characterize the impact of different numbers of controlled inputs within a limited time window, three sets of inputs were tested to validate performance improvements, including:

- 2-input case: (1) natural gas blower workload (*WL*_*CH*_4__) and (2) current (*C*).
- 3-input case: (1) natural gas blower workload (*WL*_*CH*_4__), (2) CPOX air blower workload (*WL*_*air*,*cpox*_), and (3) current (*C*).
- 5-input case: (1) natural gas blower workload (*WL*_*CH*_4__), (2) CPOX air blower workload (*WL*_*air*,*cpox*_), (3) positrode air blower workload (*WL*_*air*,*stack*_), (4) current (*C*), and (5) air mixing valve ratio (*V*_*air*_).

#### Asynchronous Strategy

According to instructions from the manufacturer, New Enerday GmbH, the operator could directly change the set point of natural gas blower workload, CPOX air blower workload, and current without limitation. The change of heat-exchange-loop-related inputs, positrode air blower workload, and air mixing valve ratio must change at a rate lower than 1% every 9 min. Hence, we applied an asynchronous control strategy here, where the time interval between two iterations was set at 3 min. The three unlimited inputs (*WL*_*CH*_4__, *WL*_*air*,*cpox*_, *C*) were controlled every iteration, while the two slow responding inputs (*WL*_*air*,*stack*_, *V*_*air*_) were changed by 1% every 3 iterations.

An asynchronous control strategy was applied to the tests in all circumstances with both electrical efficiency and CHP efficiency as cost functions.

#### Synchronous Strategy

When optimizing the CHP efficiency, the mismatch among the ramping speeds of the input variables may cause an overheating problem, due to high thermal inertia. In this case, the synchronous control strategy was applied to keep the ramping speed of all the inputs consistent with the slowest responding inputs, i.e., the positrode air blower workload and the air mixing valve ratio at 1% per 9 min. Hence, the time interval between two RTO iterations was set to 9 min, and the inputs were controlled as below:

12a $$WL_{CH_{4}}\left(t_{k+1}\right)=\{\begin{array}{cc} WL_{CH_{4}}^{*}\left(t_{k+1}\right), & \left|\Delta WL_{air,\mathrm{stack}}\right|\leq 1\%\wedge \left|\Delta V_{air}\right|\leq 1\%\\ WL_{CH_{4}}\left(t_{k}\right)+\frac{WL_{CH_{4}}^{*}\left(t_{k+1}\right)-WL_{CH_{4}}\left(t_{k}\right)}{\max\left[\left|\Delta WL_{air,\mathrm{stack}}\right|,\left|\Delta V_{air}\right|\right]\times 100}, & \left|\Delta WL_{air,\mathrm{stack}}\right|>1\%\vee \left|\Delta V_{air}\right|>1\% \end{array}$$

12b $$WL_{air,\mathrm{cpox}}\left(t_{k+1}\right)=\{\begin{array}{cc} WL_{air,\mathrm{cpox}}^{*}\left(t_{k+1}\right), & \left|\Delta WL_{air,\mathrm{stack}}\right|\leq 1\%\wedge \left|\Delta V_{air}\right|\leq 1\%\\ WL_{air,\mathrm{cpox}}\left(t_{k}\right)+\frac{WL_{air,\mathrm{cpox}}^{*}\left(t_{k+1}\right)-WL_{air,\mathrm{cpox}}\left(t_{k}\right)}{\max\left[\left|\Delta WL_{air,\mathrm{stack}}\right|,\left|\Delta V_{air}\right|\right]\times 100}, & \left|\Delta WL_{air,\mathrm{stack}}\right|>1\%\vee \left|\Delta V_{air}\right|>1\% \end{array}$$

12c $$WL_{air,\mathrm{stack}}\left(t_{k+1}\right)=\{\begin{array}{cc} WL_{air,\mathrm{stack}}^{*}\left(t_{k+1}\right), & \left|\Delta WL_{air,\mathrm{stack}}\right|\leq 1\%\\ WL_{air,\mathrm{stack}}\left(t_{k}\right)+\mathrm{sign}\left(\Delta WL_{air,\mathrm{stack}}\right)\times 1\%, & \left|\Delta WL_{air,\mathrm{stack}}\right|>1\% \end{array}$$

12d $$C\left(t_{k+1}\right)=\{\begin{array}{cc} C^{*}\left(t_{k+1}\right), & \left|\Delta WL_{air,\mathrm{stack}}\right|\leq 1\%\wedge \left|\Delta V_{air}\right|\leq 1\%\\ C\left(t_{k}\right)+\frac{C^{*}\left(t_{k+1}\right)-C\left(t_{k}\right)}{\max\left[\left|\Delta WL_{air,\mathrm{stack}}\right|,\left|\Delta V_{air}\right|\right]\times 100}, & \left|\Delta WL_{air,\mathrm{stack}}\right|>1\%\vee \left|\Delta V_{air}\right|>1\% \end{array}$$

12e $$V_{air}\left(t_{k+1}\right)=\{\begin{array}{cc} V_{air}^{*}\left(t_{k+1}\right), & \left|\Delta V_{air}\right|\leq 1\%\\ V_{air}\left(t_{k}\right)+\mathrm{sign}\left(\Delta V_{air}\right)\times 1\%, & \left|\Delta V_{air}\right|>1\% \end{array}$$

12f $$\Delta WL_{air,\mathrm{stack}}=WL_{air,\mathrm{stack}}^{*}\left(t_{k+1}\right)-WL_{air,\mathrm{stack}}\left(t_{k}\right)$$

12g $$\Delta V_{air}=V_{air}^{*}\left(t_{k+1}\right)-V_{air}\left(t_{k}\right)$$

where (·)* denotes the optimal input variable, *ΔWL*_*air*,*stack*_ is the difference of the positrode air blower workload between the optimal value at RTO iteration *k* + 1 and the set point at RTO iteration *k*, *ΔV*_*air*_ is the difference of the air mixing valve ratio between the optimal value at RTO iteration *k* + 1 and the set point at RTO iteration *k*.

The synchronous control strategy was tested once with CHP efficiency as the cost function in the 5-input case.

## Results and Discussion

Multiple plant variables were recorded during the experiments, which are comprehensively archived in the Supporting Information Part E. The following variables are presented to analyze the dynamic behavior of the system and performance of the algorithm:

- Inputs - natural gas blower workload, CPOX air blower workload, positrode air blower workload, current, air mixing valve ratio, and water pump workload.
- Key indicators: FU, stack voltage, electrical efficiency, CHP efficiency, OCV, power output, and stack efficiency.
- Temperatures: CPOX outlet temperature, stack outlet temperature, and stack inlet air temperature.

### Electrical Efficiency as Cost Function

#### Two-Input and Three-Input Cases

In the cases of 2 inputs and 3 inputs controlled for electrical efficiency optimization, each power target experienced 40 RTO iterations with a time interval of 3 min, corresponding to around 2 h. An asynchronous control strategy was applied, and the inputs are shown in Figure 3. The key indicators and the temperatures are shown in Figure 4. The electrical efficiencies and the active constraints at different operating conditions are summarized in Table 1.

**Figure 3:**
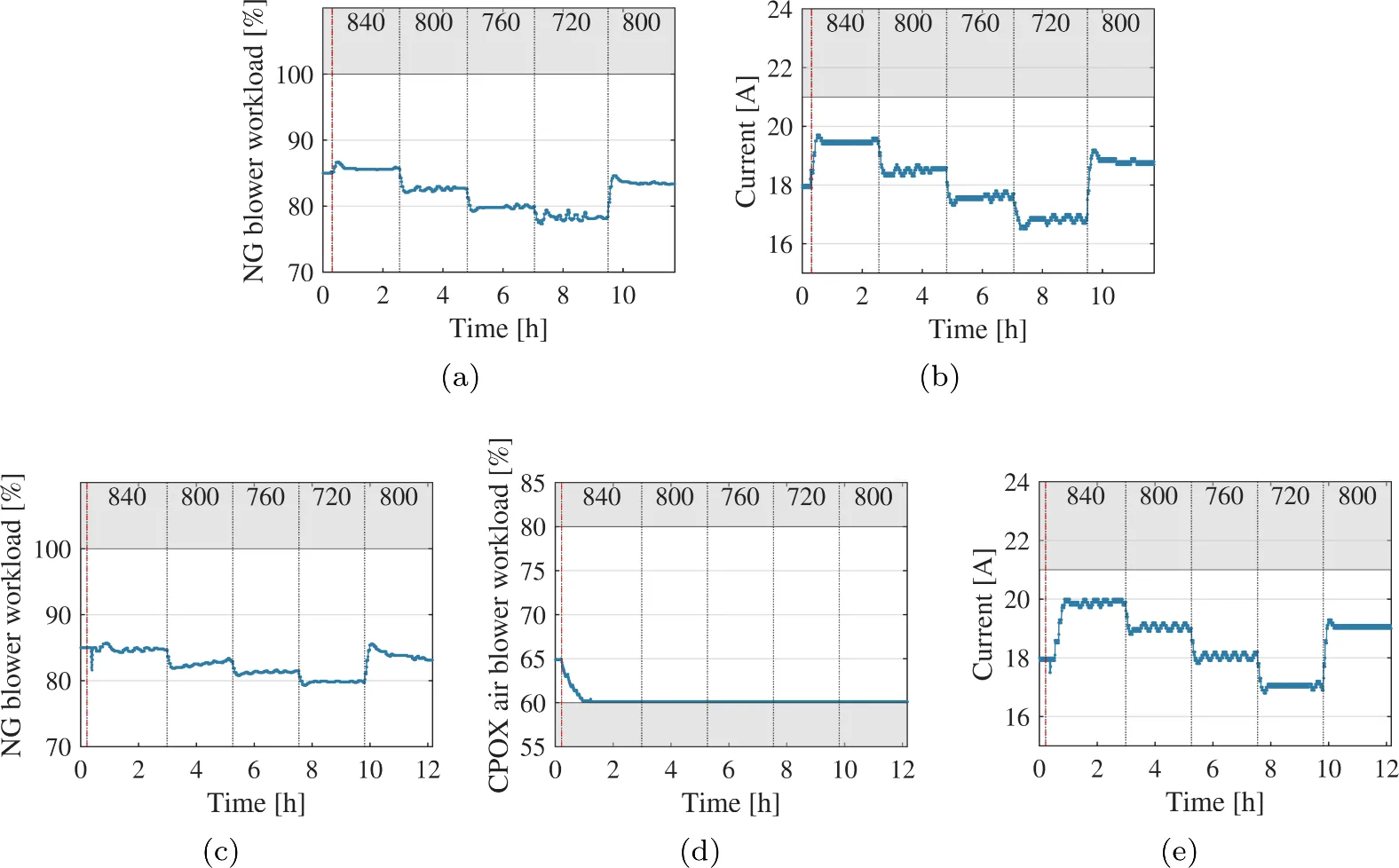
Inputs recording of (a-b) the 2-input case, and (c-e) the 3-input case with electrical efficiency as the cost function, where the number on the top indicates power target and the red dashed line indicates the time of activating CA-RTO algorithm.

**Figure 4:**
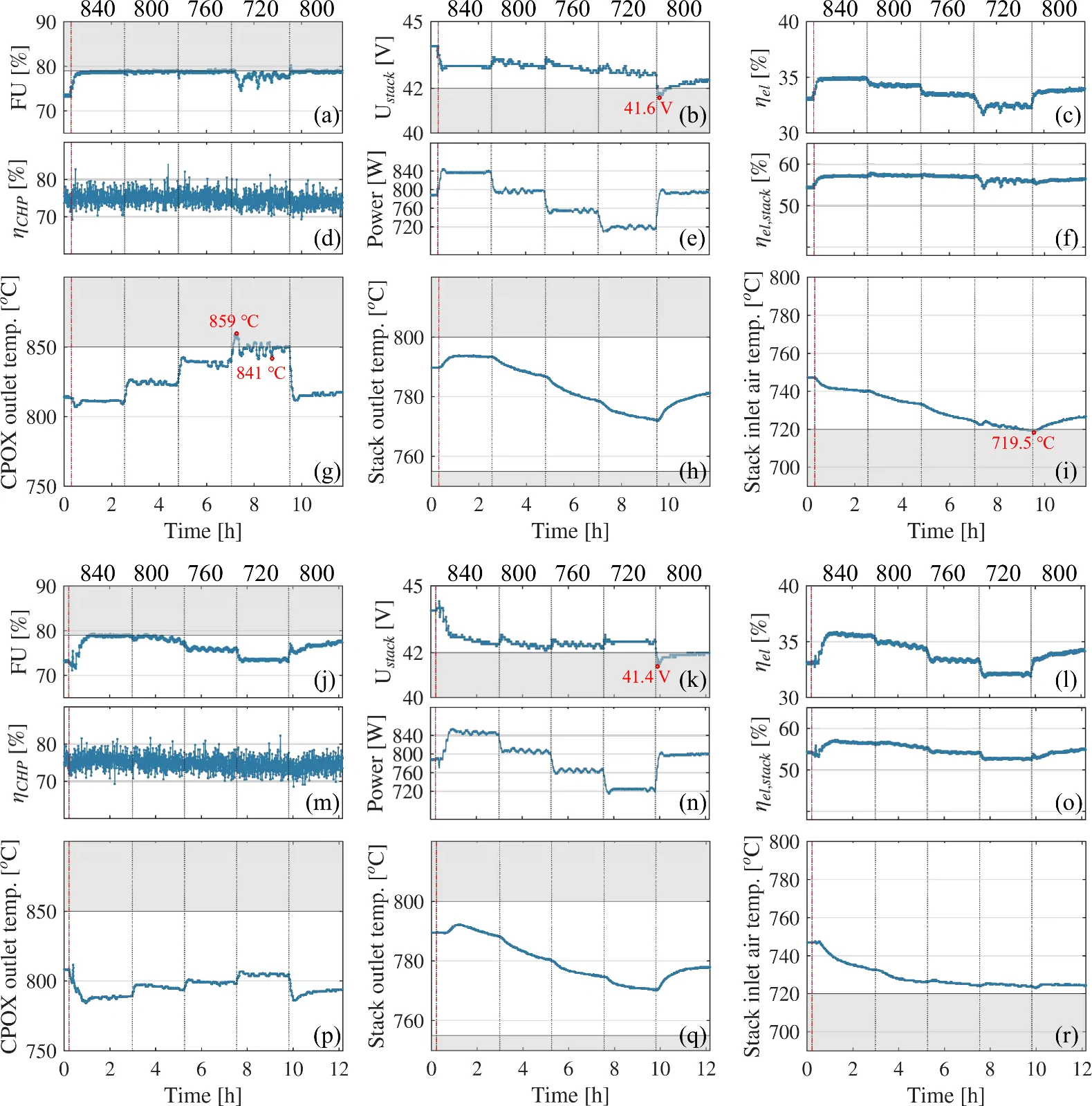
Key indicators and BoP temperature of (a-i) 2-input case and (j-r) 3-input case with electrical efficiency as cost function, where the number on the top indicates power target and the red dashed line indicates the time of activating CA-RTO algorithm.

**Table 1:** Electrical Efficiency Comparison between Different Cases and the Active Constraints at Each Power Target^a^

		**Initial**	**840 W**	**800 W**	**760 W**	**720 W**	**800 W**
**2-input**	**Electrical efficiency**	33.0%	34.9%	34.3%	33.4%	32.4%	33.8%
	**Stack efficiency**	54.2%	57.2%	57.3%	57.0%	56.1%	56.3%
	**Active constraints**	-	*P*_*el*_	*P*_*el*_	*P*_*el*_	*P*_*el*_	*P*_*el*_
		-	*FU* ^*U*^	*FU* ^*U*^	*FU* ^*U*^	*T*_*cpox*_^*U*^ → *T*_*air*_^*L*^	*FU* ^*U*^
**3-input**	**Electrical efficiency**	33.0%	35.6%	34.6%	33.3%	32.1%	34.1%
	**Stack efficiency**	54.1%	56.6%	55.6%	54.2%	52.7%	54.6%
	**Active constraints**	-	*P*_*el*_	*P*_*el*_	*P*_*el*_	*P*_*el*_	*P*_*el*_
		-	*WL*_*air*,*cpox*_^*L*^	*WL*_*air*,*cpox*_^*L*^	*WL*_*air*,*cpox*_^*L*^	*WL*_*air*,*cpox*_^*L*^	*WL*_*air*,*cpox*_^*L*^
		-	*FU* ^*U*^	*FU* ^*U*^ → *U*_*stack*_^*L*^	*U*_*stack*_^*L*^	*U*_*stack*_^*L*^	*U*_*stack*_^*L*^
**5-input**	**Electrical efficiency**	33.1%	36.3%	34.8%	33.5%	32.2%	35.7%
	**Stack efficiency**	53.9%	58.4%	55.6%	54.6%	59.1%	59.0%
	**Active constraints**	-	*P*_*el*_	*P*_*el*_	*P*_*el*_	*P*_*el*_	*P*_*el*_
		-	*WL*_*air*,*cpox*_^*L*^	*WL*_*air*,*cpox*_^*L*^	*WL*_*air*,*cpox*_^*L*^	*WL*_*air*,*cpox*_^*L*^	*WL*_*air*,*cpox*_^*L*^
		-	*WL*_*air*,*stack*_^*L*^	*WL*_*air*,*stack*_^*L*^	*WL*_*air*,*stack*_^*L*^	*WL*_*air*,*stack*_^*L*^	*WL*_*air*,*stack*_^*L*^
		-	*FU* ^*U*^	*FU* ^*U*^	*V*_*air*_^*L*^	*V*_*air*_^*L*^	*FU* ^*U*^
		-	*T*_*stack*_^*U*^	*V*_*air*_^*L*^	*U*_*stack*_^*L*^	*FU* ^*U*^	*T*_*stack*_^*U*^

a Where (·)^*U*^ denotes the upper bound and (·)^*L*^ denotes the lower bound.

Each power target was reached within 15 min, where a higher efficiency improvement was observed with more inputs controlled, and the system constraints were respected. For the inputs, the current and natural gas blower workload of both cases showed similar trends to follow the switch of the power target. The CPOX air blower workload was always maintained at its lower bound during different power targets. This action was foreseen since a smaller CPOX air flow rate implied less fuel preburned in the CPOX and less power consumption from the CPOX air blower workload, which hence increased the system electrical efficiency. Lower CPOX air blower workload was preferred in general, which was evidenced by a test with its lower bound at 50%, as is indicated in Supporting Information Part E Figure S20. In Supporting Information Part E Figure S21, the electrical efficiency was increased from 33% to 35.8% at the same power output of 790 W. The consequence was to approach the upper bound of COR and result in an OCV at around 1.1 V, which was confirmed by New Enerday GmbH to be carbon-deposition-risky. In Figures 3c and 3e, the manipulation was delayed when activating the RTO algorithm, because the change of CPOX air blower workload must be first finished via the **CPOX AIR PID**.

The system efficiency improvement was strongly affected by the system thermal inertia. After activating the CA-RTO algorithm and setting the power target at 840 W, the system efficiency increased from 33% to 35.7% in the 3-input case, while it was increased to 34.8% in the 2-input case. The higher improvement of the 3-input case benefited from the lower CPOX air blower workload, which hence reduced the amount of fuel required for power generation. Comparing Figure 4g-i with Figure 4p-r, the suppressed preburning in the CPOX lowered the temperatures of the whole system. The only exception concerned the stack inlet air temperature at the power target of 720 W, caused by unexpected gas grid fluctuation. As shown in Figure 4g, the CPOX temperature at the power target of 720 W reached the upper bound of 850 °C and fluctuating between 841 and 859 °C with time. However, the CPOX outlet temperature was maintained by iteratively changing the natural gas blower workload as validated by recording of the input (Figure 3a) and FU (Figure 4a). Further evidence of grid gas composition fluctuations emerged when analyzing CHP optimization results in the following section. Briefly, the upper constraint on CPOX outlet temperature remained inactive despite identical input parameters being applied. This observation suggested inherent variability in feed gas properties that standard control parameters fail to capture. Due to the higher stack outlet temperature at 720 W, the 2-input case showed a slightly higher electrical efficiency of 32.4% compared with the 3-input case of 32.1%. Combining with the close electrical efficiency of two cases at 760 W, a clear trade-off was observed between performance improvement from the reduced natural gas injection and the penalty from the corresponding system temperature reduction. The system did not reach the steady state within around 2 h, and hence, the possibility that the electrical efficiency of 3-input case at all power targets might surpass that of 2-input case could not be verified, as the constraint modifier values were still slowly changing with time.

As shown in Table 1, the stack efficiency of the 2-input case at different power targets was higher than that of the 3-input case. Meanwhile, the 2-input case showed steady stack efficiencies between 56% and 57%, compared with the initial stack efficiency at 54.2%. The stack efficiencies of the 3-input case differed greatly at different power targets. Hence, due to the complexity brought by the CPOX and the high thermal inertia, the 2-input strategy can be chosen for better stack efficiency and less risk of carbon deposition, while the 3-input strategy is better at overall system performance improvement, especially at high-power targets.

The number of active constraints was equivalent to the number of controlled inputs, confirming that CA-RTO was sufficient to reach the optimum, since activation of these constraints sets the degree of freedom given by the amount of controlled inputs. The constraints on electrical power were active for all power set points in both cases. In the 2-input case, the constraint on FU was active at high powers (840, 800, 760 W), but at the lower power of 720 W, the constraint on the CPOX outlet temperature became more restrictive due to the high CPOX air flow rate, and must therefore be activated. Simultaneously, the stack inlet air temperature reached slightly below the lower bound (719.5 °C) in the end at 720 W due to high thermal inertia. After reaching the steady state at 720 W, the stack inlet air temperature constraint was assumed to be active instead of the CPOX outlet temperature, because a higher fuel flow rate was implemented to maintain the stack inlet air temperature, which decreased the CPOX outlet temperature. In the 3-input case, a similar constraint migration was also observed between the constraints on FU and stack voltage, where the constraint on the CPOX air blower workload was always active for all power targets. At the first two power targets (840 and 800 W), the upper bound of FU was active, while the constraint on stack voltage became critical and was activated at around 4 h, as shown in Figure 4j. Afterward, the lower bound of stack voltage was active at lower powers and after switching back to 800 W from 720 W. Due to the high thermal inertia of the system, the stack outlet temperature slowly and continuously decreased with the power target fixed at the first 800, 760, and 720 W, which decreased the stack voltage until reaching the lower bound. To satisfy the voltage constraint, the algorithm increased the natural gas blower workload to maintain the stack voltage at its lower bound of 42 V, which simultaneously decreased the FU. Because the stack temperature did not return to a higher level in the last power target of 800 W due to thermal inertia, the stack voltage lower bound, instead of the FU upper bound, was still active. For 2- and 3-input cases, stack voltage dropped to 41.6 and 41.4 V, respectively, when switching from 720 to 800 W power target. This was caused by the faster ramping rate of current and the slower ramping rate of NG blower workload, as shown in Figure 3

In summary, with a limited number of controlled inputs, the CA-RTO algorithm reached the power target within 15 min, which can be accelerated by reducing the iteration interval, for example, 5 min with a 1 min iteration interval. The same number of active constraints as controlled inputs confirmed the adequacy of the algorithm for reaching the plant optimum. The algorithm was robust enough to cope with external disturbances in the natural gas grid. Thermal inertia still played an important role in the optimization, leading to active constraint migration during the operation and different sets of active constraints after switching back to the same power output. At most of the power targets, the plant had the same sets of active constraints as the model optimum shown in Supporting Information Part D Tables S3–S4. Exceptions occurred at 720 W for the 2-input case and 800 to 720 W for the 3-input case, where the final plant optimum did not lie on the same constraints as the models. However, the CA-RTO algorithm still pushed the plant operating conditions to the constraint, achieving the optimal electrical efficiency.

#### Five-Input Case

Comparing 2-input and 3-input cases, the addition of heat-exchange-loop inputs, positrode air blower workload, and air mixing valve ratio prolonged the stabilization time of the system. Hence, each power target experienced 180 iterations with a time interval of 3 min, corresponding to around 9 h at each power target. Asynchronous control strategy was applied, and the 23-h self-check functionality was triggered twice during the test.

At 840 W, the electrical efficiency was increased to 36.3% from 33.1% at the initial condition compared with that of 2-input (34.9%) and 3-input (35.6%) cases, and the power target was reached within 15 min. As shown in Table 1, the five constraints were active, indicating that the algorithm reached the plant optimum with 5 degrees of freedom restricted. The behavior of the CPOX air blower workload, the current, and the FU followed the same logic mentioned in the 3-input case. With the FU reaching the upper bound, the best way to further improve the electrical efficiency was to increase the stack temperature, which increased the stack voltage and, hence, decreased the final amount of fuel injected by maintaining the FU at its upper bound. The algorithm successfully recognized this and forced the positrode air blower workload to its lower bound with a lower blower power consumption, which also increased the stack inlet air temperature and the stack outlet temperature. The air mixing valve ratio was reduced simultaneously to ensure that the stack outlet temperature would not exceed the upper bound. In Figure 6h, the upper bound of the stack outlet temperature was not completely active due to thermal inertia. It could be predicted that the stack outlet temperature would be active after the system reached the final steady state based on Supporting Information Part E Figure S24.

The first 800 W power target was reached in 4 iterations, and the system experienced the first 23-h self-check at the beginning and an unexpected natural gas grid fluctuation at the end. The stack outlet temperature and the stack inlet air temperature were reduced due to the lower fuel injection, and consequently, the constraint on the air mixing valve ratio was active to maintain the system temperature, which replaced the active constraint on the stack outlet temperature at 840 W. The 23-h self-check at the beginning led to the spikes of positrode air blower workload and air mixing valve ratio in Figure 5. The control system recognized the sudden change and restored the positrode air blower workload and the air mixing valve ratio. The 23-h self-check created a ‘clogging-releasing’ phenomenon in the system, which changed the air pressure of both CPOX and positrode in Supporting Information Part E Figure S19. This did not affect the CPOX air blower, but the performance curve of the positrode air blower was changed. Nevertheless, the change was not significant enough to affect the plant optimum, but caused the opposite trend between the model and the system, as shown in Supporting Information Part E Figure S23.

**Figure 5:**
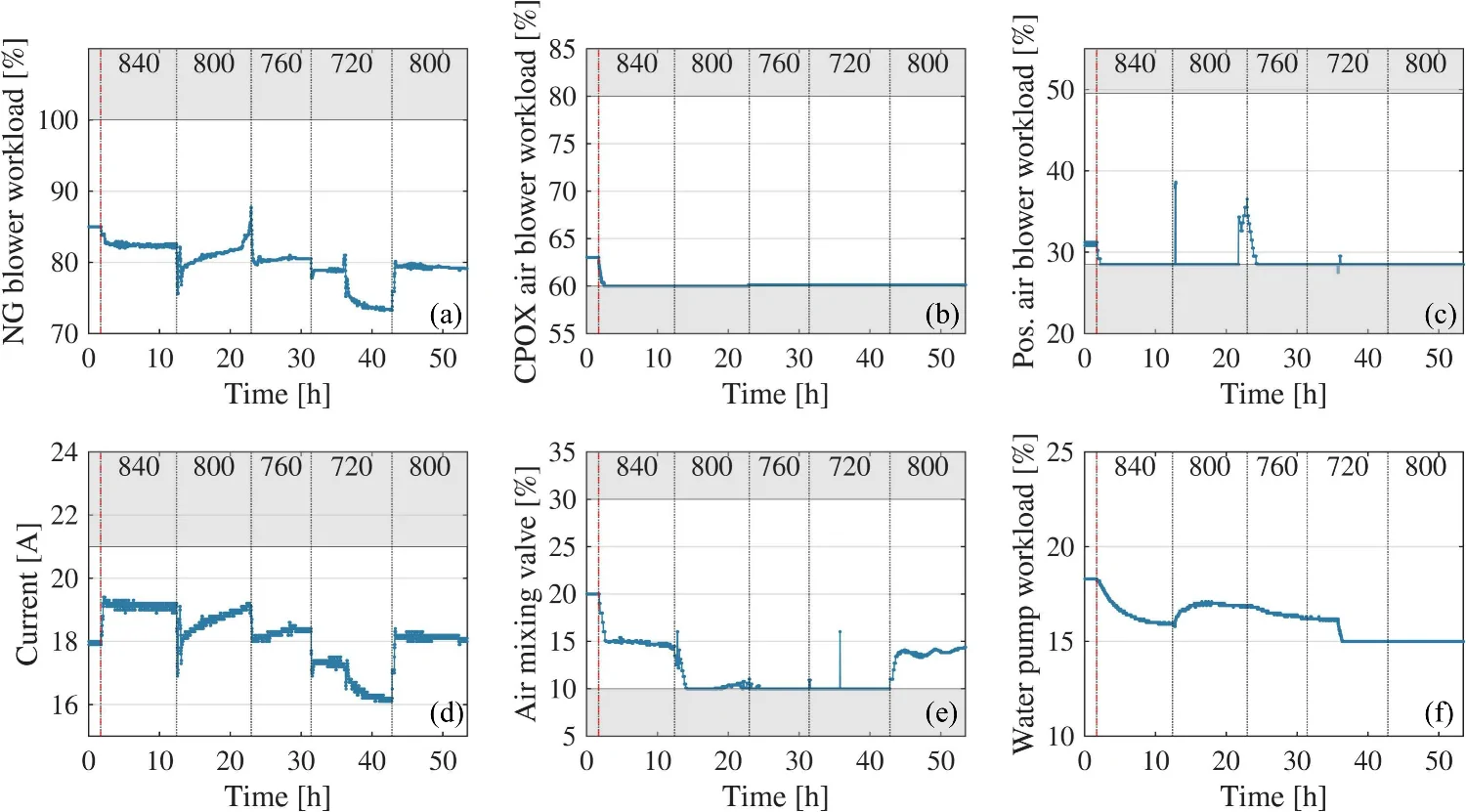
Inputs recording of 5-input case with electrical efficiency as cost function, where the number on the top indicates power target and the red dashed line indicates the time of activating CA-RTO algorithm: (a) natural gas blower workload, (b) CPOX air blower workload, (c) positrode air blower workload, (d) current, (e) air mixing valve ratio, and (f) water pump workload.

At around 21 h, close to the end of the 800 W power target, a sudden increase in the natural gas blower workload was triggered to cope with the natural gas grid fluctuation. This was validated by the OCV recording of the probe cell in Figure 6d. The OCV was maintained at 1.06 V with a fixed CPOX air flow rate and an increased natural gas flow rate, indicating the decreased fuel concentration. The algorithm was not able to reach the optimum with the modifiers under undisturbed conditions. Consequently, the modifier values were reinitialized and the power target was switched to 760 W.

**Figure 6:**
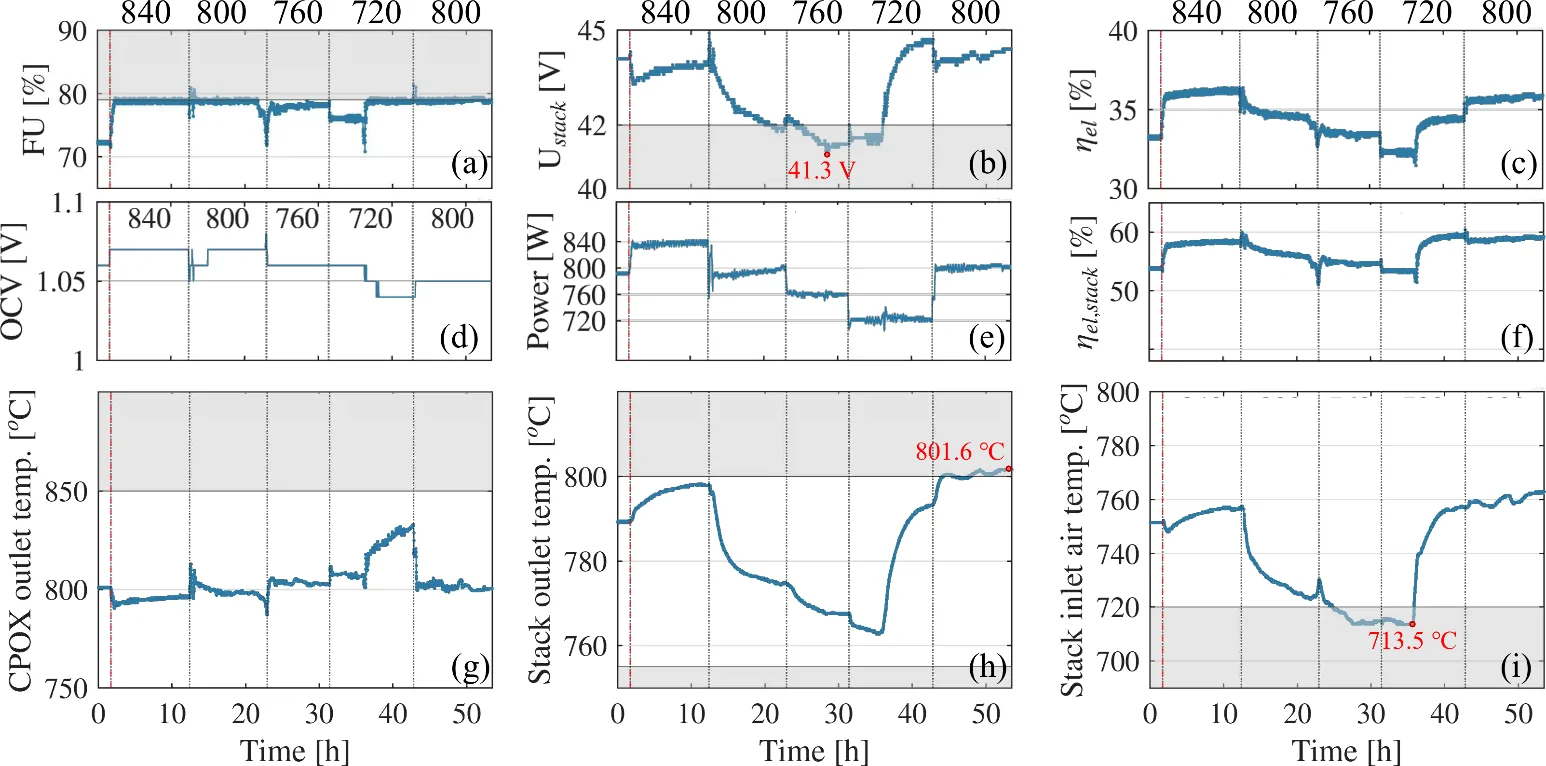
Key indicators and BoP temperature of 5-input case with electrical efficiency as cost function, where the number on the top indicates power target and the red dashed line indicates the time of activating CA-RTO algorithm: (a) FU, (b) stack voltage, (c) electrical efficiency, (d) OCV, (e) power output, (f) stack efficiency, (g) CPOX outlet temperature, (h) stack outlet temperature, and (i) stack inlet air temperature.

At 760 W power target, stack inlet air temperature constraint was not satisfied °C as an aftereffect of the 23-h self-check. According to Supporting Information Part E Figure S23, the stack inlet air temperature of the model at 760 W was still in the safe region with different trend from the system after 23-h self-check. Regarding the inputs, the increased air mixing valve ratio and positrode air blower workload were restored to their lower bounds. The FU upper bound was active again to maximize the electrical efficiency, while the voltage first dropped to 41.3 V then gradually increased. The lower bound of the stack inlet air temperature was not satisfied at around 26 h due to inconsistency of the dynamic response of the model and plant following the 23-h self-check. Benefiting from the conservative constraints, the stabilized stack inlet air temperature was only 6 °C lower than the lower bound.

At 720 W power target, another 23-h self-check occurred, significantly changing the plant optimum. With the same active constraints on the inputs, the FU upper bound was not active until 36 h. At this moment, the second 23-h self-check led to a different performance curve of the positrode air blower workload. The positrode air pressure reduced significantly without any changes regarding the positrode air blower workload, leading to a much lower flow rate and rapid temperature ramping-up within 1 h from 713.5 to 740 °C. The algorithm recognized this harsh change and immediately reduced the flow rate of fuel to maintain the same amount of power generated with lower energy injection. Hence, the electrical efficiency was maintained at around 32.2%, similar to the 2-input and 3-input cases, while the stack efficiency was increased to 59.1%.

The system dynamic response following the target power increase from 720 to 800 W further evidenced the impact of the performance curve interruption. Unlike the first 800 W power target, the lower bound of the air mixing valve ratio was no longer active. The air mixing valve ratio was increased to maintain the stack outlet temperature at its upper bound, which stabilized at around 801.6 °C at the end of the test. The electrical efficiency and the stack efficiency were higher than those at the first 800 W power target, due to a higher stack inlet air temperature and stack outlet temperature. The same operating point was not reached at the first 800 W power target due to the different positrode air blower operation pattern. This also implied that with the capability of reducing the positrode air blower workload below the PID limit (28%), the system performance could be further improved.

In summary, with 5 inputs controlled, the plant electrical efficiencies at different power targets were increased compared with the 2-input and 3-input cases. The plant optimum was coherent with the model optimum regarding the active constraints at the power targets of 840 W and the second 800 W, while the active constraints of the plant optimum at the power targets of the first 800 W, 760, and 720 W were different from that of the model optimum due to plant response modification that occurred after the 23-h self-check. This event disturbed the positive electrode air injection, which changed the dynamic characteristics of the system. Hence, for slow switching operation, the 5-input strategy is preferred when the 23-h self-check can be turned off, otherwise, 2-input and 3-input strategies should be applied to avoid abrupt operation change.

### CHP Efficiency as Cost Function

#### Two-Input Case

In the 2-input case for CHP efficiency optimization, each power target experienced 40 RTO iterations with a time interval of 3 min, corresponding to around 2 h. An asynchronous control strategy was applied. The results are shown in Figure 7–8.

**Figure 7:**
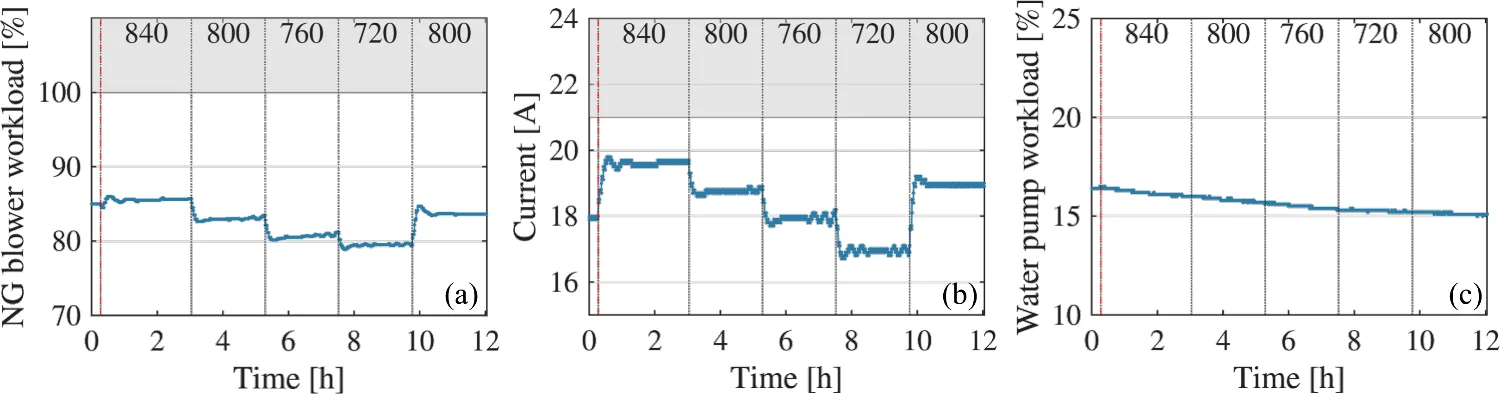
Inputs recording of the 2-input case with CHP efficiency as the cost function, where the number on the top indicates power target and the red dashed line indicates the time of activating CA-RTO algorithm: (a) natural gas blower workload, (b) current, and (c) water pump workload vs time.

**Figure 8:**
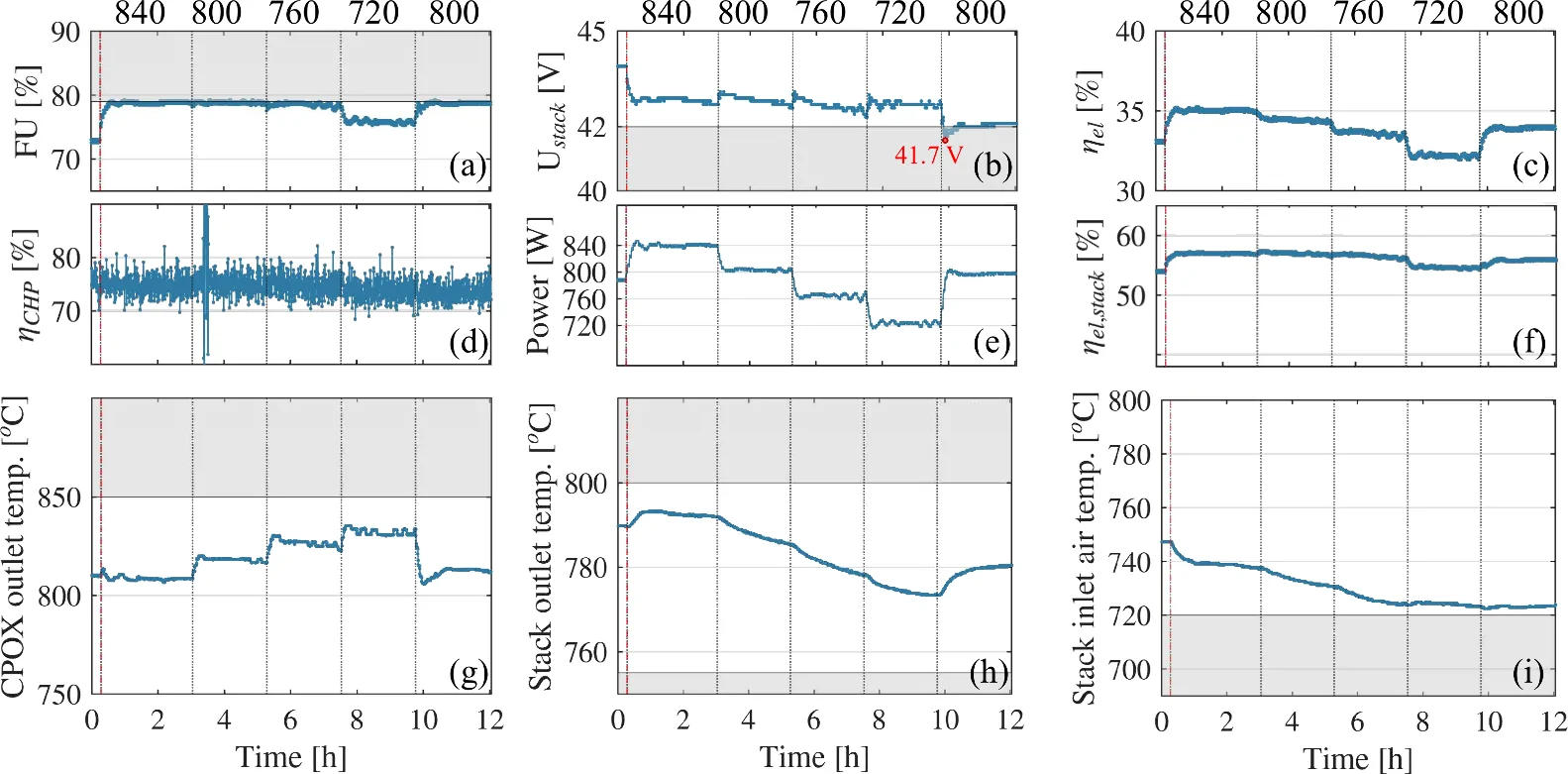
Key indicators and BoP temperature of 2-input case with CHP efficiency as cost function, where the number on the top indicates power target and the red dashed line indicates the time of activating CA-RTO algorithm: (a) FU, (b) stack voltage, (c) electrical efficiency, (d) CHP efficiency, (e) power output, (f) stack efficiency, (g) CPOX outlet temperature, (h) stack outlet temperature, and (i) stack inlet air temperature.

With 2 controlled inputs, the operating points with the optimal CHP efficiency at different power targets were consistent with those of optimal electrical efficiency under the same conditions. The plant optimum showed the same characteristics as the model, except for the active constraint on stack inlet air temperature instead of FU. Due to a more stable and slightly different natural gas composition, the upper bound of the CPOX temperature was no longer active at 720 W, unlike the condition in the 2-input case for optimal electrical efficiency (Figure 4g). At the second 800 W power target, the lower stack outlet temperature and stack inlet air temperature inherited from the previous power target of 720 W led to the activation of the constraints on both the FU and the stack voltage. However, with the stack temperature recovering to the steady state at 800 W, the FU upper bound would still be active, while the voltage lower bound will not be active. Similarly, the voltage was reduced to 41.7 V when switching from 720 to 800 W, due to the mismatch between the ramping rates of current and the NG blower workload.

With a lower natural gas blower workload, the water pump workload decreased continuously during the test to maintain the 10 °C temperature difference between the inlet and outlet water, corresponding to a lower amount of waste heat recovered. Hence, the CHP efficiency stabilized at around 74%. Without manipulating the heat-exchange-loop related inputs (positrode air blower workload and air mixing valve ratio), the plant optimum of CHP efficiency overlapped with the optimal operating points of electrical efficiency in the 2-input case. As the 3-input case showed the same characteristics based on the model optimum results, we directly skipped to the 5-input case.

#### Five-Input Case

Considering the high thermal inertia of the system, an asynchronous control strategy was first tested for CHP efficiency optimization with 360 RTO iterations and a time interval of 3 min for each power target, corresponding to around 18 h. For the synchronous control strategy, each power target only experienced 120 RTO iterations with a time interval of 9 min. Due to the interruption of the 23-h self-check functionality, it was only possible to run the test under the first power target of 840 W. The system showed a high chance of failure with an abrupt positrode air flow rate change.

To optimize the CHP efficiency, it was predicted that the system tended to produce as much heat as possible to increase the outlet water temperature. This led to the following behavior: (1) high fuel injection for thermal energy generation, (2) high air mixing valve ratio to minimize the heat transferred to the air fed to the stack, which could potentially lead to the activation of air temperature lower bound, and (3) a high CPOX air flow rate to compensate for the low stack inlet air temperature and maintain a high stack operating temperature. The impacts of different inputs on the thermal transient response differed, as indicated in Supporting Information Part C Table S2. The CPOX air blower workload and current affected the stack outlet temperature much faster than the positrode air blower workload and the air mixing valve ratio. In this case, when applying the asynchronous strategy at power target of 840 W, the fast change of natural gas blower workload, current and CPOX air blower workload, shown in Figure 9a, increased the stack outlet temperature to 818 °C within 4 h, while the adjustment of the positrode air blower workload and the air mixing valve ratio was so slow that the stack was not cooled down in time. To avoid the emergency shutdown, the test was manually stopped at around 4 h.

**Figure 9:**
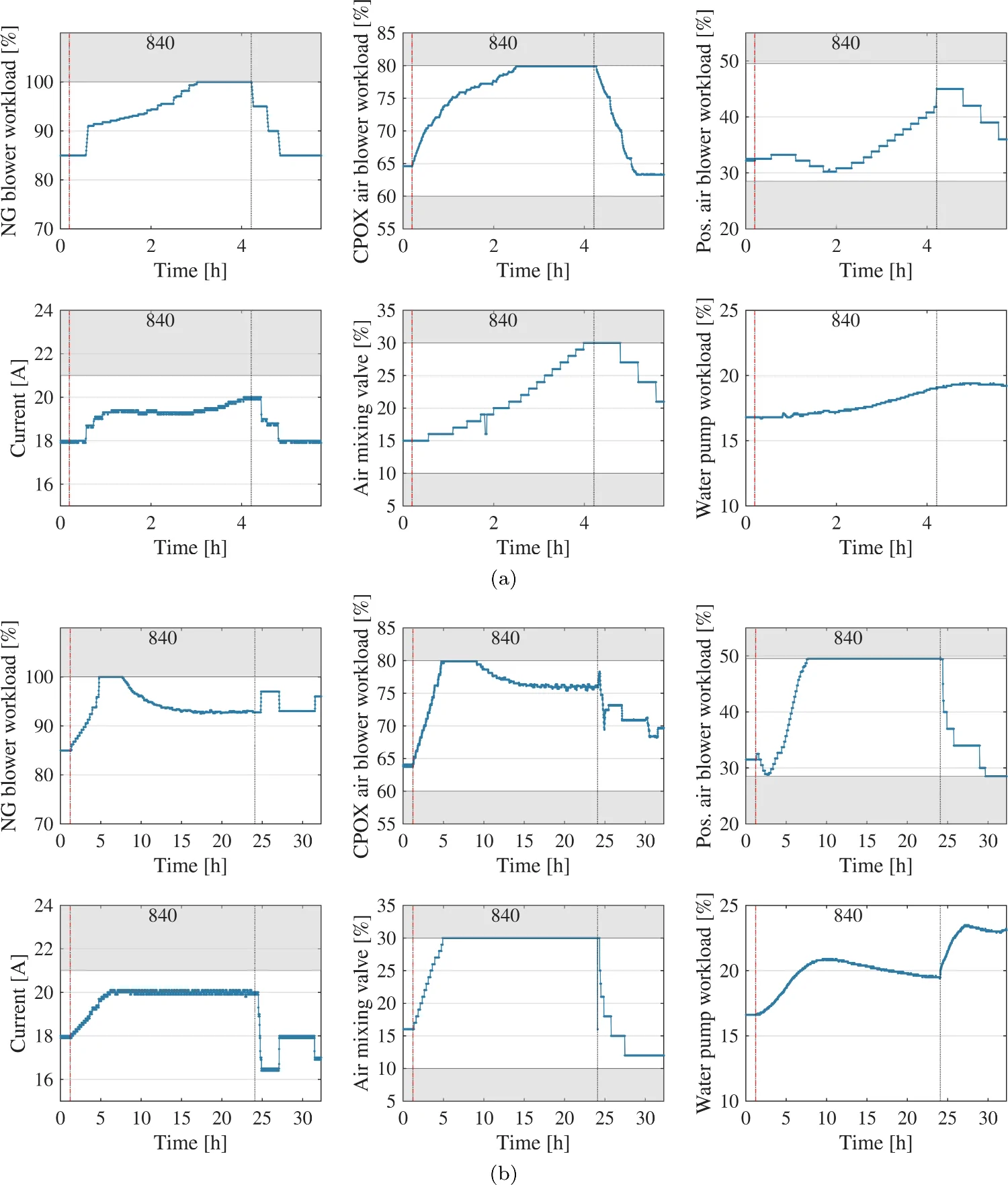
Inputs recording of the 5-input case, (a) asynchronous strategy and (b) synchronous strategy, with CHP efficiency as the cost function, where the number on the top indicates power target and the red dashed line indicates the time of activating CA-RTO algorithm.

As the system did not reach the steady state and the CA-RTO algorithm optimized the system by solving the steady-state problem, the power target was not reached within 4 h via an asynchronous strategy. With the steady-state problem, the power target should be reached after the system stabilized, which was proved by the synchronous control results (Figure 10). The CHP efficiency first decreased and then increased due to the increased natural gas blower workload and the high thermal inertia, while the electrical efficiency (32%) and the stack efficiency (52%) were significantly lower than the cases with optimized electrical efficiency, as is shown in Figure 10.

**Figure 10:**
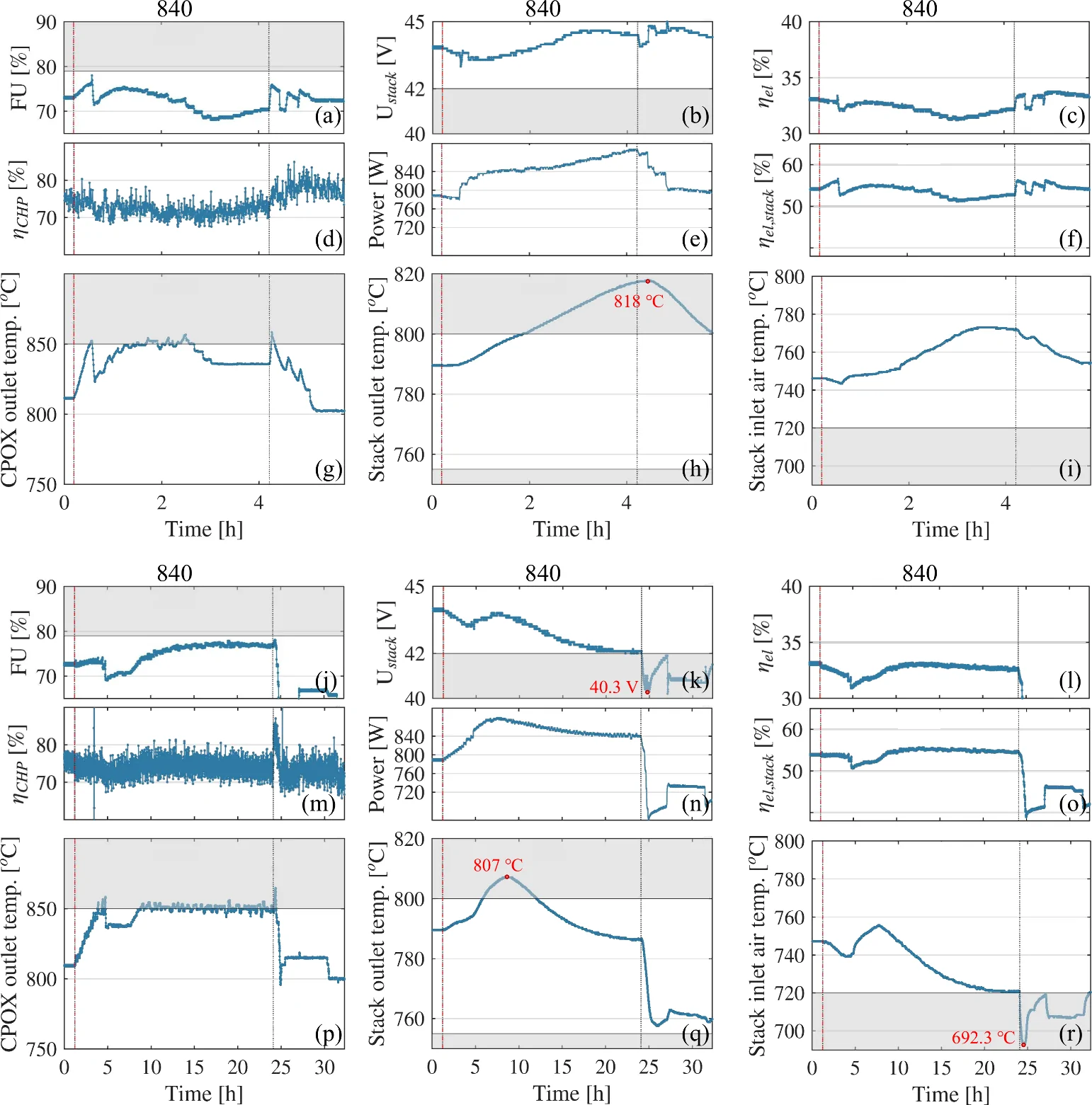
Key indicators and BoP temperature of (a-i) asynchronous control strategy and (j-r) synchronous control strategy with CHP efficiency as cost function, where the number on the top indicates power target and the red dashed line indicates the time of activating CA-RTO algorithm.

At the end of the asynchronous strategy test, the sets of active constraints were coherent with the model behavior, as the upper bound of natural gas blower workload, CPOX air blower workload, and air mixing valve ratio were active and the temperature bound was not active since the steady state was still not reached.

The synchronous control strategy successfully avoided the overheating problem, reached a steady state and proved that the CA-RTO algorithm could reach the optimum with a different set of active constraints. In Figure 9b, the natural gas blower workload and the CPOX air blower workload first reached their upper bound as expected, but then decreased with a transient drop in the CPOX outlet temperature after around 5 h (Figure 10m). Due to the natural gas composition fluctuation, a lower CH_4_ concentration and more impurities with high heat capacities led to a lower CPOX outlet temperature. Nevertheless, two inputs converged to a fixed value at around 15 h. The lower bounds of stack voltage and stack inlet air temperature were respected at around 22 h after a long stabilization duration. This indicated that the continuous evolution of the constraint modifiers, due to high thermal inertia, changed the active constraints to optimize the CHP efficiency and maintain the secure operating condition. The positrode air blower workload and the air mixing valve ratio were kept at their upper bounds during the test to ensure the secure stack inlet and outlet temperatures. Instead of 818 °C stack outlet temperature at around 4 h for the asynchronous control strategy, it reached the peak (807 °C) at around 8 h and stayed within the safe region afterward in the synchronous control strategy case.

In total, six constraints were active, including the power, the positrode air blower workload, the air mixing valve ratio, the CPOX outlet temperature, the stack voltage, and the stack inlet air temperature. This indicated that the plant optimum was reached with 5 constraints active and the optimum was located at the intersection with one additional active constraint. The stack outlet temperature exceeded the upper bound for several hours and then converged to around 786 °C, implying that synchronous strategy was more reliable than asynchronous strategy in this case. The peak of stack outlet temperature was a consequence of (1) an increased fuel injection, (2) more combustion in the CPOX, and (3) an increase of the ohmic heat generation due to a higher current. The stack inlet air temperature was decreased to its lower bound as expected to ensure that a maximum of the exhaust gas heat could be transferred to the cooling water. The CPOX outlet temperature was maintained at its upper bound after around 7 h to increase the stack operating temperature which started to decrease for a higher efficiency. Hence, the upper bounds of the natural gas blower workload and CPOX air blower workload were no longer active to reduce the energy injection with maintained CPOX outlet temperature. The stack voltage slowly reached its lower bound, and the power target was reached after around 15 h.

The CHP efficiency was not significantly improved compared with the initial condition, while more hot water was generated. The CHP efficiency was first increased to around 76% and then stabilized at around 75%, which was similar to that of the initial condition. The electrical efficiency and the stack efficiency were no different from the initial conditions as well. Because of the functionality of the H2O PID, the water pump workload first increased from the initial 16.6% to the peak of 20.0% and then stabilized at around 19.5% to maintain the temperature difference. A higher flow rate led to the decline of heat exchange efficiency and a higher power consumption, which hindered the increment of the CHP efficiency. Hence, the CHP efficiency could be increased by fixing the water pump’s workload.

At around 22 h of the synchronous strategy test, the 23-h self-check was triggered and interrupted the test. The air mixing valve ratio was immediately decreased to 16%. With the positrode air blower workload increased marginally from 49.5% to 49.7%, the performance curve of the positrode air blower was completely changed. The air pressure significantly increased from 12.3 mbar to 16.4 mbar as is indicated in Supporting Information Part E Figure S36. A considerable amount of air passed through the air heat exchanger and entered the stack with small change in the positrode air blower workload. Consequently, the stack inlet air temperature decreased from 720 to 692.3 °C within 1 h and continued to decrease quickly, expected to reach 680 °C soon and trigger the emergency shutdown. The stack outlet temperature decreased from 786.6 to 758.6 °C, and stack voltage was decreased from 42 to 40.3 V, where the emergency shutdown would be triggered at a voltage of 40 V. The sudden disruption could not be handled by the CA-RTO algorithm with a 9 min iteration interval. Hence, all the inputs were manually adjusted to avoid dangerous operating conditions.

In summary, with negatrode-related inputs controlled, the plant optimum of CHP efficiency overlapped with the optimum of the electrical efficiency optimization case. The asynchronous strategy was no longer applicable in the 5-input case because the slow response of the positrode air blower workload and air mixing valve ratio could not cool down the stack in time with a faster increased natural gas blower workload and CPOX air blower workload. The synchronous control strategy successfully pushed the plant to its optimum. The CHP efficiency was not significantly improved, but 20% more hot water was generated instead. The active constraints changed with a slow temperature response, implying that the CA-RTO algorithm can handle high thermal inertia and recognize the correct set of constraints, which are different from the model optimum. The 23-h self-check appeared to impact the system response and should be stopped to allow long-term system optimization.

## Conclusions

This study presented a universal approach for the performance optimization of SOFC systems based on the experimental results from the optimal operation of a micro-CHP SOFC system at secure operating points. The whole test took place in a real-world environment, affected by external natural gas grid composition fluctuations and internal control subsystem interruptions. A considerably simplified zero-dimensional lumped dynamic model amenable to handling significant plant-model mismatch was constructed. A fast constraint-adaptation real-time optimization algorithm was applied to optimize the electrical efficiency and CHP efficiency of the system at different power targets by updating the constraints of the optimization problem with transient system measurements. The proposed approach was able to reach system optimality in the presence of disturbances and high thermal inertia due to the heat exchange loop.

Five system inputs were considered, including the negatrode-related inputs of natural gas blower workload, CPOX air blower workload, and current, and the heat-exchange-loop related inputs of positrode air blower workload and air mixing valve ratio. Different sets of system controlled inputs were tested, including negatrode-related inputs (2-input case and 3-input case) and two-electrode-related inputs (5-input case). The major findings are listed below:

- A simplified dynamic system model based on area-specific resistance equation, effective-number of transfer units method, and continuous stirred-tank reactor model regardless of reaction kinetics was adequate for the description of plant dynamics and the performance optimization, despite plant-model mismatch.
- The CA-RTO algorithm reached the set power targets within 4 iterations during the optimization of electrical efficiency for any number of controlled inputs. The electrical efficiency increased with the number of controlled inputs, where a relative improvement of at least 5% was observed compared to the normal condition. The same number of active constraints as controlled inputs confirmed the adequacy of the CA-RTO algorithm to meet the plant’s optimum.
- The CA-RTO algorithm was robust enough to cope with the composition fluctuation of the natural gas grid, which could cause a 30 °C temperature difference. During the electrical efficiency optimization, the system operation was always maintained in a secure condition when there was no internal programmed interruption.
- When two or three inputs were controlled, optimizing the CHP efficiency led to the same operating conditions as optimizing the electrical efficiency. With five inputs controlled, the CHP efficiency was not improved significantly but the quantity of heated water was increased by 20%.

Overall, the proposed approach can securely drive the system to its optimum in the presence of external disturbance by using only a simplified lumped dynamic model, which reduces the need for experimental parameter estimation. Robustness to external disturbances was demonstrated. This method and workflow can be applied to different SOFC/SOEC systems and other energy systems.

## Supplementary Material





## Notes

Supporting Information: model details (Part A); model parameter estimation (Part B); model validation and settling-time analysis (Part C); model-optimum active constraint sets (Part D); complete plant variable recordings and manufacturer operating limits (Part E); Figures S1–S36; Tables S1–S6 (PDF).
Natural gas blower calibration data: gas-meter readings, measured flow rates at each blower workload set point, and the resulting workload–flow-rate calibration curves (XLSX).

## Nomenclature

**Abbreviation**
API: Application programming interface
ASR: Area specific resistance
AU: Air utilization
BoP: Balance of plant
CA: Constraint-adaptation
CHP: Combined heat and power
COR: Carbon to oxygen ratio
CPOX: Catalytic partial oxidation reactor
CSTR: Continuous stirred-tank reaction
FU: Fuel utilization
GDC: Gadolinia-doped ceria
LSCF: Lanthanum strontium cobaltite ferrite
MA: Modifier adaptation
NLP: Nonlinear programming
OCV: Open circuit voltage
PID: Proportional-integral-derivative
RTO: Real-time optimization
SOFC: Solid oxide fuel cell
SR: Steam reforming
WGS: Water gas shift
YSZ: Yttria-doped zirconia
**Greek symbols**
δ: Time interval between two iterations [1 × 1]
δ_*NLP*_: Time duration to compute the nonlinear programming optimization problem [1 × 1]
**δ**_*input*_: Vector of time duration to change the input variables [*n*_*u*_ × 1]
η_*CHP*_: Combined heat and power efficiency [1 × 1]
η_*el*_: Electrical efficiency [1 × 1]
η_*el*,*stack*_: Stack efficiency [1 × 1]
**ξ**: Steady state functions for the state variables [*n*_*x*_ × 1]
***ε***_*k*_^**H**^: Equality constraint modifiers at iteration k [*n*_*H*_ × 1]
***ε* ^**_*k*_^**H**^: Estimated equality constraint modifiers at iteration k based on the transient measurements [*n*_*H*_ × 1]
***ε***_*k*_^**G**^: Inequality constraint modifiers at iteration k [*n*_*G*_ × 1]
Φ: Model cost function value [1 × 1]
Φ_*p*_: Plant cost function value [1 × 1]
ϕ: Cost function [1 × 1]
**θ**: Vector of adjustable model parameters [*n*_θ_ × 1]
*ΔWL*_*air*,*stack*_: difference of the positrode air blower workload between the optimal value at RTO iteration *k* + 1 and the set point at RTO iteration *k* [1 × 1]
*ΔV*_*air*_: difference of the air mixing valve ratio between the optimal value at RTO iteration *k* + 1 and the set point at RTO iteration *k* [1 × 1]
**Mathematical symbols**
**A**: Active inequality constraints [(*n*_*u*_ – *n*_*H*_) × 1]
*C*: Current [1 × 1]
*C*_*p*,*H*_2_ *O*_: Heat capacity of water [1 × 1]
**f**: Process model equations including mass and energy balances and thermodynamic relationships [*n*_*f*_ × 1]
**F**: Model functions [*n*_*y*_ × 1]
**G**: Inequality constraints values of the model [*n*_*G*_ × 1]
**G** ^*dyn*^: Inequality constraints values of the model during dynamic operation [*n*_*G*_ × 1]
**G**_*p*_^*dyn*^: Inequality constraints values of the plant based on the transient measurements [*n*_*G*_ × 1]
**Ĝ**_*p*_: Estimated inequality constraints values of the plant at steady state [*n*_*G*_ × 1]
**G**_*p*_: Inequality constraints values of the plant [*n*_*G*_ × 1]
**g**_*p*_: Inequality constraints functions of the plant [*n*_*G*_ × 1]
**H**: Equality constraints values of the model [*n*_*H*_ × 1]
**H** ^*dyn*^: Equality constraints values of the model during dynamic operation [*n*_*H*_ × 1]
**H**_*p*_^*dyn*^: Equality constraints values of the plant based on the transient measurements [*n*_*H*_ × 1]
**H**_*p*_: Equality constraints values of the plant [*n*_*H*_ × 1]
**Ĥ**_*p*_: Estimated equality constraints values of the plant at steady state [*n*_*H*_ × 1]
**h**_*p*_: Equality constraints functions of the plant [*n*_*H*_ × 1]
*I*: Identity matrix for filter matrix­[*n*_*u*_ × *n*_*u*_]
**K**: Filter diagonal matrix [*n*_*u*_ × *n*_*u*_]
*P*_*conv*_: Power loss from the converter [1 × 1]
*P*_*el*_: System power output [1 × 1]
*P*_*el*_^*set*^: Power output target set by user [1 × 1]
*P*_*loss*_: Power consumption from the converter, blowers and pump [1 × 1]
*P*_*max*,*CH*_4__: Maximal power of the natural gas blower [1 × 1]
*P*_*max*,*cpox*_: Maximal power of the CPOX air blower [1 × 1]
*P*_*max*,*pos*_: Maximal power of the positrode air blower [1 × 1]
*P*_*max*,*H*_2_ *O*_: Maximal power of the water pump [1 × 1]
*q*_*H*_2_ *O*_: Water flow rate [1 × 1]
*Q*_*O*_2_,*cpox*_: Oxgen flow rate converted rom the CPOX air blower workload [1 × 1]
*Q*_*CH*_4__: Methane flow rate [1 × 1]
*t*: Time [1 × 1]
*t*_*k*_: Time at iteration k [1 × 1]
*T*_*air*_: Mixed inlet air temperature of the positrode [1 × 1]
*T*_*burn*_: Afterburner outlet temperature [1 × 1]
*T*_*cpox*_: Catalytic partial oxidation reactor outlet temperature [1 × 1]
*T*_*H*_2_ *O*,*in*_: Inlet water temperature [1 × 1]
*T*_*H*_2_ *O*,*out*_: Outlet water temperature [1 × 1]
*T*_*stack*_: Stack outlet temperature [1 × 1]
**u**: Input variable vector [*n*_*u*_ × 1]
**u***: Optimal input variable vector [*n*_*u*_ × 1]
**u**_*k*_: Input variable vector at iteration k [*n*_*u*_ × 1]
**u**_*k*+1_^*^: Optimal input variable vector at iteration k+1 [*n*_*u*_ × 1]
**u**_*k*+1_: Input variable vector at iteration k+1 [*n*_*u*_ × 1]
**u** ^*L*^: Vector of inputs lower bounds [*n*_*u*_ × 1]
**u** ^*U*^: Vector of inputs upper bounds [*n*_*u*_ × 1]
*U*_*stack*_: Stack voltage [1 × 1]
*U*_*OCV*_: Open circuit voltage [1 × 1]
*V*_*air*_: Ratio of air bypassing the air heat exchanger [1 × 1]
*WL*_*air*,*cpox*_: Workload of the CPOX air blower [1 × 1]
*WL*_*air*,*stack*_: Workload of the positrode air blower [1 × 1]
*WL*_*CH*_4__: Workload of the natural gas blower [1 × 1]
*WL*_*H*_2_ *O*_: Workload of the water pump [1 × 1]
**x**: State variables [*n*_*x*_ × 1]
**ẋ**: Dynamic state variables [*n*_*x*_ × 1]
**y**_*p*_: Steady-state input-output map of the plant [*n*_*y*_ × 1]
**y**: Output variables predicted by the model [*n*_*y*_ × 1]
