# Supplementary material for: Optimal and Secure Operation of a μ‑CHP Solid Oxide Fuel Cell System
Source: ACS Omega. 2026 Oct 20;11(43):67597–613. doi: 10.1021/acsomega.6c08789 (PMC13738149; doi:10.1021/acsomega.6c08789)
Supplement: Supplementary file 1 [file ao6c08789_si_001.pdf]

Optimal and Secure Operation of a μ-CHP Solid Oxide Fuel Cell System

Hangyu Yu[a,*], Florian Bernard Berset[a], Tafarel de Avila Ferreira[b], Arne Sommerfeld[c] , Gregor Holstermann[c], Cédric Frantz[a], Philippe Aubin[a], Guillaume Jeanmonod[d], Ligang Wang[e,f,], Matthias Boltze[c], Grégory François[b,*] and Jan Van herle[a]

[a] Group of Energy Materials, Swiss Federal Institute of Technology in Lausanne (EPFL), Rue de l'Industrie 17, Sion, 1951, Valais, Switzerland

[b] HES-SO Valais-Wallis, CH-1951, Sion, Switzerland

[c] New Enerday GmbH, Lindenstraβe 45, 17033, Neubrandenburg, Germany

[d] Hydro-Québec (IREQ), Montreal, QC/Canada

[e] National Energy Storage Industry-Education Platform, North China Electric Power University, 102206, Beijing, China

[f] Beijing Laboratory of New Energy Storage Technology, North China Electric Power University, 102206, Beijing, China

[*] Corresponding authors at: Swiss Federal Institute of Technology in Lausanne, Switzerland (Hangyu Yu) and Ecole Spécialisée de Suisse occidentale (Grégory François)

List of Abbreviations

CA	Constraint adaptation
CHP	Combined heat and power
CPOX	Catalytic partial oxidation reactor
CSTR	Continuous stirred-tank reactor
EPFL	Swiss Federal Institute of Technology in Lausanne
ε-NTU	Effectiveness-number of transfer units
HEX	Heat exchanger
PE	Parameter estimation
PCB	Printed circuit board
RTO	Real-time optimization
SOFC	Solid oxide fuel cell
SR	Steam reforming
VD	Validation
WGS	Water gas shift

Nomenclature of the variables used in this deliverable

Variable	Definition
C	Stack current [A]
V_{air}	Air mixing valve ratio [%]
$WL_{air,cpox}$	Workload of the air blower for the CPOX [%]
$WL_{air,stack}$	Workload of the air blower for the stack [%]
WL_{CH_4}	Workload of the natural gas blower [%]
WL_{H_2O}	Workload of the water pump [%]
$F_{air,cpox}$	Exponential factor to convert the CPOX air blower workload into flow rate in NL/min.
$n_{in,cpox}^{i}$	Catalytic partial oxidation reactor inlet gas composition flow rate [mol·s^{-1}]
$n_{out,cpox}^{j}$	Catalytic partial oxidation reactor outlet gas composition flow rate [mol·s^{-1}]
F_{WGS}	Exponential factor to determine the water gas shift reaction in the catalytic partial oxidation reactor.
$C_{p,cpox}$	Heat capacity of the catalytic partial oxidation reactor [J·kg^{-1}·K^{-1}]

m_{cpox}	Mass of the catalytic partial oxidation reactor [kg]
$H_{in,cpox}^{i}(T_{in})$	Inlet gas enthalpy of catalytic partial oxidation reactor [J·mol^{-1}]
$H_{out,cpox}^{j}(T_{cpox})$	Outlet gas enthalpy of catalytic partial oxidation reactor [J·mol^{-1}]
$Q_{loss,cpox}$	Thermal loss of CPOX due to heat interaction with stack and ambient [W]
$F_{conv,stack}^{cpox}$	Coefficient for convection heat transfer between CPOX and stack [W·K]
$F_{conv,amb}^{cpox}$	Coefficient for convection heat transfer between CPOX and ambient [W·K]
$T_{amb,cpox}$	Ambient temperature of the CPOX [K]
T_{in}	Inlet fuel temperature fixed at 623 [K]
T_{cpox}	CPOX outlet temperature [K]
$n_{react}^{H_2}$	Reacted hydrogen mole flow rate [mol·s^{-1}]
N_{cell}	Number of cells
F	Faraday constant [C·mol^{-1}]
$n_{in,pos}^{air}$	Air flow rate of the positrode [mol·s^{-1}]
$F_{air,stack,pre}$	Pre-exponential factor to compute the air flow rate, initialized at 342 [NL·min^{-1}]
$F_{air,stack,exp}$	Exponential factor for air flow rate conversion, initialized at 0.6
$n_{in,pos}^{O_2}$	Oxygen flow rate of the positrode [mol·s^{-1}]
$n_{in,pos}^{N_2}$	Nitrogen flow rate of the positrode [mol·s^{-1}]
m_{stack}	Stack mass [kg]
$C_{p,stack}$	Heat capacity of the stack [J·kg^{-1}·K^{-1}]
$n_{in,neg}^{i}$	SOFC stack negatrode inlet gas composition flow rate [mol·s^{-1}]
$H_{in,neg}^{i}(T_{cpox})$	Enthalpy of gas from CPOX [J·mol^{-1}]
$H_{in,pos}^{O_2}(T_{air})$	Enthalpy of O_2 in the positrode [J·mol^{-1}]
$H_{in,pos}^{N_2}(T_{air})$	Enthalpy of N_2 in the positrode [J·mol^{-1}]
$n_{out,stack}^{j}$	SOFC stack outlet gas composition flow rate [mol·s^{-1}]
$H_{out,stack}^{j}(T_{stack})$	Outlet gas enthalpy of the stack [J·mol^{-1}]
$Q_{loss,stack}$	Thermal loss of stack [W]
$F_{conv,cpox}^{stack}$	Coefficient for convection heat transfer between stack and CPOX [W·K^{-1}]
$F_{conv,pcb}^{stack}$	Coefficient for convection heat transfer between stack and ambient [W·K^{-1}]
$F_{conv,burn}^{stack}$	Coefficient for convection heat transfer between stack and afterburner [W·K^{-1}]
T_{air}	Positrode inlet air temperature [K]
T_{hexc}	Outlet temperature of the cold fluid of the air heat exchanger [K]
T_{burn}	Outlet temperature of the afterburner [K]
T_{stburn}	Temperature of the startup burner [K]
$Q_{conv,burn}^{air}$	Heat transfer between the afterburner and the mixed air [W]
$F_{conv,burn}^{air}$	Coefficient for convection heat transfer between air and afterburner [W·K^{-1}]
T_{pcb}	Ambient temperature for the stack [K]
$C_{p,air}$	Heat capacity of the air [J·kg^{-1}·K^{-1}]

U_{stack}	Stack voltage [V]
U_{nernst}	Nernst voltage [V]
A_{active}	Cell electroactive area [cm^2]
A_0	Fitted parameter of ASR model [$Ohm\cdot m^2$]
E_A	Fitted parameter of ASR model [J/mol]
R	Ideal gas constant [$J\cdot K^{-1}\cdot mol^{-1}$]
U_s	Standard redox potential [V]
dU_1	Voltage drop coefficient due to water temperature increase in liquid phase [V]
dU_2	Voltage drop coefficient due to steam temperature increase [V]
$x_{out,neg}^{H_2}$	Hydrogen molar fraction in the outlet gas of negatrode
$x_{out,pos}^{O_2}$	Oxygen molar fraction in the outlet gas of positrode
$x_{out,neg}^{H_2O}$	Steam molar fraction in the outlet gas of negatrode
k_{nernst}	Correction term in the Nernst equation
FU	Fuel utilization ratio
AU	Air utilization ratio
m_{burn}	Afterburner mass [kg]
$C_{p,burn}$	Heat capacity of the afterburner [$J\cdot kg^{-1}\cdot K^{-1}$]
$n_{in,burn}^{i}$	Mole flow rates of the inlet gas for the afterburner [$mol\cdot s^{-1}$]
$H_{in,burn}^{i}(T_{stack})$	Inlet gas enthalpy of the afterburner [$J\cdot mol^{-1}$]
$n_{out,burn}^{j}$	Mole flow rates of the inlet gas for the afterburner [$mol\cdot s^{-1}$]
$H_{out,burn}^{j}(T_{burn})$	Outlet gas enthalpy of the afterburner [$J\cdot mol^{-1}$]
$Q_{loss,burn}$	Thermal loss of afterburner [W]
$F_{conv,stack}^{burn}$	Coefficient for convection heat transfer between afterburner and stack [$W\cdot K^{-1}$]
$F_{conv,stburn}^{burn}$	Coefficient for convection heat transfer between afterburner and startup burner [$W\cdot K^{-1}$]
m_{hexh}	Mass of the air heat exchanger at the hot side [kg]
m_{hexc}	Mass of the air heat exchanger at the cold side [kg]
$C_{p,hexh}$	Heat capacity of the air heat exchanger's hot side [$J\cdot kg^{-1}\cdot K^{-1}$]
$C_{p,hexc}$	Heat capacity of the air heat exchanger's cold side [$J\cdot kg^{-1}\cdot K^{-1}$]
$n_{in,hexh}^{i}$	Mole flow rates of the inlet gas at the hot side of air heat exchanger [$mol\cdot s^{-1}$]
$H_{in,hexh}^{i}(T_{burn})$	Inlet gas enthalpy at the hot side of air heat exchanger [$J\cdot mol^{-1}$]
$n_{out,hexh}^{j}$	Mole flow rates of the inlet gas at the hot side of air heat exchanger [$mol\cdot s^{-1}$]
$H_{out,hexh}^{j}(T_{hexh})$	Outlet gas enthalpy at the hot side of air heat exchanger [$J\cdot mol^{-1}$]
$n_{in,hexc}^{i}$	Mole flow rates of the inlet gas at the cold side of air heat exchanger [$mol\cdot s^{-1}$]
$H_{in,hexc}^{i}(T_{in})$	Inlet gas enthalpy at the cold side of air heat exchanger [$J\cdot mol^{-1}$]
$n_{out,hexc}^{j}$	Mole flow rates of the outlet gas at the cold side of air heat exchanger [$mol\cdot s^{-1}$]
$H_{out,hexc}^{j}(T_{hexc})$	Outlet gas enthalpy at the cold side of air heat exchanger [$J\cdot mol^{-1}$]
$Q_{NTU,air}$	Heat transfer between the hot side and the cold side [W]

$C_{min,air}$	Lower value between the heat capacities of cold flow and hot flow in the air heat exchanger [J·mol^{-1}]
$\epsilon_{hex,air}$	Ratio between the actual heat transfer rate and the maximum possible heat transfer rate in the air heat exchanger
$C_{p,hexh,gas}$	Gas heat capacity in the hot side of the air heat exchanger [J·mol^{-1}]
$x_{in,hexh}^{i}$	Mole fraction of the individual gas in the hot side of the air heat exchanger
$c_{p,hexh}^{i}(T_{hexh})$	Heat capacity of the individual gas in the hot side of the air heat exchanger [J·mol^{-1}]
$C_{p,hexc,gas}$	Gas heat capacity in the cold side of the air heat exchanger [J·mol^{-1}]
$x_{in,hexc}^{i}$	Mole fraction of the individual gas in the cold side of the air heat exchanger
$c_{p,hexc}^{i}(T_{hexc})$	heat capacity of the individual gas in the cold side of the air heat exchanger [J·mol^{-1}]
m_{whexh}	Mass of the water heat exchanger at the hot side [kg]
m_{whexc}	Mass of the water heat exchanger at the cold side [kg]
$C_{p,whexh}$	Heat capacity of the water heat exchanger's hot side [J·kg^{-1}·K^{-1}]
$C_{p,whexc}$	Heat capacity of the water heat exchanger's cold side [J·kg^{-1}·K^{-1}]
$m_{est}C_{p,est}$	Additional parameter to be estimated for the dynamic behavior of the air HEX [J·K^{-1}]
$n_{in,whexh}^{i}$	Mole flow rates of the inlet gas at the hot side of air heat exchanger [mol·s^{-1}]
$H_{in,whexh}^{i}(T_{hexh})$	Inlet gas enthalpy at the hot side of water heat exchanger [J·mol^{-1}]
$n_{out,whexh}^{j}$	Mole flow rates of the outlet gas at the hot side of water heat exchanger [mol·s^{-1}]
$H_{out,whexh}^{j}(T_{whexh})$	Outlet gas enthalpy at the hot side of water heat exchanger [J·mol^{-1}]
$n_{in,whexc}^{H_2O}$	Inlet water mole flow rate at the cold side of water heat exchanger [mol·s^{-1}]
$H_{in,whexc}^{H_2O}(T_{in,H_2O})$	Inlet water enthalpy at the cold side of water heat exchanger [J·mol^{-1}]
$n_{out,whexc}^{H_2O}$	Outlet water mole flow rate at the cold side of water heat exchanger [mol·s^{-1}]
$H_{out,whexc}^{H_2O}$	Outlet water enthalpy at the cold side of water heat exchanger [J·mol^{-1}]
Q_{NTU,H_2O}	Heat transfer between the hot side and the cold side [W]
C_{min,H_2O}	Lower value between the heat capacities of cold flow and hot flow in the water heat exchanger [J·mol^{-1}]
ϵ_{hex,H_2O}	Ratio between the actual heat transfer rate and the maximum possible heat transfer rate in the water heat exchanger
$C_{p,whexh,gas}$	Gas heat capacity in the hot side of the water heat exchanger [J·mol^{-1}]
$x_{in,whexh}^{i}$	Mole fraction of the individual gas in the hot side of water heat exchanger
$c_{p,whexh}^{i}(T_{whexh})$	Heat capacity of the individual gas in the hot side of water heat exchanger
$C_{p,hexc,H_2O}(T_{out,H_2O})$	Water heat capacity in the cold side of water heat exchanger [J·mol^{-1}]
SQ	Sum of squares of the deviations of the model output and the plant measurement
$k_{i,pe}$	Scaling factor to keep the deviations of all the outputs at the same magnitude
$\boldsymbol{y}_{i}^{j}$	Plant measurement
Y_{i}^{j}	Model output
$\boldsymbol{p}_{i}$	Vector of the parameters to be estimated

$\boldsymbol{p}_i^L$	Lower bound vector of the parameters
$\boldsymbol{p}_i^U$	Upper bound vector of the parameters
$T_{p,cpox}^j$	Plant measurement of CPOX outlet temperature [K]
$T_{m,cpox}^j$	Model output of CPOX outlet temperature [K]
$WL_{CH_4}^j$	Natural gas blower workload at time j [%]
$WL_{air,cpox}^j$	Catalytic partial oxidation reactor air blower workload at time j [%]
$T_{p,stack}^j$	Plant measurement of stack outlet temperature at time j [K]
$\boldsymbol{u}_{cpox}$	Input vector of the CPOX model parameter estimation
$\boldsymbol{p}_{cpox}$	Vector of the parameters estimated in CPOX model
$\boldsymbol{p}_{cpox}^L$	Lower bound vector of the parameters in CPOX model
$\boldsymbol{p}_{cpox}^U$	Upper bound vector of the parameters in CPOX model
U_p^j	Voltage measurement [V]
U_m^j	Model voltage output [V]
$\boldsymbol{u}_{vol}$	Input vector of the stack model voltage parameter estimation
$\boldsymbol{p}_{vol}$	Vector of the parameters estimated for stack voltage
$\boldsymbol{p}_{vol}^L$	Lower bound vector of the parameters for stack voltage
$\boldsymbol{p}_{vol}^U$	Upper bound vector of the parameters for stack voltage
$T_{m,stack}^j$	Stack model temperature output [K]
$\boldsymbol{u}_{stack}$	Input vector of the parameter estimation for stack outlet temperature
$\boldsymbol{p}_{stack}$	Vector of the parameters estimated for the stack temperature
$\boldsymbol{p}_{stack}^L$	Lower bound vector of the parameters for the stack temperature
$\boldsymbol{p}_{stack}^U$	Upper bound vector of the parameters for the stack temperature
$T_{p,pcb}^j$	Plant measurement of the PCB temperature [K]
$T_{p,burn}^j$	Plant measurement of the afterburner outlet temperature [K]
$T_{p,air}^j$	Plant measurement of the positrode inlet air temperature [K]
$T_{m,burn}^j$	Model output of afterburner outlet temperature [K]
$\boldsymbol{u}_{burn}$	Input vector of the afterburner model parameter estimation
$\boldsymbol{p}_{burn}$	Vector of the parameters estimated for the afterburner temperature
$\boldsymbol{p}_{burn}^L$	Lower bound vector of the parameters for the afterburner temperature
$\boldsymbol{p}_{burn}^U$	Upper bound vector of the parameters for the afterburner temperature
$T_{m,stburn}^j$	Model output of startup burner outlet temperature [K]
$\boldsymbol{u}_{stburn}$	Input vector of the startup burner model parameter estimation
$\boldsymbol{p}_{stburn}$	Vector of the parameters estimated for the startup burner temperature
$\boldsymbol{p}_{stburn}^L$	Lower bound vector of the parameters for the startup burner temperature
$\boldsymbol{p}_{stburn}^U$	Upper bound vector of the parameters for the startup burner temperature
$T_{m,air}^j$	Model temperature output of the mixed air [K]
$\boldsymbol{u}_{hex}$	Input vector of the startup burner model parameter estimation

$\boldsymbol{p}_{hex}$	Vector of the parameters estimated for the air HEX outlet temperature
$\boldsymbol{p}_{hex}^{L}$	Lower bound vector of the parameters for the air HEX outlet temperature
$\boldsymbol{p}_{hex}^{U}$	Upper bound vector of the parameters for the air HEX outlet temperature
T_{p,out,H_2O}^{j}	Plant temperature of the heated water [K]
T_{m,out,H_2O}^{j}	Model temperature output of the heated water [K]
$\boldsymbol{u}_{whex}$	Input vector of the water HEX model parameter estimation
$\boldsymbol{p}_{whex}$	Vector of the parameters estimated for the water HEX outlet temperature
$\boldsymbol{p}_{whex}^{L}$	Lower bound vector of the parameters for the water HEX outlet temperature
$\boldsymbol{p}_{whex}^{U}$	Upper bound vector of the parameters for the water HEX outlet temperature
$\boldsymbol{u}_{sys}$	Input vector of the system model parameter estimation
$\boldsymbol{p}_{sys}$	Vector of the parameters estimated for the whole system
$\boldsymbol{p}_{sys}^{L}$	Lower bound vector of the parameters for the whole system
$\boldsymbol{p}_{sys}^{U}$	Upper bound vector of the parameters for the whole system
LHV_{CH_4}	Lower heating value of methane [mol·J]
q_{H_2O}	Water flow rate [mol·s^{-1}]
C_{p,H_2O}	Water heat capacity [J·mol^{-1}]
P_{el}	Model power output [W]
P_{el}^{set}	Power output target [W]
P_{conv}	Power loss from the converter [W]
P_{max,CH_4}	Maximal power of the natural gas blower [W]
$P_{max,cpox}$	Maximal power of the CPOX air blower [W]
$P_{max,pos}$	Maximal power of the positrode air blower [W]
$P_{max,H_2\text{O}}$	Maximal power of the water pump [W]
$\hat{\epsilon}_{k}^{P_{el}}$	Modifiers of the power [W]
$\hat{\epsilon}_{k}^{T_{cpox}}$	Modifiers of the CPOX outlet temperature [K]
$\hat{\epsilon}_{k}^{T_{stack}}$	Modifiers of the stack outlet temperature [K]
$\hat{\epsilon}_{k}^{U_{stack}}$	Modifiers of the stack voltage[V]
$\hat{\epsilon}_{k}^{T_{burn}}$	Modifiers of the afterburner outlet temperature [K]
$\hat{\epsilon}_{k}^{T_{air}}$	Modifiers of the air inlet temperature [K]

Table of Contents

LIST OF ABBREVIATIONS **1**

NOMENCLATURE OF THE VARIABLES USED IN THIS DELIVERABLE **1**

PART A. SYSTEM MODEL FORMULATION **8**

1. CATALYTIC PARTIAL OXIDATION REACTOR MODEL 8
2. SOLID OXIDE FUEL CELL STACK MODELLING 9
3. AFTERBURNER MODELLING 13
4. STARTUP BURNER MODELLING 14
5. AIR HEAT EXCHANGER MODELLING 14
6. WATER HEAT EXCHANGER MODELLING 15

PART B. PARAMETER ESTIMATION FOR SIMPLIFIED DYNAMIC SYSTEM MODEL **16**

1. CPOX MODEL 17
2. SOLID OXIDE FUEL CELL STACK MODEL 17
3. AFTERBURNER MODEL 18
4. STARTUP BURNER MODEL 18
5. AIR HEAT EXCHANGER MODEL 19
6. WATER HEAT EXCHANGER MODEL 19
7. INTEGRATED SYSTEM MODEL 20

PART C. SYSTEM TEST MATRIX AND MODEL PARAMETER ESTIMATION **20**

PART D. MODEL OPTIMUM **25**

PART E. RESULTS OF CA RTO ALGORITHM **27**

1. ELECTRICAL EFFICIENCY AS THE COST FUNCTION 28

1.1. 2 INPUTS CASE 28

1.2. 3 INPUTS CASE 30

1.3. 5 INPUTS CASE 32

1.4. 5-INPUT CASE RESULTS WITH CPOX AIR BLOWER LOWER BOUND AT 50% 34

1.5. EVIDENCE OF PLANT-MODEL MISMATCH AND IMPACT FROM 23-HOUR SELF-CHECK 36

2. CHP EFFICIENCY AS THE COST FUNCTION 37

2.1. 2 INPUTS CASE 37

2.2. 5 INPUTS CASE WITH ASYNCHRONOUS CONTROL STRATEGY 39

2.3. 5 INPUTS CASE WITH SYNCHRONOUS CONTROL STRATEGY 41

Part A. System model formulation

1. Catalytic partial oxidation reactor model

Unlike traditional methane steam reforming reaction, partial oxidation of methane comprises three reactions: (1) combustion of methane, (2) steam reforming of methane with carbon monoxide and hydrogen as the product, (3) dry reforming of methane with hydrogen and carbon monoxide as products. The chemical reaction formulas are listed below:

$$\begin{aligned}
&\text{Reaction 1:} \quad CH_4 + 2O_2 \Rightarrow CO_2 + 2H_2O \quad (\Delta H_{(298K)} = -802kJ/mol) \\
&\text{Reaction 2:} \quad CH_4 + H_2O \Leftrightarrow CO + 3H_2 \quad (\Delta H_{(298K)} = 206kJ/mol) \\
&\text{Reaction 3:} \quad H_2O + CO \Leftrightarrow CO_2 + H_2 \quad (\Delta H_{(298K)} = -41.2kJ/mol)
\end{aligned} \tag{S1}$$

It is assumed that CPOX is one single thermal body with a single temperature as an ideal continuous stirred-tank reactor (CSTR), meaning that the composition and temperature are uniform everywhere in CPOX and the outlet stream composition and physical properties are the same as those inside the reactor tank.

Mass balance. The flow rates of all components that enter CPOX are considered, namely, methane, oxygen, and nitrogen. Based on the CPOX test results from the manufacturer, New Enerday GmbH, the outlet gas is mainly composed of hydrogen and carbon monoxide, and there is no leftover oxygen. In this case, several assumptions have been made to simply the mass balance of the CPOX model:

- The mass balance is at steady state at the transition of different operating conditions. This means that the quantity of the chemicals varies almost instantaneously, and the overall dynamics are dominated by the energy balance and the corresponding variations in temperature.
- The conversion of CH_4 and O_2 into CO and H_2 is assumed to be complete. When there is surplus O_2, H_2 combustion and water gas shift reaction are considered by using simplified Arrhenius equation instead of detailed reaction kinetic models.

Based on the assumptions, the mass balance is expressed by considering two cases – (1) surplus O_2, and (2) insufficient O_2, where the CPOX inlet can be expressed as below:

$$\begin{aligned}
n_{in,cpox}^{CH_4} &= \frac{4.1224 \times WL_{CH_4} + 0.4894}{22.4 \times 60} \\
n_{in,cpox}^{O_2} &= \frac{20 \times WL_{air,cpox}{}^{F_{air,cpox}} \times 21\%}{22.4 \times 60} \\
n_{in,cpox}^{N_2} &= \frac{20 \times WL_{air,cpox}{}^{F_{air,cpox}} \times 79\%}{22.4 \times 60}
\end{aligned} \tag{S2}$$

where the conversion from natural gas blower workload to natural gas flow rate is calculated via the in-house natural gas flowmeter as is shown in the supplementary information (excel sheet), $F_{air,cpox}$ is the exponential factor to convert the CPOX air blower workload into flow rate in NL/min. When the amount of O_2 is not enough to convert CH_4 into CO and H_2, there will be CH_4 left and the mass balance can be expressed as below:

$$\begin{pmatrix} n_{out,cpox}^{H_2O} \\ n_{out,cpox}^{CH_4} \\ n_{out,cpox}^{CO} \\ n_{out,cpox}^{H_2} \\ n_{out,cpox}^{CO_2} \\ n_{out,cpox}^{O_2} \\ n_{out,cpox}^{N_2} \end{pmatrix} = \begin{pmatrix} 0 & 0 & 0 \\ 1 & -2 & 0 \\ 0 & 2 & 0 \\ 0 & 4 & 0 \\ 0 & 0 & 0 \\ 0 & 0 & 0 \\ 0 & 0 & 1 \end{pmatrix} \begin{pmatrix} n_{in,cpox}^{CH_4} \\ n_{in,cpox}^{O_2} \\ n_{in,cpox}^{N_2} \end{pmatrix} \tag{S3}$$

When the amount of O_2 is enough to convert CH_4 into CO and H_2, the mass balance can be expressed as below:

$$\begin{pmatrix} \dfrac{n_{out,cpox}^{H_2O}}{1-\exp(-\frac{F_{WGS}}{T_{cpox}})} \\ n_{out,cpox}^{CH_4} \\ n_{out,cpox}^{CO} \\ n_{out,cpox}^{H_2} \\ \dfrac{n_{out,cpox}^{CO_2}}{\exp(-\frac{F_{WGS}}{T_{cpox}})} \\ n_{out,cpox}^{O_2} \\ n_{out,cpox}^{N_2} \end{pmatrix} = \begin{pmatrix} -1 & 2 & 0 \\ 0 & 0 & 0 \\ 1+\exp(-\frac{F_{WGS}}{T_{cpox}}) & -2\times\exp(-\frac{F_{WGS}}{T_{cpox}}) & 0 \\ 3-\exp(-\frac{F_{WGS}}{T_{cpox}}) & -2+2\times\exp(-\frac{F_{WGS}}{T_{cpox}}) & 0 \\ -1 & 2 & 0 \\ 0 & 0 & 0 \\ 0 & 0 & 1 \end{pmatrix} \begin{pmatrix} n_{in,cpox}^{CH_4} \\ n_{in,cpox}^{O_2} \\ n_{in,cpox}^{N_2} \end{pmatrix} \quad \text{(S4)}$$

Where F_{WGS} is the exponential factor to determine the portion of WGS reaction, T_{cpox} is the CPOX outlet temperature.

Energy balance. Since CPOX is assumed to be a continuous stirred-tank reactor, the outlet temperature equals the temperature inside the reactor. According to how the CPOX is positioned inside the system's box, the convection heat transfer from the stack and the convection heat transfer from the ambient are considered as heat loss. The energy balance can be written as the summation of the energy flowing and out from the CPOX via the streams as well as the heat loss due to the convection heat transfer with stack and ambient. The energy balance is expressed as below:

$$\begin{gathered} m_{cpox}C_{p,cpox}\frac{dT_{cpox}}{dt} = \sum_i n_{in,cpox}^{i}H_{in,cpox}^{i}(T_{in}) - \sum_j n_{out,cpox}^{j}H_{out,cpox}^{j}(T_{cpox}) - Q_{loss,cpox} \\ Q_{loss,cpox} = F_{conv,stack}^{cpox}\times(T_{cpox}-T_{stack}) + F_{conv,amb}^{cpox}\times(T_{cpox}-T_{amb,cpox}) \end{gathered} \quad \text{(S5)}$$

where m_{cpox} is the mass of the CPOX, $C_{p,cpox}$ is the heat capacity of the CPOX, $H_{in,cpox}^{i}(T_{in})$ is the inlet gas enthalpy of CPOX, T_{in} is the inlet fuel temperature fixed at 623 K, $H_{out,cpox}^{j}(T_{cpox})$ is the outlet gas enthalpy of CPOX, $n_{in,cpox}^{i}$ is the mole flow rates of the CPOX inlet gas, $n_{out,cpox}^{j}$ is the mole flow rates of the CPOX outlet gas, i denotes the inlet gas species of CPOX, j denotes the outlet gas species of CPOX, $Q_{loss,cpox}$ is the heat loss due to the convection heat transfer with SOFC and ambient, $F_{conv,stack}^{cpox}$ is the coefficient for convection heat transfer between CPOX and stack, $F_{conv,amb}^{cpox}$ is the coefficient for convection heat transfer between CPOX and ambient, $T_{amb,pcb}$ is the ambient temperature regarding the CPOX.

2. Solid oxide fuel cell stack modelling

SOFC modelling focuses on three aspects: (1) mass balance, (2) energy balance, and (3) stack voltage. Similar to CPOX, the SOFC stack is assumed to be one single thermal body with a single temperature, i.e., an ideal CSTR. In this case, the stack is considered to have uniform composition and temperature everywhere, and the downstream of the stack has the same gas composition as the stack. The main reactions considered in the stack are listed below:

$$\begin{gathered} H_2 + \frac{1}{2}O_2 \Rightarrow H_2O \\ CO + H_2O \Rightarrow CO_2 + H_2 \\ CH_4 + 2H_2O \Rightarrow CO_2 + 4H_2 \end{gathered} \quad \text{(S6)}$$

Mass balance considers the flow rates of all components that enter and leave the stack. The inlet gas flow rate at the negatrode side equals the outlet gas flow rate of CPOX, while the inlet gas flow rates at the positrode side and the reacted hydrogen flow rate are expressed below:

$$
\begin{aligned}
n_{react}^{H_2} &= \frac{C \times N_{cell}}{2F} \\
n_{in,pos}^{air} &= \frac{F_{air,stack,pre} \times {WL_{air,stack}}^{F_{air,stack,exp}}}{22.4 \times 60} \\
n_{in,pos}^{O_2} &= n_{in,pos}^{air} \times 21\% \\
n_{in,pos}^{N_2} &= n_{in,pos}^{air} \times 79\%
\end{aligned}
\tag{S7}
$$

where $n_{react}^{H_2}$ is the reacted hydrogen, N_{cell} is the number of the cells, F is the Faraday constant, $n_{in,pos}^{air}$ is the positrode air flow rate, $F_{air,stack,pre}$ is the pre-exponential factor to compute the air flow rate, initialized at 342, $F_{air,stack,exp}$ is the exponential factor for air flow rate conversion, initialized at 0.6 , $n_{in,pos}^{O_2}$ is the positrode O_2 mole flow rate, $n_{in,pos}^{N_2}$ is the positrode N_2 mole flow rate.

Due to the complex outlet gas composition of CPOX and the fast response of electrochemical reaction, several assumptions are made as below.

- The mass balance is at steady state at the transition of different operating conditions, since the dynamics of the electrochemical reaction are very fast, i.e., it responds within one second when the operating condition changes. This means that the quantity of the chemicals varies almost instantaneously, and the overall dynamics are dominated by the energy balance and the corresponding variations of temperature.
- CH_4 and CO are converted into H_2 and CO_2 via steam reforming (SR) and water gas shift (WGS) reactions before the stack. Depending on the current and the inlet steam flow rate, three cases are introduced:
  (1) The summation of the generated steam flow rate due to electrochemical reaction and the negatrode inlet steam flow rate is higher than the steam flow rate required for both SR and WGS reactions, i.e., $n_{react}^{H_2} + n_{in,neg}^{H_2O} > n_{in,neg}^{CO} + 2n_{in,neg}^{CH_4}$. This means that all the CO and CH_4 has been converted into H_2 and CO_2. In this case, the mass balance can be expressed as below:

$$
\begin{pmatrix} n_{out,neg}^{H_2O} \\ n_{out,neg}^{CH_4} \\ n_{out,neg}^{CO} \\ n_{out,neg}^{H_2} \\ n_{out,neg}^{CO_2} \\ n_{out,neg}^{O_2} \\ n_{out,neg}^{N_2} \\ n_{out,pos}^{O_2} \\ n_{out,pos}^{N_2} \end{pmatrix}
=
\begin{pmatrix}
1 & 1 & -2 & -1 & 0 & 0 & 0 & 0 & 0 & 0 \\
0 & 0 & 0 & 0 & 0 & 0 & 0 & 0 & 0 & 0 \\
0 & 0 & 0 & 0 & 0 & 0 & 0 & 0 & 0 & 0 \\
-1 & 0 & 4 & 1 & 1 & 0 & 0 & 0 & 0 & 0 \\
0 & 0 & 1 & 1 & 0 & 1 & 0 & 0 & 0 & 0 \\
0 & 0 & 0 & 0 & 0 & 0 & 1 & 0 & 0 & 0 \\
0 & 0 & 0 & 0 & 0 & 0 & 0 & 1 & 0 & 0 \\
-0.5 & 0 & 0 & 0 & 0 & 0 & 0 & 0 & 1 & 0 \\
0 & 0 & 0 & 0 & 0 & 0 & 0 & 0 & 0 & 1
\end{pmatrix}
\begin{pmatrix} n_{react}^{H_2} \\ n_{in,neg}^{H_2O} \\ n_{in,neg}^{CH_4} \\ n_{in,neg}^{CO} \\ n_{in,neg}^{H_2} \\ n_{in,neg}^{CO_2} \\ n_{in,neg}^{O_2} \\ n_{in,neg}^{N_2} \\ n_{in,pos}^{O_2} \\ n_{in,pos}^{N_2} \end{pmatrix}
\tag{S8}
$$

  (2) The summation of the generated steam flow rate due to electrochemical reaction and the negatrode inlet steam flow rate is higher than the steam flow rate required for WGS reaction, but lower than

the steam flow rate required for both WGS reaction and SR reaction, i.e., $n_{react}^{H_2} + n_{in,neg}^{H_2O} > n_{in,neg}^{CO}$ and $n_{react}^{H_2} + n_{in,neg}^{H_2O} - n_{in,neg}^{CO} < 2n_{in,neg}^{CH_4}$. This means that all the CO has been converted into H_2 and CO_2, but only a small part of CH_4 is converted into H_2 and CO_2. In this case, the steam at the outlet will be exhausted, while its flow rate is set at 10^{-6} mol/s to be applied in the Nernst equation:

$$\begin{pmatrix} n_{out,neg}^{H_2O} \\ n_{out,neg}^{CH_4} \\ n_{out,neg}^{CO} \\ n_{out,neg}^{H_2} \\ n_{out,neg}^{CO_2} \\ n_{out,neg}^{O_2} \\ n_{out,neg}^{N_2} \\ n_{out,pos}^{O_2} \\ n_{out,pos}^{N_2} \end{pmatrix} = \begin{pmatrix} 0 & 0 & 0 & 0 & 0 & 0 & 0 & 0 & 0 & 0 \\ -0.5 & -0.5 & 1 & 0.5 & 0 & 0 & 0 & 0 & 0 & 0 \\ 0 & 0 & 0 & 0 & 0 & 0 & 0 & 0 & 0 & 0 \\ 1 & 2 & 0 & -1 & 1 & 0 & 0 & 0 & 0 & 0 \\ 0.5 & 0.5 & 0 & 0.5 & 0 & 1 & 0 & 0 & 0 & 0 \\ 0 & 0 & 0 & 0 & 0 & 0 & 1 & 0 & 0 & 0 \\ 0 & 0 & 0 & 0 & 0 & 0 & 0 & 1 & 0 & 0 \\ -0.5 & 0 & 0 & 0 & 0 & 0 & 0 & 0 & 1 & 0 \\ 0 & 0 & 0 & 0 & 0 & 0 & 0 & 0 & 0 & 1 \end{pmatrix} \begin{pmatrix} n_{react}^{H_2} \\ n_{in,neg}^{H_2O} \\ n_{in,neg}^{CH_4} \\ n_{in,neg}^{CO} \\ n_{in,neg}^{H_2} \\ n_{in,neg}^{CO_2} \\ n_{in,neg}^{O_2} \\ n_{in,neg}^{N_2} \\ n_{in,pos}^{O_2} \\ n_{in,pos}^{N_2} \end{pmatrix} + \begin{pmatrix} 10^{-6} \\ 0 \\ 0 \\ 0 \\ 0 \\ 0 \\ 0 \\ 0 \\ 0 \end{pmatrix} \quad \text{(S9)}$$

(3) The summation of the generated steam flow rate due to electrochemical reaction and the negatrode inlet steam flow rate is lower than the steam flow rate required for WGS reaction, i.e., $n_{react}^{H_2} + n_{in,neg}^{H_2O} < n_{in,neg}^{CO}$. This means that even CO cannot be entirely converted into H_2 and CO_2. In this case, the steam at the outlet will be exhausted, while its flow rate is set at 10^{-6} mol/s to be applied in the Nernst equation:

$$\begin{pmatrix} n_{out,neg}^{H_2O} \\ n_{out,neg}^{CH_4} \\ n_{out,neg}^{CO} \\ n_{out,neg}^{H_2} \\ n_{out,neg}^{CO_2} \\ n_{out,neg}^{O_2} \\ n_{out,neg}^{N_2} \\ n_{out,pos}^{O_2} \\ n_{out,pos}^{N_2} \end{pmatrix} = \begin{pmatrix} 0 & 0 & 0 & 0 & 0 & 0 & 0 & 0 & 0 & 0 \\ 0 & 0 & 1 & 0 & 0 & 0 & 0 & 0 & 0 & 0 \\ -1 & -1 & 0 & 1 & 0 & 0 & 0 & 0 & 0 & 0 \\ 0 & 0 & 0 & 0 & 1 & 0 & 0 & 0 & 0 & 0 \\ 1 & 1 & 0 & 0 & 0 & 1 & 0 & 0 & 0 & 0 \\ 0 & 0 & 0 & 0 & 0 & 0 & 1 & 0 & 0 & 0 \\ 0 & 0 & 0 & 0 & 0 & 0 & 0 & 1 & 0 & 0 \\ -0.5 & 0 & 0 & 0 & 0 & 0 & 0 & 0 & 1 & 0 \\ 0 & 0 & 0 & 0 & 0 & 0 & 0 & 0 & 0 & 1 \end{pmatrix} \begin{pmatrix} n_{react}^{H_2} \\ n_{in,neg}^{H_2O} \\ n_{in,neg}^{CH_4} \\ n_{in,neg}^{CO} \\ n_{in,neg}^{H_2} \\ n_{in,neg}^{CO_2} \\ n_{in,neg}^{O_2} \\ n_{in,neg}^{N_2} \\ n_{in,pos}^{O_2} \\ n_{in,pos}^{N_2} \end{pmatrix} + \begin{pmatrix} 10^{-6} \\ 0 \\ 0 \\ 0 \\ 0 \\ 0 \\ 0 \\ 0 \\ 0 \end{pmatrix} \quad \text{(S10)}$$

Note that, case 2 and case 3 will only appear at low current and low CPOX air flow rate, which are extreme cases and far away from the plant optimum and nominal operating points. Adding these two cases will not improve the optimal performance of the plant but increase the robustness of the model by preventing simulation crashes when the plant is operating under extreme operating conditions or startup/shutdown processes.

Energy balance. As an ideal CSTR, the outlet temperature of the stack equals the temperature inside the reactor.

According to how the stack is positioned inside the system's box, (1) the convection heat transfer from the CPOX, (2) the convection heat transfer from the ambient, and (3) the convection heat transfer from the afterburner are considered as heat loss. In this case, the energy balance can be written as the summation of the energy flowing in and out from the stack via the streams as well as the heat loss due to the convection heat transfer and heat radiation. The energy balance is expressed as below:

$$\begin{aligned} m_{stack} C_{p,stack} \frac{dT_{stack}}{dt} &= \sum_i n_{in,neg}^i H_{in,neg}^i(T_{cpox}) + \left(n_{in,pos}^{O_2} H_{in,pos}^{O_2}(T_{air}) + n_{in,pos}^{N_2} H_{in,pos}^{N_2}(T_{air})\right) \\ &- \sum_j n_{out,stack}^j H_{out,stack}^j(T_{stack}) - P_{stack} - Q_{loss,stack} \\ Q_{loss,stack} &= F_{conv,cpox}^{stack}(T_{stack} - T_{cpox}) + F_{conv,pcb}^{stack}(T_{stack} - T_{pcb}) + F_{conv,burn}^{stack}(T_{stack} - T_{burn}) \end{aligned} \tag{S11}$$

where m_{stack} is the mass of the stack, $C_{p,stack}$ is the heat capacity of the stack, the $Q_{loss,stack}$ includes the convection heat from CPOX and ambient as well as the radiation heat from afterburner, $H_{in,neg}^i(T_{cpox})$ is the enthalpy of gas from CPOX, $H_{in,pos}^{O_2}(T_{air})$ is the enthalpy of O_2 in the positrode, $H_{in,pos}^{N_2}(T_{air})$ is the enthalpy of N_2 in the positrode, $H_{out,stack}^j(T_{stack})$ is the outlet gas enthalpy of the stack, $n_{in,an}^i$ is the mole flow rates of the inlet gas from CPOX, $n_{out,stack}^j$ is the mole flow rates of the stack outlet gas, i denotes the inlet gas species of stack, j denotes the outlet gas species of stack, $F_{conv,cpox}^{stack}$ is the coefficient for convection heat transfer between stack and CPOX, $F_{conv,pcb}^{stack}$ is the coefficient for convection heat transfer between stack and ambient, $F_{conv,burn}^{stack}$ is the coefficient for convection heat transfer between stack and afterburner. T_{pcb} is the measured printed circuit board (PCB) temperature. Specifically, T_{air} is considered to be the temperature of the mixed flow between the cold air passing through the startup burner and the hot air from the air heat exchanger, which received the convection heat from the afterburner:

$$\begin{aligned} T_{air} &= T_{hexc} \times (1 - V_{air}) + T_{stburn} \times V_{air} + Q_{conv,burn}^{air} \\ Q_{conv,burn}^{air} &= \frac{F_{conv,burn}^{air} \times (T_{burn} - T_{air})}{n_{in,stack}^{air} \times C_{p,air}(T_{air})} \end{aligned} \tag{S12}$$

Where T_{hexc} is the outlet temperature of the cold fluid, i.e., air, T_{stburn} is the startup burner outlet temperature, $Q_{conv,burn}^{air}$ is the heat received by the air from the afterburner, $F_{conv,burn}^{air}$ is the coefficient for convection heat transfer between the air and the afterburner, T_{burn} is the outlet temperature of the afterburner, $C_{p,air}(T_{air})$ is the heat capacity of the air, which depends on the air temperature.

Stack voltage. The stack potential is mainly computed based on gas concentration, stack temperature and current with Nernst equation. Losses are considered in two aspects: (1) overpotentials due to activation, oxygen dissociation, electrolyte ohmic loss, gas diffusion, and ohmic loss from metal interconnector, and (2) power losses due to the internal electronic devices, air blower for the CPOX, air blower for the SOFC, nature gas blower, water pump, and inverter conversion. For the overpotential losses, instead of calculating each term, a lumped ASR model from Sunfire is applied. The calculation of the stack potential is expressed below:

$$U_{stack} = \left(U_{nernst} - \frac{C}{A_{active}} \times A_0 exp\left(\frac{E_A}{RT_{stack}}\right)\right) \times N_{cell} \tag{S13}$$

where U_{stack} is the stack voltage, U_{nernst} is the Nernst voltage, A_{active} is the cell electroactive area of 127.8 cm^2, $A_0 = 2.6 \times 10^{-8}$ [$\mathrm{Ohm \cdot m^2}$] and $E_A = 72900$ [J/mol] are the fitted parameters of the ASR model, R is the ideal gas constant, 8.314 $J \cdot K^{-1} \cdot mol^{-1}$.

The Nernst voltage (U_{nernst}) depends on the gas concentration in the stack. To simplify and accelerate the computation, outlet gas composition is considered. This assumption may lead to an inaccurate model voltage output, so another correction factor k_{nernst} is applied in the gas concentration part. The Nernst voltage is expressed as below:

$$U_{nernst} = U_s - dU_1 \times 75 - dU_2 \times (T_{stack} - 100) + \frac{RT_{stack}}{2F} \times \ln\left(\frac{x_{out,neg}^{H_2} \times \left(x_{out,pos}^{O_2}\right)^{0.5}}{x_{out,neg}^{H_2O} \times k_{nernst}}\right) \quad \text{(S14)}$$

where U_s is the standard redox potential, $dU_1 = 846\ \mu V/K$ is the voltage drop due the temperature increase from room temperature (25 ºC to 100 ºC), $dU_2 = 230\ \mu V/K$ is the voltage drop due to the temperature increase from stack temperature to the evaporation temperature, which, in our case, will be 100 ºC at 1 bar, $x_{out,neg}^{H_2}$ is the H_2 molar fraction in the outlet gas of negatrode, $x_{out,pos}^{O_2}$ is the O_2 molar fraction in the outlet gas of positrode, $x_{out,neg}^{H_2O}$ is the steam molar fraction in the outlet gas of negatrode.

Another two main stack indexes are fuel utilization ratio (FU) and air utilization ratio (AU), respectively, which are expressed below:

$$FU = \frac{n_{reac}^{CH_4}}{n_{in,cpox}^{CH_4}} = \frac{\frac{C \times N_{cell}}{8F}}{n_{in,cpox}^{CH_4} - \frac{n_{in,cpox}^{O_2}}{2}}$$
$$AU = \frac{N_{cell}}{8F}\frac{2C}{n_{in,pos}^{O_2}} \quad \text{(S15)}$$

3. Afterburner modelling

All the unreacted fuel will be combusted with excess air from the cathode in the burner after the stack. The heat generated in the burner will be used to heat up the air for the stack cathode to over 750 ºC and the living water from room temperature to around 60 ºC. This leads to higher system CHP efficiency. The main reaction happens inside the chamber and is written below:

$$H_2 + \frac{1}{2}O_2 \Rightarrow H_2O$$
$$CO + \frac{1}{2}O_2 \Rightarrow CO_2 \quad \text{(S16)}$$
$$CH_4 + 2O_2 \Rightarrow CO_2 + 2H_2O$$

Mass balance. Three reactions above are complete, in which case, the mass balance can be expressed as below:

$$\begin{pmatrix} n_{out,burn}^{H_2O} \\ n_{out,burn}^{CH_4} \\ n_{out,burn}^{CO} \\ n_{out,burn}^{H_2} \\ n_{out,burn}^{CO_2} \\ n_{out,burn}^{O_2} \\ n_{out,burn}^{N_2} \end{pmatrix} = \begin{pmatrix} 2 & 0 & 0 & 0 & 0 \\ 0 & 0 & 0 & 0 & 0 \\ 0 & 0 & 0 & 0 & 0 \\ 0 & 0 & 0 & 0 & 0 \\ 1 & 0 & 0 & 0 & 0 \\ -2 & 1 & 0 & 1 & 0 \\ 0 & 0 & 1 & 0 & 1 \end{pmatrix} \begin{pmatrix} n_{in,cpox}^{CH_4} \\ n_{in,cpox}^{O_2} \\ n_{in,cpox}^{N_2} \\ n_{in,pos}^{O_2} \\ n_{in,pos}^{N_2} \end{pmatrix} \quad \text{(S17)}$$

Energy balance. Playing the trade-off between computational speed and model accuracy, we model the afterburner as a CSTR with constant pressure and ideal gas mixing, whose outlet temperature is consistent with the chamber temperature. The energy balance is written as below:

$$m_{burn}C_{p,burn}\frac{dT_{burn}}{dt} = \sum_i n^i_{in,burn}H^i_{in,burn}(T_{stack}) - \sum_j n^j_{out,burn}H^j_{out,burn}(T_{burn}) - Q_{loss,burn} \quad \text{(S18)}$$
$$Q_{loss,burn} = F^{burn}_{conv,stack}(T_{burn} - T_{stack}) + F^{burn}_{conv,stburn}(T_{burn} - T_{stburn}),$$

where m_{burn} is the afterburner mass, $C_{p,burn}$ is the heat capacity of the burner, $Q_{loss,burn}$ is the heat exchange between afterburner and stack as well as the startup burner, $F^{burn}_{conv,stack}$ is the coefficient for convection heat transfer between afterburner and stack, $F^{burn}_{conv,stburn}$ is the coefficient for the convection heat transfer between afterburner and startup burner, $H^i_{in,burn}(T_{stack})$ is the inlet gas enthalpy of the afterburner, $H^j_{out,burn}(T_{burn})$ is the outlet gas enthalpy of the afterburner, $n^i_{in,burn}$ is the mole flow rates of the afterburner inlet gas, $n^j_{out,burn}$ is the mole flow rates of the afterburner outlet gas, i denotes the inlet gas species of the afterburner, j denotes the outlet gas species of the afterburner.

4. Startup burner modelling

Startup burner is dedicated for the heat-up process, which is not working during the nominal operating conditions. Hence, mass balance is not considered here, as no chemical reaction occurs during the nominal conditions.

Energy balance considers the convection heat transfer between the startup burner and the ambient and the convection heat transfer between the startup burner and the afterburner, as is expressed below:

$$m_{stburn}C_{p,stburn}\frac{dT_{stburn}}{dt} = \sum_i n^i_{in,stburn}H^i_{in,stburn}(T_{in}) - \sum_j n^j_{out,stburn}H^j_{out,stburn}(T_{stburn}) - Q_{loss,stburn} \quad \text{(S19)}$$
$$Q_{loss,stburn} = F^{stburn}_{conv,pcb}\left(T_{stburn} - T_{pcb}\right) + F^{stburn}_{conv,burn}(T_{stburn} - T_{burn})$$

where m_{stburn} is the startup burner mass, $C_{p,stburn}$ is the heat capacity of the startup burner, $Q_{loss,stburn}$ is the heat exchange between startup burner and ambient as well as the afterburner, $F^{stburn}_{conv,pcb}$ is the coefficient for thermal radiation heat between afterburner and stack, $F^{stburn}_{conv,burn}$ is the coefficient for the convection heat transfer between startup burner and afterburner, $H^i_{in,st\ burn}(T_{in})$ is the inlet gas enthalpy of the startup burner, $H^j_{out,burn}(T_{stburn})$ is the outlet gas enthalpy of the startup burner, $n^i_{in,burn}$ is the mole flow rates of the startup burner inlet gas, $n^j_{out,burn}$ is the mole flow rates of the startup burner outlet gas, i denotes the inlet gas species of the startup burner, j denotes the outlet gas species of the startup burner. Notably, there are only two types of gas through startup burner, O_2 and N_2.

5. Air heat exchanger modelling

The heat exchanger model is simplified using ε-NTU (effectiveness-number of transfer units) method, which increased the model robustness by avoiding the temperature cross in log mean temperature difference (LMTD) method.

Energy balance is calculated by considering both the hot flow (exhaust from afterburner) and cold flow (cold air), as is written below:

$$m_{hexh}C_{p,hexh}\frac{dT_{hexh}}{dt} = -Q_{NTU,air} + \sum_i n^i_{in,hexh}H^i_{in,hexh}(T_{burn}) - \sum_j n^j_{out,hexh}H^j_{out,hexh}(T_{hexh})$$

$$\left(m_{hexc}C_{p,hexc} + m_{est}C_{p,est}\right)\frac{dT_{hexc}}{dt} = Q_{NTU,air} + \sum_i n^i_{in,hexc}H^i_{in,hexc}\left(T_{pcb}\right) - \sum_j n^j_{out,hexc}H^j_{out,hexc}(T_{hexc})$$

$$Q_{NTU,air} = C_{min,air} \times \left(T_{burn} - T_{pcb}\right) \times \epsilon_{hex,air} \tag{S20}$$

$$C_{min,air} = \min[m_{hexh,gas}C_{p,hexh,gas}, m_{hexc,gas}C_{p,hexc,gas}]$$

$$C_{p,hexh,gas} = \sum_i x^i_{in,hexh}c^i_{p,hexh}(T_{hexh})$$

$$C_{p,hexc,gas} = \sum_i x^i_{in,hexc}c^i_{p,hexc}(T_{hexc})$$

where m_{hexh} and m_{hexc} are the flow mass of the hot side and cold side, respectively, $C_{p,hexh}$ and $C_{p,hexc}$ are the heat capacities of the hot fluid and cold fluid of the heat exchanger, $m_{est}C_{p,est}$ is the additional parameter to be estimated for the dynamic behavior of the air HEX. $H^i_{in,hexh}(T_{burn})$ is the inlet gas enthalpy of the hot flow, $H^j_{out,hexh}(T_{hexh})$ is the outlet gas enthalpy of the hot flow, $n^i_{in,hexh}$ is the mole flow rates of the hot side inlet gas, $n^j_{out,hexh}$ is the mole flow rates of the hot side outlet gas, $H^i_{in,hexc}(T_{in})$ is the inlet gas enthalpy of the hot flow, which is at room temperature of 25 °C, $H^j_{out,hexc}(T_{hexc})$ is the outlet gas enthalpy of the hot flow, $n^i_{in,hexc}$ is the mole flow rates of the cold flow inlet gas, $n^j_{out,hexc}$ is the mole flow rates of the cold flow outlet gas, i denotes the inlet gas species, j denotes the outlet gas species, $Q_{NTU,air}$ is the heat transfer between the hot side and the cold side, $C_{min,air}$ is the lower value between the heat capacities of cold flow and hot flow, $\epsilon_{hex,air}$ the ratio between the actual heat transfer rate and the maximum possible heat transfer rate, $C_{p,hexh,gas}$ is the gas heat capacity in the hot side, $x^i_{in,hexh}$ is the mole fraction of the individual gas in the hot side, $c^i_{p,hexh}(T_{hexh})$ is the heat capacity of the individual gas in the hot side, $C_{p,hexc,gas}$ is the gas heat capacity in the cold side, $x^i_{in,hexc}$ is the mole fraction of the individual gas in the cold side, $c^i_{p,hexc}(T_{hexc})$ is the heat capacity of the individual gas in the cold side.

6. Water heat exchanger modelling

Similar to the air heat exchanger, water heat exchanger is modelled based on the ε-NTU method. The energy balance is considered as below:

$$m_{whexh}C_{p,whexh}\frac{dT_{whexh}}{dt} = -Q_{NTU,H_2O} + \sum_i n^i_{in,whexh}H^i_{in,whexh}(T_{hexh}) - \sum_j n^j_{out,whexh}H^j_{out,whexh}(T_{whexh})$$

$$m_{whexc}C_{p,whexc}\frac{dT_{whexc}}{dt} = Q_{NTU,H_2O} + n^{H_2O}_{in,whexc}H^{H_2O}_{in,whexc}\left(T_{in,H_2O}\right) - n^{H_2O}_{out,whexc}H^{H_2O}_{out,whexc}\left(T_{out,H_2O}\right) \tag{S21}$$

$$Q_{NTU,H_2O} = C_{min,H_2O} \times \left(T_{hexh} - T_{in,H_2O}\right) \times \epsilon_{hex,H_2O}$$

$$C_{min,H_2O} = \min[m_{whexh,gas}C_{p,whexh,gas}, m_{hexc,H_2O}C_{p,hexc,H_2O}(T_{out,H_2O})]$$

$$C_{p,whexh,gas} = \sum_i x^i_{in,whexh}c^i_{p,whexh}(T_{whexh})$$

where m_{whexh} and m_{whexc} are the mass flow of the hot side and cold side, respectively, $C_{p,whexh}$ and $C_{p,whexc}$ are the heat capacities of the hot fluid and cold fluid of the heat exchanger. $H^i_{in,w\,hexh}(T_{hexh})$ is the inlet gas enthalpy of the hot flow, $H^j_{out,whexh}(T_{whexh})$ is the outlet gas enthalpy of the hot flow, $n^i_{in,whexh}$ is the mole flow rates of the hot side inlet gas, $n^j_{out,whexh}$ is the mole flow rates of the hot side outlet gas, $H^{H_2O}_{in,whexc}\left(T_{in,H_2O}\right)$ is the inlet gas enthalpy of the water, which is measured by temperature sensor, $H^{H_2O}_{out,whexc}\left(T_{out,H_2O}\right)$ is the outlet gas enthalpy of the water, $n^{H_2O}_{in,whexc}$ is the mole flow rates of water, $n^{H_2O}_{out,whexc}$ is the mole flow rates of the water, i denotes the inlet gas species, j denotes the outlet gas species, Q_{NTU,H_2O} is the heat transfer between the hot side and the cold side, C_{min,H_2O} is the lower value between the heat capacities of cold flow and hot flow, ϵ_{hex,H_2O} the ratio between the actual heat transfer rate and the maximum possible heat transfer rate, $C_{p,whexh,gas}$ is the gas heat capacity in the hot side, $x^i_{in,whexh}$ is the mole fraction of the individual gas in the hot side, $c^i_{p,whexh}(T_{whexh})$ is the heat capacity of the individual gas in the hot side, $C_{p,whexc,H_2O}$ is the water heat capacity in the cold side.

Part B. Parameter estimation for simplified dynamic system model

The parameter estimation of the system model was performed via a fmincon function-based MATLAB code developed by EPFL, Group of Energy Materials. Two steps were considered, including (1) parameter estimation of individual components, where the temperatures of the components that have heat transfer with the estimated component directly use the plant measurements, and (2) parameter estimation of the whole system. The accuracy of the model is calculated based on the weighted least squares between the model outputs and the plant measurements as follows:

$$min\ SQ = \sum k \sum \left(y^j - Y^j(u, p)\right)^2 \tag{S22}$$

$$\boldsymbol{p}_i^L \leq \boldsymbol{p}_i \leq \boldsymbol{p}_i^U$$

where SQ is the sum of squares of the deviations of the model output and the plant measurement, k_{pe}^i is a scaling factor used to keep the difference of all the outputs at the same magnitude, y_j^i is the plant measurement, Y_j^i is the model output, $\boldsymbol{u}_i$ is the vector of inputs, $\boldsymbol{p}_i$ is the vector of the parameters to be estimated, $\boldsymbol{p}_i^L$ is the lower bound vector of the parameters, $\boldsymbol{p}_i^U$ is the upper bound vector of the parameters, i denotes the outputs, j denotes the time point selected. Notably, the upper bound and lower bound of the estimated parameters are flexible during the optimization, where the upper bound is typically initialized at ten times the initial guess and the lower bound is usually initialized at one-tenth of the initial guess.

1. CPOX model

Note that we need to solve a differential equation in order to solve the following optimization problem. The inputs of CPOX are the CPOX air blower workload and the natural gas flow rate, while the corresponding model output is the CPOX outlet temperature. Hence, the optimization problem for the parameter estimation of the CPOX can be expressed as follow:

$$\begin{aligned}
&\min_{\boldsymbol{p}_{cpox}} SQ_{cpox} = \sum_j \left(T_{p,cpox}^j - T_{m,cpox}^j(\boldsymbol{u}_{cpox}, \boldsymbol{p}_{cpox})\right)^2 \\
&\text{s.t. Model equations of CPOX.} \\
&\quad \boldsymbol{p}_{cpox}^L \leq \boldsymbol{p}_{cpox} \leq \boldsymbol{p}_{cpox}^U \\
&\quad \boldsymbol{u}_{cpox} \in \left[WL_{CH_4}^j, WL_{air,cpox}^j, T_{p,stack}^j\right] \\
&\quad \boldsymbol{p}_{cpox} \in \left[F_{conv,amb}^{cpox}, T_{amb,cpox}, F_{air,cpox}, F_{WGS}\right]
\end{aligned} \tag{S23}$$

where $T_{p,cpox}^j$ is the plant measurement of CPOX outlet temperature, $T_{m,cpox}^j$ is the model output of CPOX outlet temperature, $\boldsymbol{u}_{cpox}$ is the input vector of the CPOX model parameter estimation, $\boldsymbol{p}_{cpox}$ is the vector of the parameters estimated in the CPOX model, $\boldsymbol{p}_{cpox}^L$ is the lower bound vector of the parameters, $\boldsymbol{p}_{cpox}^U$ is the upper bound vector of the parameters, j denotes the time points selected, $\boldsymbol{u}_{cpox}$ is the model inputs, including the natural gas blower workload ($WL_{CH_4}^j$), CPOX air blower workload ($WL_{air,cpox}^j$), and stack outlet temperature measurement ($T_{p,stack}^j$) at time j.

2. Solid oxide fuel cell stack model

Voltage. Based on the assumptions and the Nernst equation, the stack voltage is related to the stack temperature and the outlet gas composition that can be predicted with the current input, measured stack temperature, positrode air blower workload, inlet natural gas blower workload, the CPOX air blower workload, and the assumptions made in the CPOX model. The optimization problem for the parameter estimation of the stack voltage can be formulated as below:

$$\begin{aligned}
&\min_{\boldsymbol{p}_{vol}} SQ_{voltage} = \sum_j \left(U_p^j - U_m^j(\boldsymbol{u}_{vol}, \boldsymbol{p}_{vol})\right)^2 \\
&\text{s.t. Model voltage equations} \\
&\quad \boldsymbol{p}_{vol}^L \leq \boldsymbol{p}_{vol} \leq \boldsymbol{p}_{vol}^U \\
&\quad \boldsymbol{u}_{vol} \in \left[C^j, WL_{CH_4}^j, WL_{air,cpox}^j, WL_{air,stack}^j, T_{p,stack}^j\right] \\
&\quad \boldsymbol{p}_{vol} \in [k_{nernst}]
\end{aligned} \tag{S24}$$

where U_p^j is the voltage measurement, U_m^j is the model voltage output, $\boldsymbol{p}_{vol}$ is the vector of the parameters

estimated for the voltage output, $\boldsymbol{p}_{vol}^{L}$ is the vector of lower bounds of the parameters, $\boldsymbol{p}_{vol}^{U}$ is the vector of upper bounds of the parameters, j is the index that denotes the time points selected based on the plant voltage change, $\boldsymbol{u}_{vol}$ is the model input vector for stack voltage parameter estimation.

Outlet temperature of the stack is calculated based on (1) CPOX outlet gas flow rates, (2) CPOX outlet gas temperature, (3) positrode air blower workload, (4) positrode inlet air temperature which refers to the plant measurement, and (5) the current. Hence, the optimization problem for the parameter estimation of the stack outlet temperature can be expressed as follows:

$$\begin{aligned}
&\min_{\boldsymbol{p}_{stack}} SQ_{stack} = \sum_j \left(T_{p,stack}^{j} - T_{m,stack}^{j}(\boldsymbol{u}_{stack}, \boldsymbol{p}_{stack})\right)^2 \\
&\text{s.t. Model equations of the stack and the CPOX} \\
&\quad \boldsymbol{p}_{stack}^{L} \le \boldsymbol{p}_{stack} \le \boldsymbol{p}_{stack}^{U} \\
&\quad \boldsymbol{u}_{stack} \in \left[C^j, WL_{CH_4}^{j}, WL_{air,cpox}^{j}, WL_{air,stack}^{j}, T_{p,cpox}^{j}, T_{p,pcb}^{j}, T_{p,burn}^{j}, T_{p,air}^{j}\right] \\
&\quad \boldsymbol{p}_{stack} \in \left[F_{conv,pcb}^{stack}, F_{conv,burn}^{stack}\right]
\end{aligned} \tag{S25}$$

where $T_{m,stack}^{j}$ is the modelled stack model temperature, $T_{p,pcb}^{j}$ is plant measurement of the PCB temperature, $T_{p,burn}^{j}$ is the plant measurement of the afterburner outlet temperature, $T_{p,air}^{j}$ is the measured positrode inlet air temperature, $\boldsymbol{u}_{stack}$ is the input vector of the stack model during the parameter estimation for stack temperature, $\boldsymbol{p}_{stack}$ is the vector of the parameters estimated for the stack model temperature output, $\boldsymbol{p}_{stack}^{L}$ is the vector of lower bounds of the parameters, $\boldsymbol{p}_{stack}^{U}$ is the vector of upper bounds of the parameters, j denotes the time points selected as same as those in the parameter estimation for the voltage.

3. Afterburner model

The inputs of the afterburner are the outlet flow from the stack, which can be calculated according to the current, the fuel flow rate, the stack outlet gas temperature measurements, and the air flow rates of both CPOX and stack. The corresponding model output is the afterburner outlet temperature. Hence, the optimization problem for the parameter estimation of the afterburner model can be expressed as follows:

$$\begin{aligned}
&\min_{\boldsymbol{p}_{burn}} SQ_{burn} = \sum_j \left(T_{p,burn}^{j} - T_{m,burn}^{j}(\boldsymbol{u}_{burn}, \boldsymbol{p}_{burn})\right)^2 \\
&\text{s.t. Model equations of the afterburner, stack and CPOX} \\
&\quad \boldsymbol{p}_{burn}^{L} \le \boldsymbol{p}_{burn} \le \boldsymbol{p}_{burn}^{U} \\
&\quad \boldsymbol{u}_{burn} \in \left[C^j, WL_{CH_4}^{j}, WL_{air,cpox}^{j}, WL_{air,stack}^{j}, T_{p,stack}^{j}, T_{p,stburn}^{j}\right] \\
&\quad \boldsymbol{p}_{burn} \in \left[F_{conv,stack}^{burn}, F_{conv,stburn}^{burn}\right]
\end{aligned} \tag{S26}$$

where $T_{m,burn}^{j}$ is the model output of afterburner outlet temperature, $T_{p,stburn}^{j}$ is the startup burner outlet temperature, $\boldsymbol{u}_{burn}$ is the input vector of the afterburner model parameter estimation, $\boldsymbol{p}_{burn}$ is the vector of the parameters estimated in the afterburner model, $\boldsymbol{p}_{burn}^{L}$and $\boldsymbol{p}_{burn}^{U}$ are the vectors of lower and upper bound values for the parameters, j denotes the time points selected.

4. Startup burner model

The inputs of the startup burner are split ratio of the positrode air, the positrode air flow rates, the PCB temperature measurements, and the afterburner temperature measurements. The corresponding model output is the startup burner outlet temperature. Hence, the optimization problem for the parameter estimation of the

afterburner model can be expressed as follows:

$$\min_{\boldsymbol{p}_{stburn}} SQ_{stburn} = \sum_j \left(T^j_{p,stburn} - T^j_{m,stburn}(\boldsymbol{u}_{stburn}, \boldsymbol{p}_{stburn})\right)^2$$
$$\text{s.t. Model equations of the startup burner}$$
$$\boldsymbol{p}^L_{stburn} \le \boldsymbol{p}_{stburn} \le \boldsymbol{p}^U_{stburn}$$
$$\boldsymbol{u}_{stburn} \in \left[WL^j_{air,stack}, V^j_{air}, T^j_{p,pcb}, T^j_{p,burn}\right]$$
$$\boldsymbol{p}_{stburn} \in \left[F^{stburn}_{conv,pcb}, F^{stburn}_{conv,burn}\right] \quad \text{(S27)}$$

where $T^j_{m,stburn}$ is the model output of afterburner outlet temperature, $\boldsymbol{u}_{stburn}$ is the input vector of the startup burner model parameter estimation, $\boldsymbol{p}_{stburn}$ is the vector of the parameters estimated in the startup burner model, $\boldsymbol{p}^L_{stburn}$and $\boldsymbol{p}^U_{stburn}$ are the vectors of lower and upper bound values for the parameters, j denotes the time points selected.

5. Air heat exchanger model

The inputs of the air HEX include split ratio of the positrode air, the positrode air flow rates, the CPOX air flow rate, fuel flow rate, the afterburner temperature measurements, the startup burner temperature, and the inlet air temperature which is set at PCB temperature. The corresponding model output will be the temperature of the mixed air. The optimization problem is then written as below:

$$\min_{\boldsymbol{p}_{hex}} SQ_{hex} = \sum_j \left(T^j_{p,air} - T^j_{m,air}(\boldsymbol{u}_{hex}, \boldsymbol{p}_{hex})\right)^2$$
$$\text{s.t. Model equations of air HEX}$$
$$\boldsymbol{p}^L_{hex} \le \boldsymbol{p}_{hex} \le \boldsymbol{p}^U_{hex}$$
$$\boldsymbol{u}_{hex} \in \left[WL^j_{CH_4}, WL^j_{air,cpox}, WL^j_{air,stack}, V^j_{air}, T^j_{p,burn}, T^j_{p,stburn}\right]$$
$$\boldsymbol{p}_{hex} \in \left[F^{air}_{conv,burn}, \epsilon_{hex,air}\right] \quad \text{(S28)}$$

where $T^j_{m,hex}$ is the model temperature output of the air from the air HEX, $\boldsymbol{u}_{hex}$ is the input vector of the air HEX parameter estimation, $\boldsymbol{p}_{hex}$ is the vector of the parameters estimated in the afterburner model, $\boldsymbol{p}^L_{hex}$ is the lower bound vector of the parameters, $\boldsymbol{p}^U_{hex}$ is the upper bound vector of the parameters, j denotes the time points selected.

6. Water heat exchanger model

The inputs of the water HEX include water pump workload, inlet water temperature, and the outlet temperature of the air HEX hot side, which can be calculated with the air HEX model and the afterburner outlet temperature. The corresponding model output will be the temperature of the mixed air. The optimization problem is then written as below:

$$\min_{\boldsymbol{p}_{whex}} SQ_{whex} = \sum_j \left(T^j_{p,out,H_2O} - T^j_{m,out,H_2O}(\boldsymbol{u}_{whex}, \boldsymbol{p}_{whex})\right)^2$$
$$\text{s.t. Model equations of water HEX and air HEX}$$
$$\boldsymbol{p}^L_{whex} \le \boldsymbol{p}_{whex} \le \boldsymbol{p}^U_{whex}$$
$$\boldsymbol{u}_{whex} \in \left[WL^j_{CH_4}, WL^j_{air,cpox}, WL^j_{air,stack}, V^j_{air}, WL^j_{H_2O}, T^j_{in,H_2O}, T^j_{p,burn}\right]$$
$$\boldsymbol{p}_{whex} \in \left[\epsilon_{hex,H_2O}\right] \quad \text{(S29)}$$

where T^j_{p,out,H_2O} is the plant measurements of the outlet water temperature from the water HEX, T^j_{m,out,H_2O} is the model temperature output of the water from the water HEX, $\boldsymbol{u}_{whex}$ is the input vector of the water HEX

model parameter estimation, $\boldsymbol{p}_{whex}$ is the vector of the parameters estimated in the afterburner model, $\boldsymbol{p}_{whex}^{L}$ is the lower bound vector of the parameters, $\boldsymbol{p}_{whex}^{U}$ is the upper bound vector of the parameters, j denotes the time points selected.

7. Integrated system model

The inputs of the whole system model include the natural gas blower workload, CPOX air blower workload, positrode air blower workload, current, air mixing valve ratio, and water pump workload. As the water HEX is isolated from the other components without heat interactions, its parameters were not estimated to reduce the computation load. The optimization problem is written as below:

$$
\begin{aligned}
\min_{\boldsymbol{p}_{sys}} SQ_{sys} = & \sum_{j}\left(T_{p,cpox}^{j} - T_{m,cpox}^{j}(\boldsymbol{u}_{sys}, \boldsymbol{p}_{sys})\right)^2 + \sum_{j}\left(T_{p,stack}^{j} - T_{m,stack}^{j}(\boldsymbol{u}_{sys}, \boldsymbol{p}_{sys})\right)^2 \\
& + \sum_{j}\left(T_{p,burn}^{j} - T_{m,burn}^{j}(\boldsymbol{u}_{sys}, \boldsymbol{p}_{sys})\right)^2 \\
& + \sum_{j}\left(T_{p,stburn}^{j} - T_{m,stburn}^{j}(\boldsymbol{u}_{sys}, \boldsymbol{p}_{sys})\right)^2 + \sum_{j}\left(T_{p,air}^{j} - T_{m,air}^{j}(\boldsymbol{u}_{sys}, \boldsymbol{p}_{sys})\right)^2
\end{aligned}
$$

s.t. Model equations of CPOX, stack, afterburner, startup burner, air HEX and water HEX

$$
\boldsymbol{p}_{sys}^{L} \le \boldsymbol{p}_{sys} \le \boldsymbol{p}_{sys}^{U} \qquad \text{(S30)}
$$

$$
\boldsymbol{u}_{sys} \in \left[WL_{CH_4}^{j}, WL_{air,cpox}^{j}, WL_{air,stack}^{j}, C^{j}, V_{air}^{j}, WL_{H_2O}^{j}, T_{pcb}^{j}\right]
$$

$$
\boldsymbol{p}_{sys} \in \begin{bmatrix} F_{conv,amb}^{cpox}, T_{amb,cpox}, F_{air,cpox}, F_{WGS}, \\ F_{conv,pcb}^{stack}, F_{conv,burn}^{stack}, F_{air,stack,exp}, F_{air,stack,pre}, m_{stack}C_{p,stack}, \\ F_{conv,stburn}^{burn}, F_{conv,stack}^{burn}, m_{burn}C_{p,burn}, \\ F_{conv,pcb}^{stburn}, F_{conv,burn}^{stburn}, m_{stburn}C_{p,stburn}, \\ \epsilon_{hex,air}, F_{conv,burn}^{air}, m_{est}C_{p,est} \end{bmatrix}
$$

where $\boldsymbol{u}_{sys}$ is the input vector of the system model parameter estimation, $\boldsymbol{p}_{sys}$ is the vector of the parameters estimated in the system model, $\boldsymbol{p}_{sys}^{L}$ is the lower bound vector of the parameters, $\boldsymbol{p}_{sys}^{U}$ is the upper bound vector of the parameters, j denotes the time points selected. Notably, the final model will be available for sharing upon request.

Part C. System test matrix and model parameter estimation

A test matrix with 34 operating points has been proposed and implemented to obtain enough dynamic data for model calibration. As is shown in Table S1, starting from the steady state of previous operating condition, every test only changed one input variable to exclude the coupled interference from two input variables. Each input variable has been perturbed for at least 3 time. All the test datasets have been separated into two parts, one part for parameter estimation (PE), and another part for validation (VD), and the comparison between the model and the plant measurements is shown in Figure S1-Figure S7. It can clearly be observed that the model response considerably deviated from the plant measurement.

Table S1. Test matrix for system performance characterization under different operating conditions, where the dash means that the input variable remains unchanged.

**Test No.**	**1**	**2**	**3**	**4**	**5**	**6**	**7**	**8**	**9**	**10**	**11**	**12**
$\boldsymbol{WL}_{\mathbf{CH_4}}$ **[%]**	95	-	-	-	95	-	-	-	-	-	95	-
$\boldsymbol{WL}_{\mathbf{air,cpox}}$ **[%]**	71.2	-	-	-	71.2	-	-	-	61.2	-	71.6	-
$\boldsymbol{WL}_{\mathbf{air,stack}}$ **[%]**	40.3	-	-	-	39.9	-	-	45	-	-	36.2	-
$\boldsymbol{I}$ **[A]**	18	20	18	16	18	19	16	-	-	-	18	-
$\boldsymbol{WL}_{\mathbf{H_2O}}$ **[%]**	23.6	-	-	-	23.6	-	-	-	-	-	22.6	-

V_{air} [%]	27	-	-	-	26.9	-	-	-	-	32	27	30.4
**Test No.**	**13**	**14**	**15**	**16**	**17**	**18**	**19**	**20**	**21**	**22**	**23**	**24**
WL_{CH_4} [%]	-	-	-	90	-	-	-	-	-	85	-	85
$WL_{air,cpox}$ [%]	-	76.8	-	-	-	-	-	65.4	-	64.5	-	63.2
$WL_{air,stack}$ [%]	-	-	31.2	-	-	40.4	-	-	-	42.7	-	37.6
I [A]	-	-	-	-	15	-	-	-	-	18	16	16
WL_{H_2O} [%]	31.7	-	-	-	-	-	-	-	-	20.3	-	20.3
V_{air} [%]	-	-	-	-	-	-	20	-	15	13.4	-	22
**Test No.**	**25**	**26**	**27**	**28**	**29**	**30**	**31**	**32**	**33**	**34**		
WL_{CH_4} [%]	75	75	-	-	-	65	65	-	65	-		
$WL_{air,cpox}$ [%]	-	57.5	-	-	-	-	50.2	-	50.2	-		
$WL_{air,stack}$ [%]	-	31.7	-	-	-	-	31.9	-	26	-		
I [A]	-	16	14	12	10	-	10	-	10	-		
WL_{H_2O} [%]	-	20.3	-	-	-	-	20.3	35	35	60		
V_{air} [%]	-	21.9	-	-	-	-	18.2	-	22.1	-		

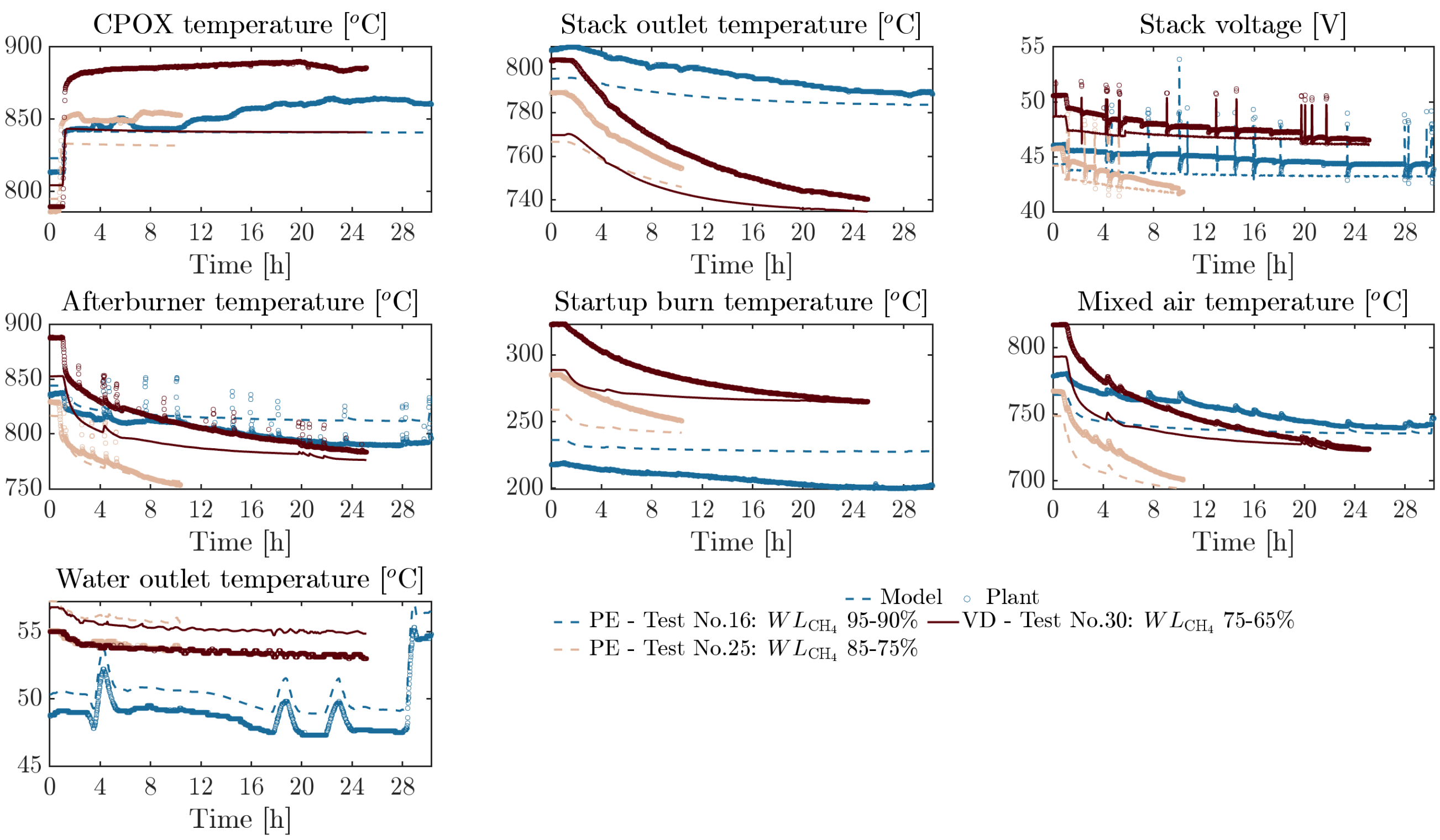

Figure S1. Comparison of dynamic behavior between the model results (lines) and the plant measurements (circles) under different natural gas blower workloads with other input variables unchanged, where the experimantal data applied for parameter estimation is in dash lines and the experimental data for model validation is in solid lines.

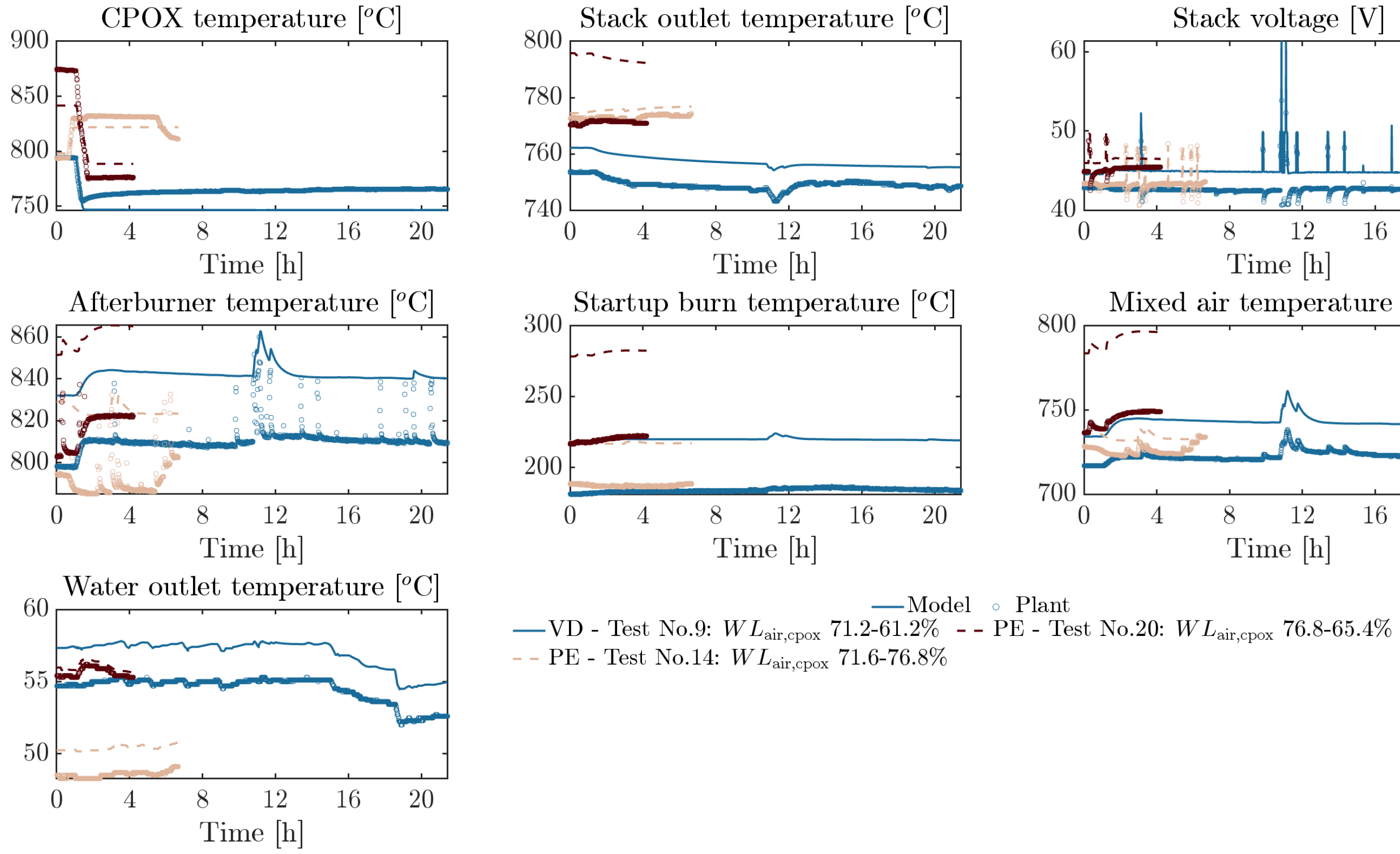

Figure S2. Comparison of dynamic behavior between the model results (lines) and the plant measurements (circles) under different CPOX air blower workloads with other input variables unchanged, where the experimantal data applied for parameter estimation is in dash lines and the experimental data for model validation is in solid lines.

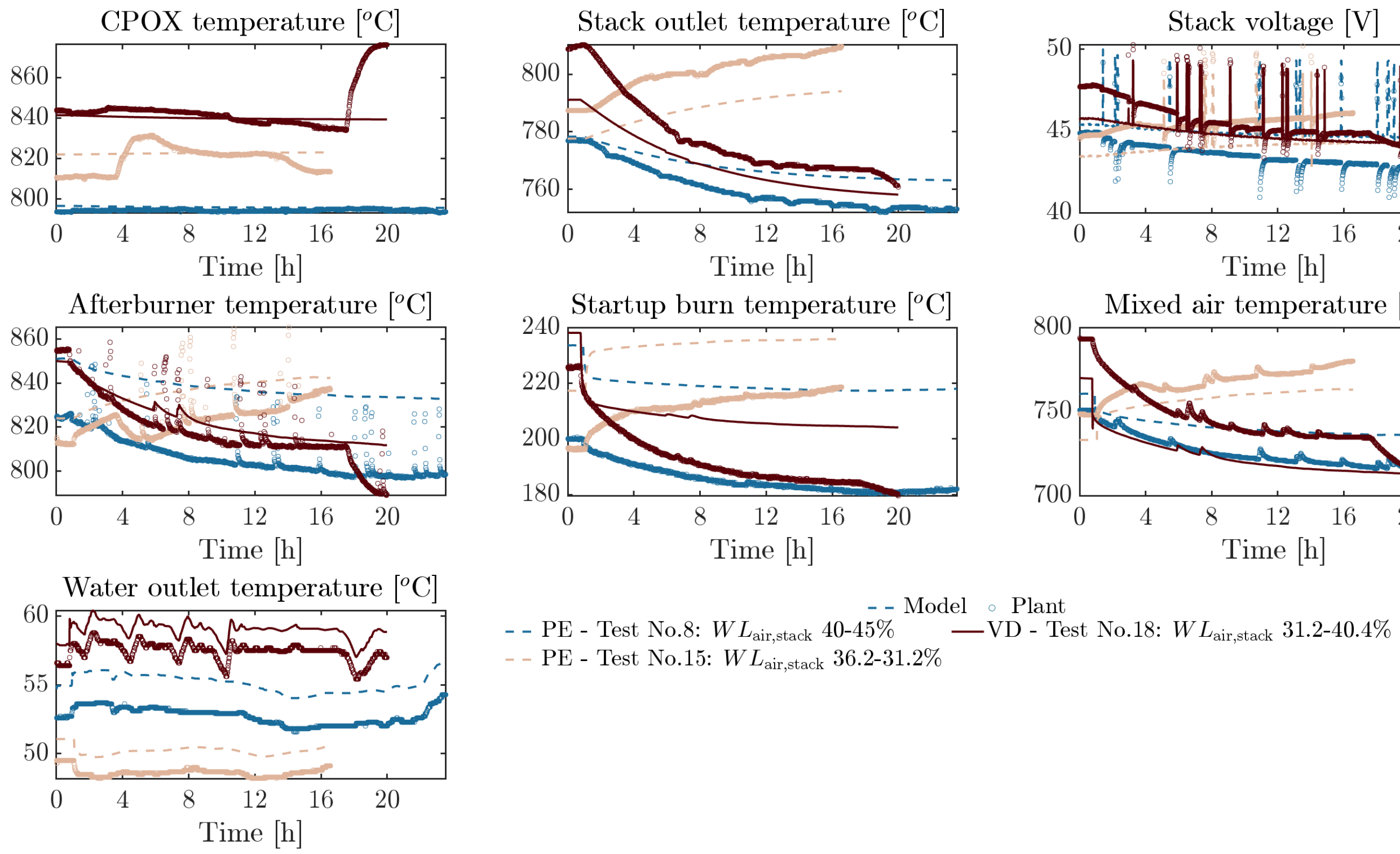

Figure S3. Comparison of dynamic behavior between the model results (lines) and the plant measurements (circles) under different positrode air blower workloads with other input variables unchanged, where the experimantal data applied for parameter estimation is in dash lines and the experimental data for model validation is in solid lines.

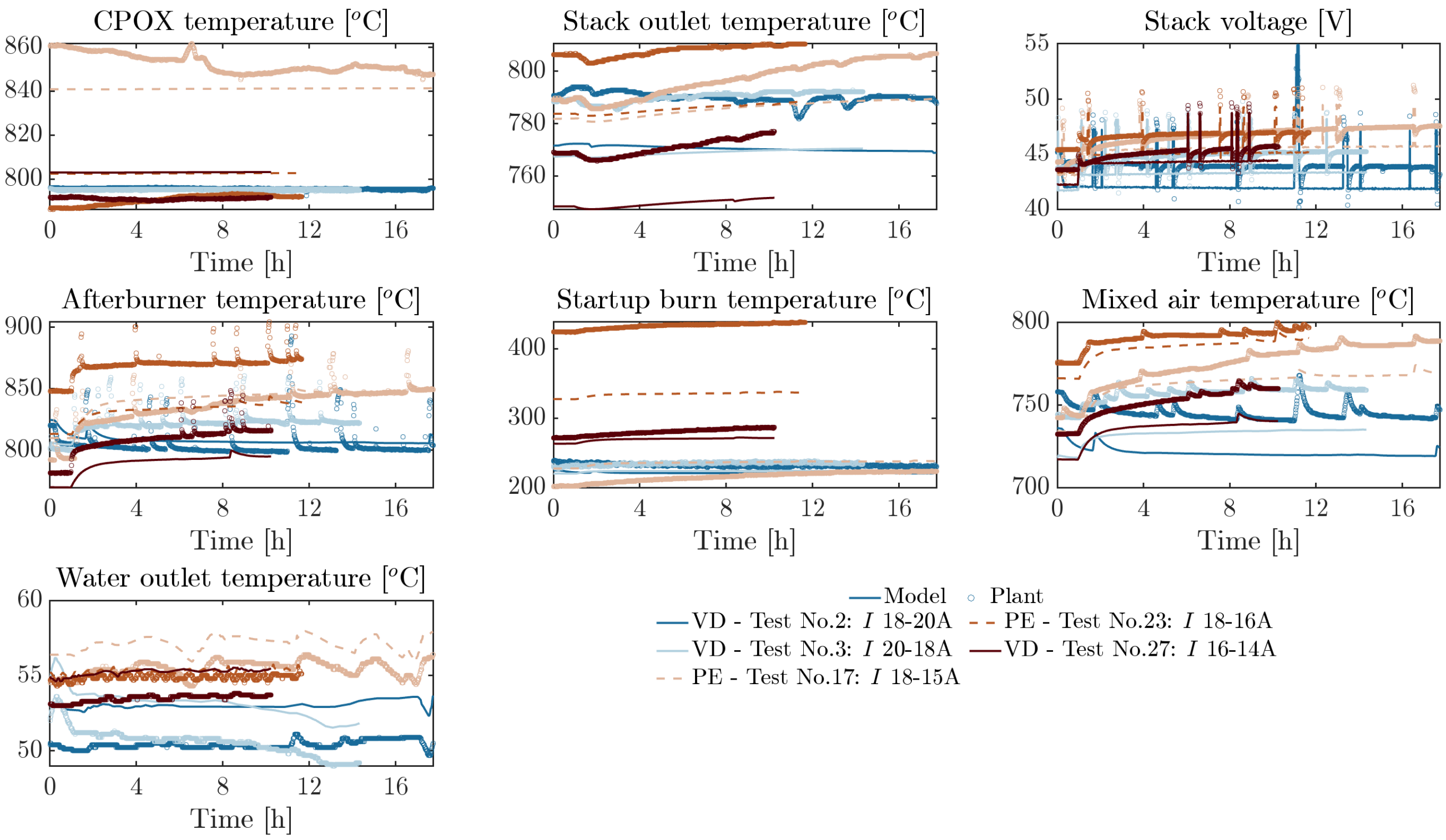

Figure S4. Comparison of dynamic behavior between the model results (lines) and the plant measurements (circles) under different current with other input variables unchanged, where the experimantal data applied for parameter estimation is in dash lines and the experimental data for model validation is in solid lines.

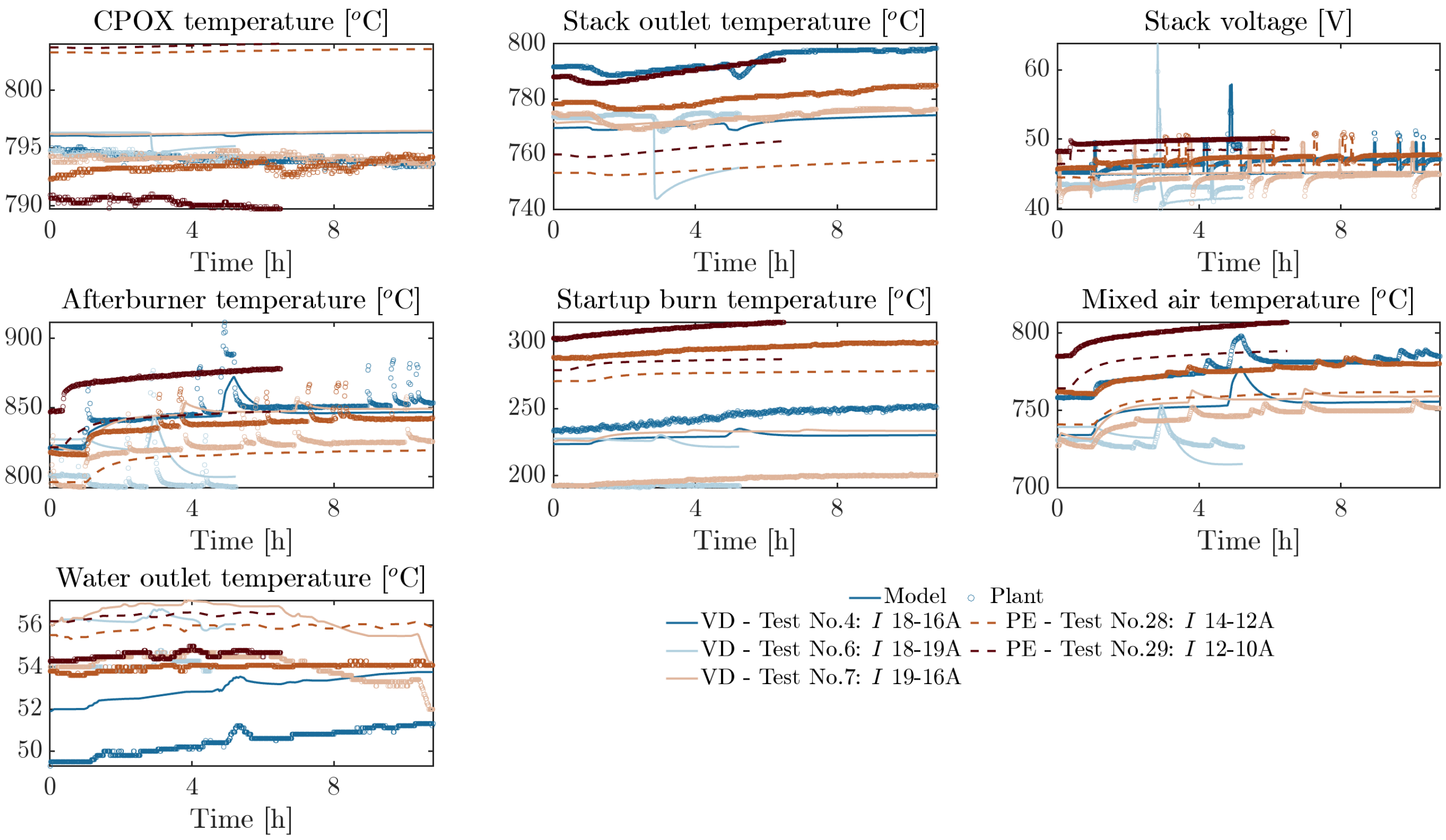

Figure S5. Comparison of dynamic behavior between the model results (lines) and the plant measurements (circles) under different current with other input variables unchanged, where the experimantal data applied for parameter estimation is in dash lines and the experimental data for model validation is in solid lines.

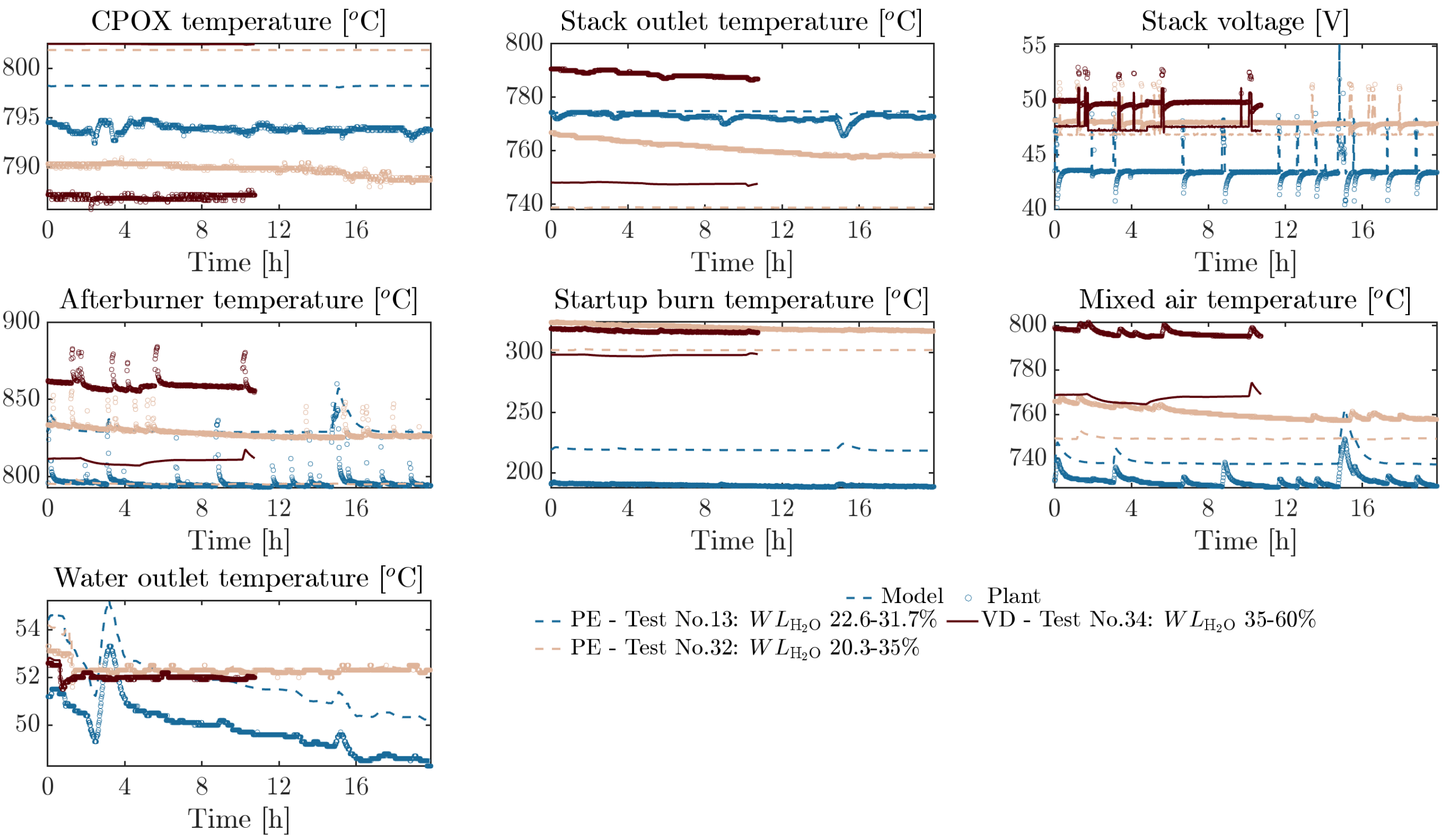

Figure S6. Comparison of dynamic behavior between the model results (lines) and the plant measurements (circles) under different water pump workload with other input variables unchanged, where the experimantal data applied for parameter estimation is in dash lines and the experimental data for model validation is in solid lines.

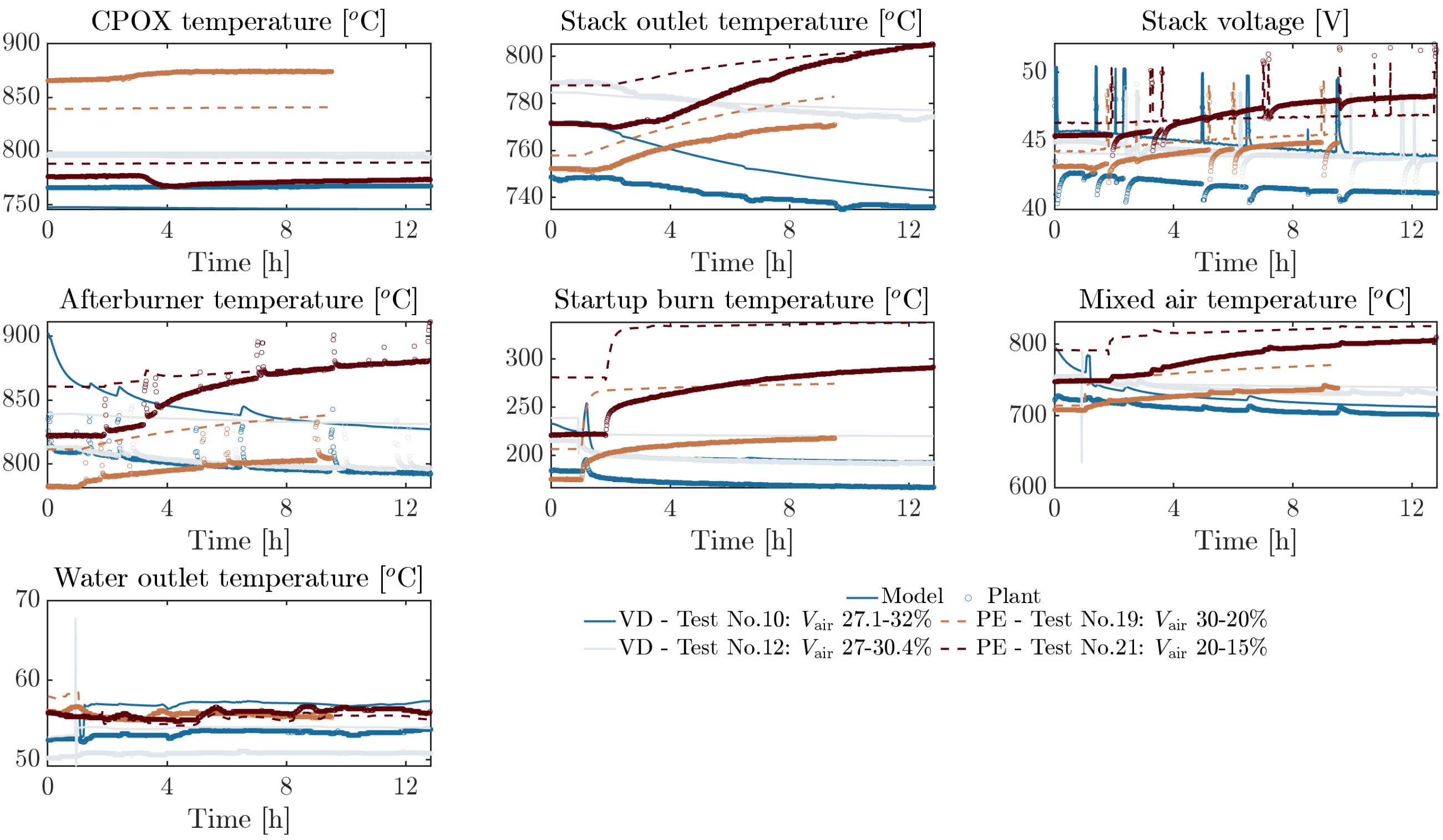

Figure S7. Comparison of dynamic behavior between the model results (lines) and the plant measurements (circles) under different air mixing valve ratios with other input variables unchanged, where the experimantal data applied for parameter estimation is in dash lines and the experimental data for model validation is in solid lines.

Table S2. Stabilization time of different system parameters due to the change of different inputs.

	U_{stack}	T_{cpox}	T_{stack}	T_{burn}	T_{air}
ΔWL_{CH_4}	28 h	0.3 h	28 h	28 h	28 h
$\Delta WL_{\text{air,cpox}}$	2 h	0.3 h	2 h	0.3 h	2 h
$\Delta WL_{\text{air,stack}}$	16 h	-	16 h	16 h	16 h
ΔI	10 h	-	10 h	10 h	10 h
ΔV_{air}	12 h	-	12 h	12 h	12 h

Part D. Model optimum

The model optimum has been first estimated to understand the behavior of the system, and the optimization was formulated as below:

$$\max_{\mathbf{u}} \eta_{el} = \frac{P_{el} - P_{loss}}{n_{in,cpox}^{CH_4} \times LHV_{CH_4}} \text{ or } \eta_{CHP} = \frac{P_{el} - P_{loss} + q_{H_2O} C_{p,H_2O} \times \left(T_{out,H_2O} - T_{in,H_2O}\right)}{n_{in,cpox}^{CH_4} \times LHV_{CH_4}}$$

$$\begin{aligned}
&\text{s.t. System model equations} \\
&P_{el} = P_{el}^{\text{set}}\ [W] \\
&700 \le T_{\text{cpox}} \le 850[°\text{C}] \\
&755 \le T_{\text{stack}} \le 800[°\text{C}] \\
&42 \le U_{\text{stack}} \le 60[\text{V}] \\
&710 \le T_{\text{burn}} \le 900[°\text{C}] \\
&720 \le T_{\text{air}} \le 810[°\text{C}] \\
&\text{FU} \le 79[\%] \\
&\text{AU} \le 25[\%] \\
&50\% \le WL_{CH_4} \le 100\% \\
&60\% \le WL_{\text{air,cpox}} \le 80\% \\
&28.5\% \le WL_{\text{air,stack}} \le 49.5\% \\
&0.1 \le I \le 21[A] \\
&10\% \le V_{\text{air}} \le 30\%
\end{aligned} \tag{S31}$$

Where q_{H_2O} is the water flow rate, C_{p,H_2O} is the heat capacity of water, P_{el}^{set} is the power output target, P_{el} is the model power output, P_{loss} is power consumption from the converter, blowers and pump, as is expressed below:

$$\begin{aligned} P_{loss} = P_{conv} + P_{max,CH_4} \times WL_{CH_4} + P_{max,cpox} \times WL_{air,cpox} + P_{max,pos} \times WL_{air,stack} \\ + P_{max,H_2\text{O}} \times WL_{\text{H}_2\text{O}} \end{aligned} \tag{S32}$$

where P_{conv} is the power loss from the converter, fixed at 8 W, P_{max,CH_4} is the maximal power of the natural gas blower, fixed at 9.6 W, $P_{max,cpox}$ is the maximal power of the CPOX air blower, fixed at 16.32 W, $P_{max,pos}$ is the maximal power of the positrode air blower, fixed at 70 W, $P_{max,H_2\text{O}}$ is the maximal power of the water pump, fixed at 50 W.

The power output target has been set at 840, 800, 760, 720, and 680 W, respectively. The model optimum with different number of manipulated inputs is shown in Table S3-Table S5. When optimizing the electrical efficiency, several model behaviors are noted:

- In different power targets and different inputs manipulated, minimized CPOX air blower workload reduces the power consumption of the blower and maximized FU increase the conversion ratio of the fuel, which hence maximizes electrical efficiency.
- In 5-input case, the upper bound of the stack outlet temperature was activated at 840 and 800 W targets, which then moved to lower bound of air mixing valve ratio, which fully utilized the waste heat to

increase the stack temperature and enhance the performance.

When optimizing the CHP efficiency, several model behaviors are noted:

- In 2-input and 3-input cases, the operating points are highly coherent with those with optimal electrical efficiencies.
- In 5-input case, the natural gas blower workload was maintained at the upper bound.
- In 5-input case, upper bound of CPOX air blower workload was activated, due to the fact that burning more fuel in CPOX can reduce the positrode air blower workload to ensure more heat exchanged in the water HEX and hence increase the CHP efficiency.
- In 5-input case, air mixing valve ratio was kept at its upper bound to bypass as much air as possible, such that more heat will be exchanged in the water HEX, increasing the CHP efficiency.
- In 5-input case, the consequence of high air mixing valve ratio was the activation of the lower bound of mixed air temperature.

Table S3. System optimum with 2 inputs manipulated at different power outputs and different cost functions, where the red bold font indicates the activated lower bound and the blue bold font indicates the activated upper bound.

	Power [W]	η_{el} [%]	η_{CHP} [%]	WL_{CH_4} [%]	$WL_{\mathrm{air,cpox}}$ [%]	$WL_{\mathrm{air,stack}}$ [%]	I [A]	V_{air} [%]	FU [%]	AU [%]	U_{stack} [V]	T_{cpox} [°C]	T_{stack} [°C]	T_{burn} [°C]	T_{air} [°C]
max η_{el}	840	32.7	70.8	85.2	63	31.2	19.7	18.0	**79**	10	42.7	791.8	791.3	809.2	767.8
	800	32.0	70.8	82.2	63	31.2	18.7	18.0	**79**	10	42.7	803.4	785.6	801.4	760.4
	760	31.3	70.8	79.3	63	31.2	17.8	18.0	**79**	9	42.8	815.1	779.8	793.6	752.9
	720	30.5	70.7	76.3	63	31.2	16.8	18.0	**79**	9	42.9	826.9	773.9	785.6	745.4
	680	29.7	70.7	73.4	63	31.2	15.8	18.0	**79**	8	43.0	838.8	768.0	777.6	737.7
Max η_{CHP}	840	32.7	70.8	85.2	63	31.2	19.7	18.0	**79**	10	42.7	791.8	791.3	809.2	767.8
	800	32.0	70.8	82.2	63	31.2	18.7	18.0	**79**	10	42.7	803.4	785.6	801.4	760.4
	760	31.3	70.8	79.3	63	31.2	17.8	18.0	**79**	9	42.8	815.1	779.8	793.6	752.9
	720	30.5	70.7	76.3	63	31.2	16.8	18.0	**79**	9	42.9	826.9	773.9	785.6	745.4
	680	29.7	70.7	73.4	63	31.2	15.8	18.0	**79**	8	43.0	838.8	768.0	777.6	737.7

Table S4. System optimum with 3 inputs manipulated at different power outputs and different cost functions, where the red bold font indicates the activated lower bound and the blue bold font indicates the activated upper bound.

	Power [W]	η_{el} [%]	η_{CHP} [%]	WL_{CH_4} [%]	$WL_{\mathrm{air,cpox}}$ [%]	$WL_{\mathrm{air,stack}}$ [%]	I [A]	V_{air} [%]	FU [%]	AU [%]	U_{stack} [V]	T_{cpox} [°C]	T_{stack} [°C]	T_{burn} [°C]	T_{air} [°C]
max η_{el}	840	33.0	70.8	84.4	**60**	31.2	19.8	18.0	**79**	11	42.3	777.2	785.3	803.9	762.8
	800	32.3	70.8	81.5	**60**	31.2	18.9	18.0	**79**	10	42.4	788.9	779.5	796.1	755.3
	760	31.6	70.8	78.5	**60**	31.2	17.9	18.0	**79**	9	42.4	800.7	773.7	788.1	747.7
	720	30.8	70.8	75.5	**60**	31.2	16.9	18.0	**79**	9	42.5	812.6	767.7	780.0	740.0
	680	30.0	70.8	72.6	**60**	31.2	16.0	18.0	**79**	8	42.6	824.5	761.7	771.8	732.3
Max η_{CHP}	840	32.8	70.8	85.1	62.8	31.2	19.7	18.0	**79**	10	42.6	791.0	791.0	809.0	767.5
	800	32.2	70.8	81.9	61.5	31.2	18.8	18.0	**79**	10	42.6	796.4	782.6	798.8	757.9
	760	31.5	70.8	78.7	60.8	31.2	17.9	18.0	**79**	9	42.5	804.5	775.3	789.5	749.1
	720	30.8	70.8	75.5	**60**	31.2	16.9	18.0	**79**	9	42.5	812.6	767.7	780.0	740.0
	680	30.0	70.8	72.6	**60**	31.2	16.0	18.0	**79**	8	42.6	824.5	761.7	771.8	732.3

Table S5. System optimum with 5 inputs manipulated at different power outputs and different cost functions, where the red bold font indicates the activated lower bound and the blue bold font indicates the activated upper bound.

	Power [W]	η_{el} [%]	η_{CHP} [%]	WL_{CH_4} [%]	$WL_{\mathrm{air,cpox}}$ [%]	$WL_{\mathrm{air,stack}}$ [%]	I [A]	V_{air} [%]	FU [%]	AU [%]	U_{stack} [V]	T_{cpox} [°C]	T_{stack} [°C]	T_{burn} [°C]	T_{air} [°C]
m	840	33.5	66.5	83.2	**60**	**28.5**	19.4	13.7	**79**	11	43.2	783.3	**800.0**	820.6	792.4

	800	33.0	64.3	79.9	**60**	**28.5**	18.3	11.4	**79**	10	43.6	796.7	**800.0**	819.7	796.3
	760	32.3	62.9	76.8	**60**	**28.5**	17.3	**10.0**	**79**	10	43.8	809.4	797.9	816.4	795.6
	720	31.5	62.7	73.9	**60**	**28.5**	16.3	**10.0**	**79**	9	43.9	821.0	792.1	808.4	787.8
	680	30.7	60.7	71.0	**60**	**28.5**	15.4	**10.0**	**79**	9	44.0	832.6	786.2	800.4	780.0
Max η_{CHP}	840	28.0	81.6	**100**	**80**	41.9	19.9	**30.0**	70	9	42.4	822.0	776.5	815.2	**720.0**
	800	26.5	81.4	**100**	**80**	43.7	18.7	**30.0**	66	8	43.0	821.6	771.0	818.8	**720.0**
	760	25.0	81.3	**100**	**80**	45.5	17.4	**30.0**	62	7	43.6	821.3	765.8	822.2	**720.0**
	720	23.5	81.1	**100**	**80**	47.3	16.3	**30.0**	57	7	44.2	821.0	760.8	825.6	**720.0**
	680	22.0	80.9	**99.9**	**80**	49.1	15.2	**30.0**	54	6	44.9	820.9	756.1	828.6	**720.0**

Part E. Results of CA RTO algorithm

All the cases with electrical efficiency as the cost function applied asynchronous control strategy, while only the last 5-input test with CHP efficiency as cost function applied synchronous control strategy. The modified optimization problem based on the model can be formulated as below:

$$\max_{\mathbf{u}} \eta_{el} = \frac{P_{el} - P_{loss}}{n_{in,cpox}^{CH_4} \times LHV_{CH_4}} \text{ or } \eta_{CHP} = \frac{P_{el} - P_{loss} + q_{H_2O} C_{p,H_2O} \times \left(T_{out,H_2O} - T_{in,H_2O}\right)}{n_{in,cpox}^{CH_4} \times LHV_{CH_4}}$$

$$\begin{aligned}
&\text{s.t. System model equations}\\
&P_{el} = P_{el}^{\text{set}} + \hat{\epsilon}_k^{P_{el}} [W]\\
&700 + \hat{\epsilon}_k^{T_{cpox}} \leq T_{\text{cpox}} \leq 850 + \hat{\epsilon}_k^{T_{cpox}} [°\text{C}]\\
&755 + \hat{\epsilon}_k^{T_{stack}} \leq T_{\text{stack}} \leq 800 + \hat{\epsilon}_k^{T_{stack}} [°\text{C}]\\
&42 + \hat{\epsilon}_k^{U_{stack}} \leq U_{\text{stack}} \leq 60 + \hat{\epsilon}_k^{U_{stack}} [\text{V}]\\
&710 + \hat{\epsilon}_k^{T_{burn}} \leq T_{\text{burn}} \leq 900 + \hat{\epsilon}_k^{T_{burn}} [°\text{C}]\\
&720 + \hat{\epsilon}_k^{T_{air}} \leq T_{\text{air}} \leq 810 + \hat{\epsilon}_k^{T_{air}} [°\text{C}]\\
&\text{FU} \leq 79 [\%]\\
&\text{AU} \leq 25 [\%]\\
&50\% \leq WL_{CH_4} \leq 100\%\\
&60\% \leq WL_{\text{air,cpox}} \leq 80\%\\
&28.5\% \leq WL_{\text{air,stack}} \leq 49.5\%\\
&0.1 \leq I \leq 21 [A]\\
&10\% \leq V_{\text{air}} \leq 30\%
\end{aligned} \tag{S33}$$

Table S6. System limitations, where the violation of temperature and stack voltage will trigger emergency shutdown and the OCV indicated by the probe cell determines the potential risk of carbon deposition.

	T_{cpox} [°C]	T_{stack} [°C]	T_{air} [°C]	T_{burn} [°C]	U_{stack} [V]	U_{OCV} [V]	WL_{CH_4} [%]	$WL_{air,cpox}$ [%]	$WL_{air,st}$ [%]	C [A]	V_{air} [%]
**Max.**	1100	850	850	700	60	1.07	100	80	50	21	30
**Min.**	390	735	680	-	40	-	50	60	28	-	10

1. Electrical efficiency as the cost function

1.1. 2 inputs case

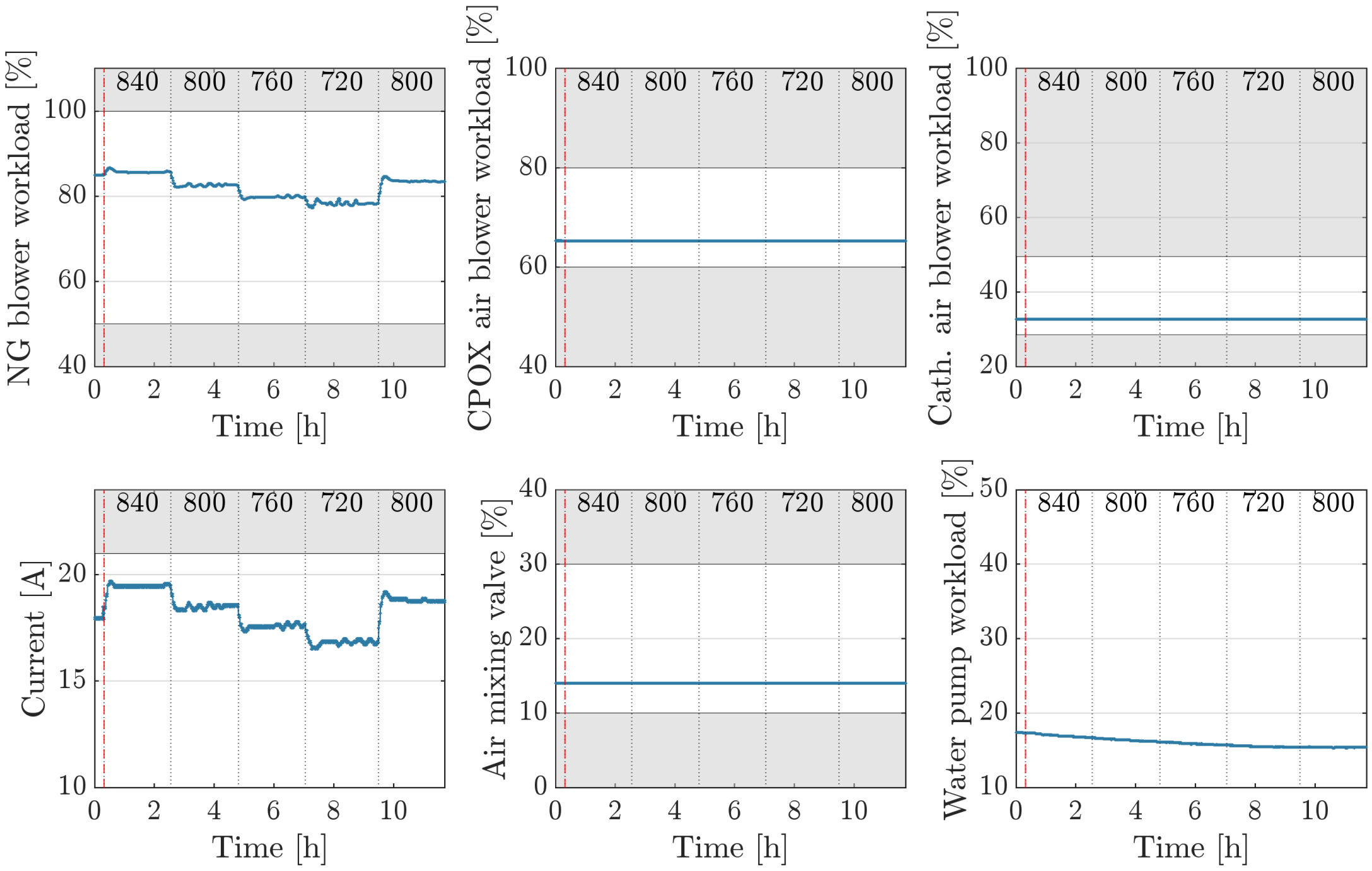

Figure S8. 2-input case for max. electrical efficiency: System inputs during the CA RTO test with natural gas blower workload and current manipulated, where the red dash line indicates the activation of CA RTO algorithm and the numbers segmented by dotted lines denote the power target in W.

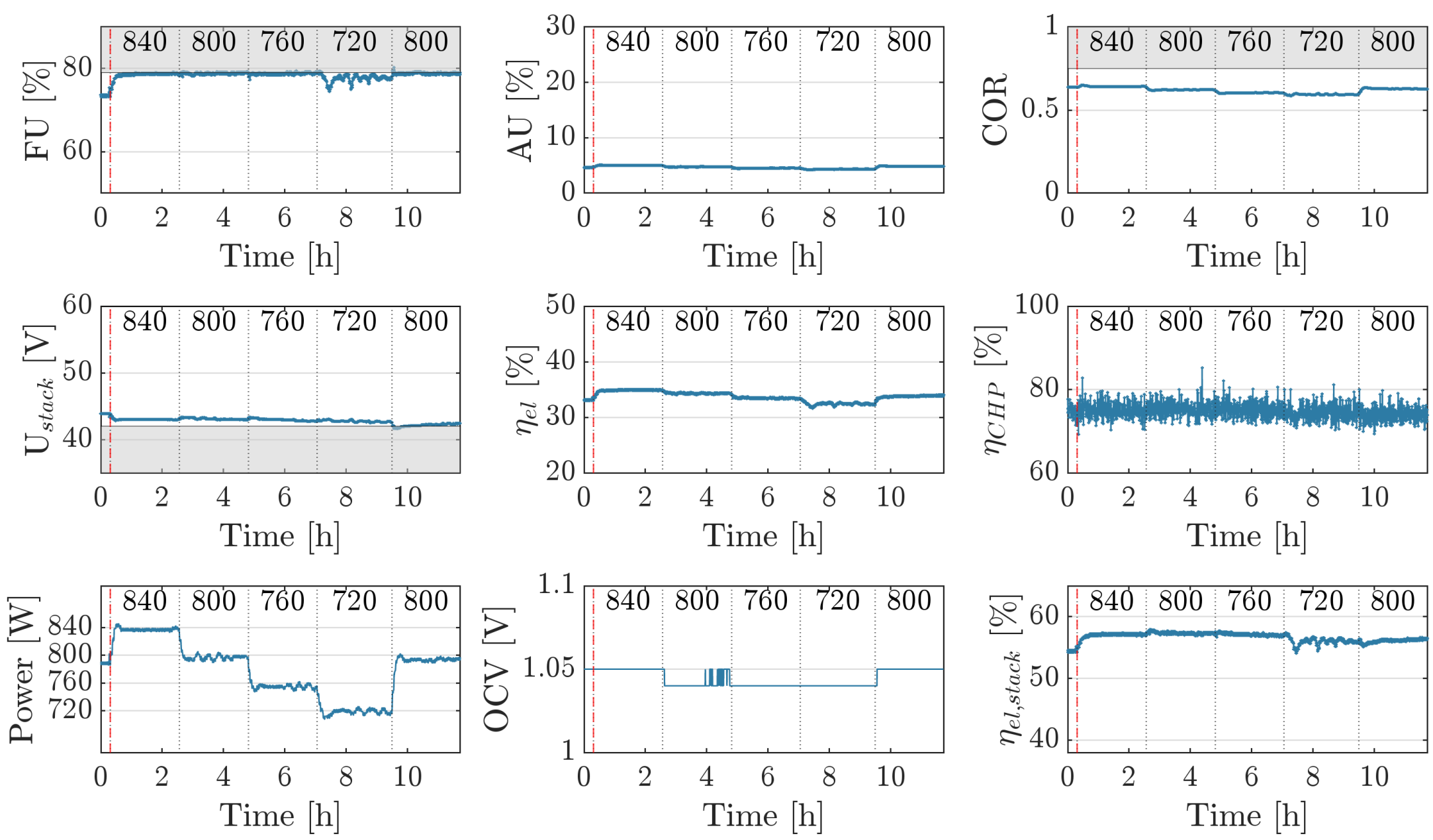

Figure S9. 2-input case for max. electrical efficiency: System key indicators during the CA RTO test with natural gas blower workload and current manipulated, where the red dash line indicates the activation of CA RTO algorithm and the numbers segmented by dotted lines denote the power target in W.

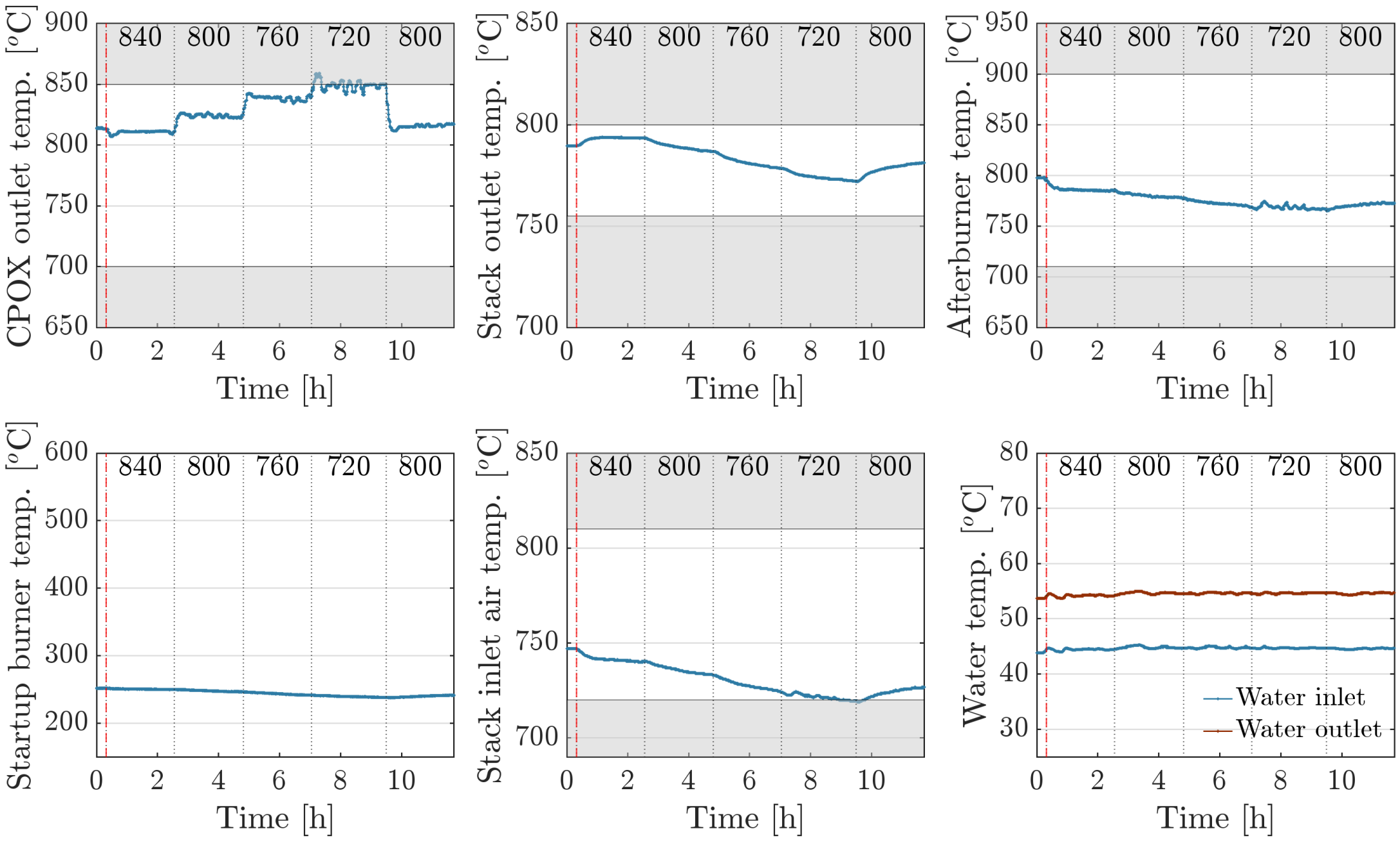

Figure S10. 2-input case for max. electrical efficiency: BoP temperatures during the CA RTO test with natural gas blower workload and current manipulated, where the red dash line indicates the activation of CA RTO algorithm and the numbers segmented by dotted lines denote the power target in W.

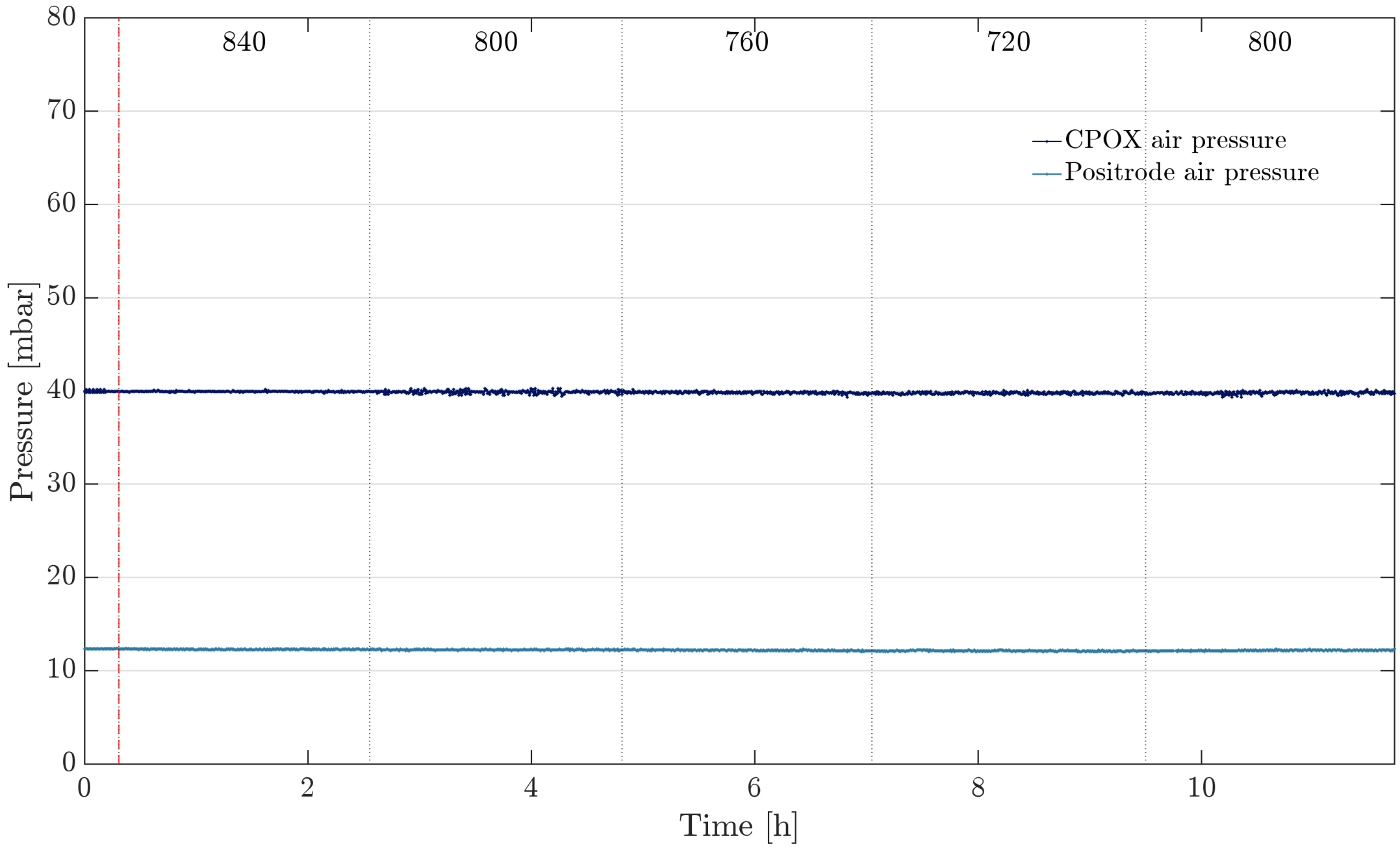

Figure S11. 2-input case for max. electrical efficiency: Outlet pressures of the blowers during the CA RTO test with natural gas blower workload and current manipulated, where the red dash line indicates the activation of CA RTO algorithm and the numbers segmented by dotted lines denote the power target in W.

1.2. 3 inputs case

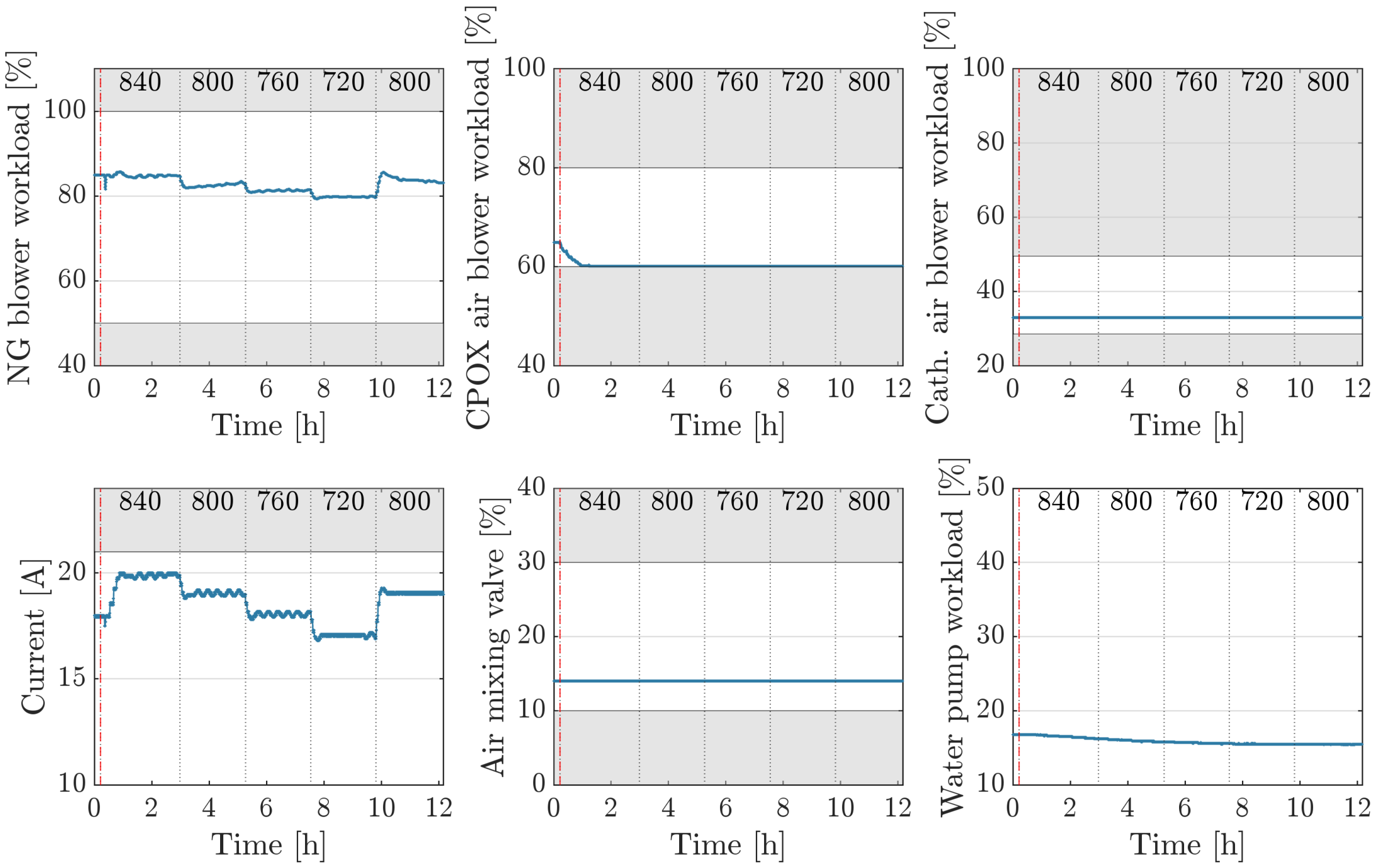

Figure S12. 3-input case for max. electrical efficiency: System inputs during the CA RTO test with natural gas blower workload, CPOX air blower workload and current manipulated, where the red dash line indicates the activation of CA RTO algorithm and the numbers segmented by dotted lines denote the power target in W.

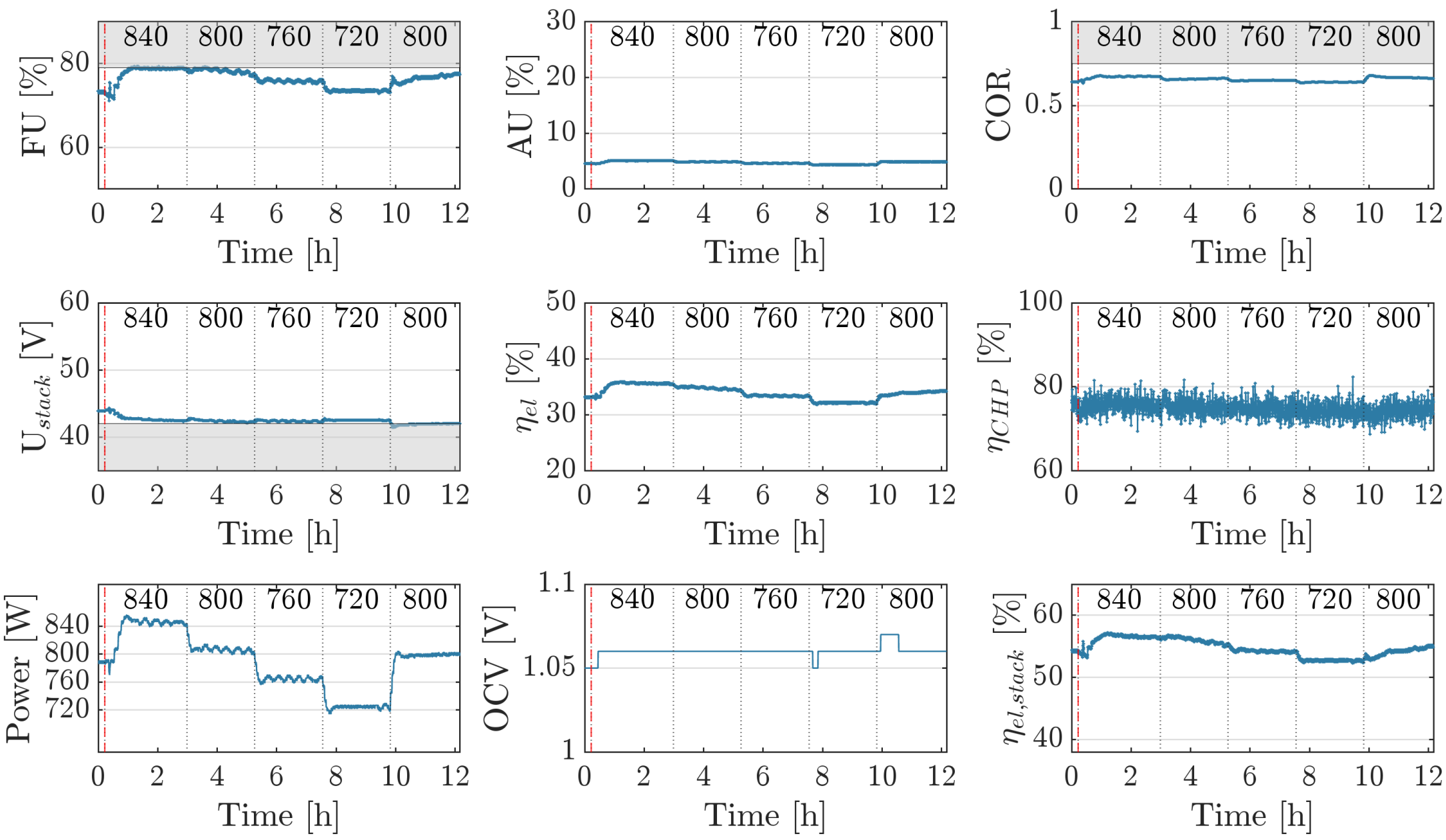

Figure S13. 3-input case for max. electrical efficiency: System key indicators during the CA RTO test with natural gas blower workload, CPOX air blower workload and current manipulated, where the red dash line indicates the activation of CA RTO algorithm and the numbers segmented by dotted lines denote the power target in W.

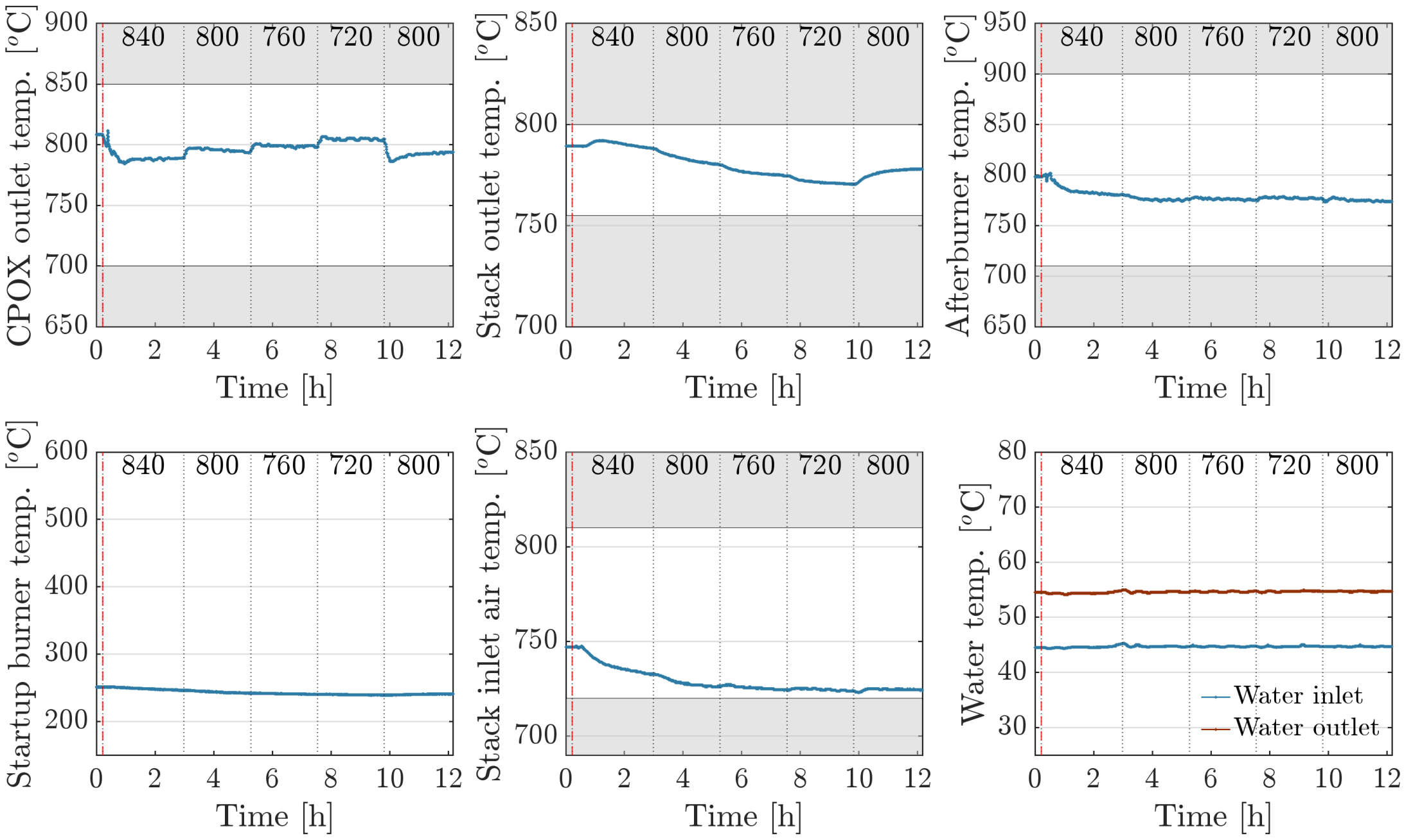

Figure S14. 3-input case for max. electrical efficiency: BoP temperatures during the CA RTO test with natural gas blower workload, CPOX air blower workload and current manipulated, where the red dash line indicates the activation of CA RTO algorithm and the numbers segmented by dotted lines denote the power target in W.

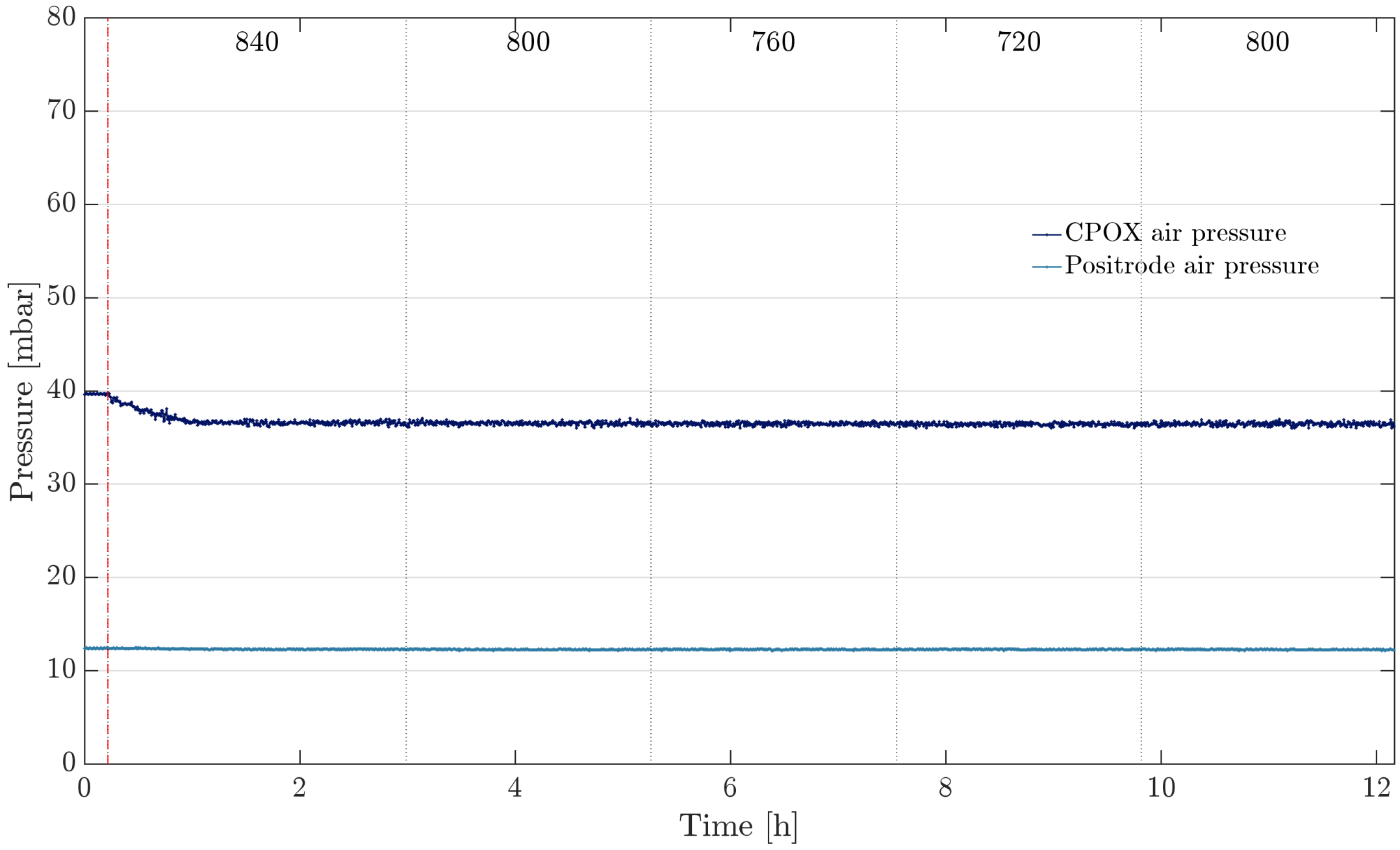

Figure S15. 3-input case for max. electrical efficiency: Outlet pressures of the blowers during the CA RTO test with natural gas blower workload, CPOX air blower workload and current manipulated, where the red dash line indicates the activation of CA RTO algorithm and the numbers segmented by dotted lines denote the power target in W.

1.3. 5 inputs case

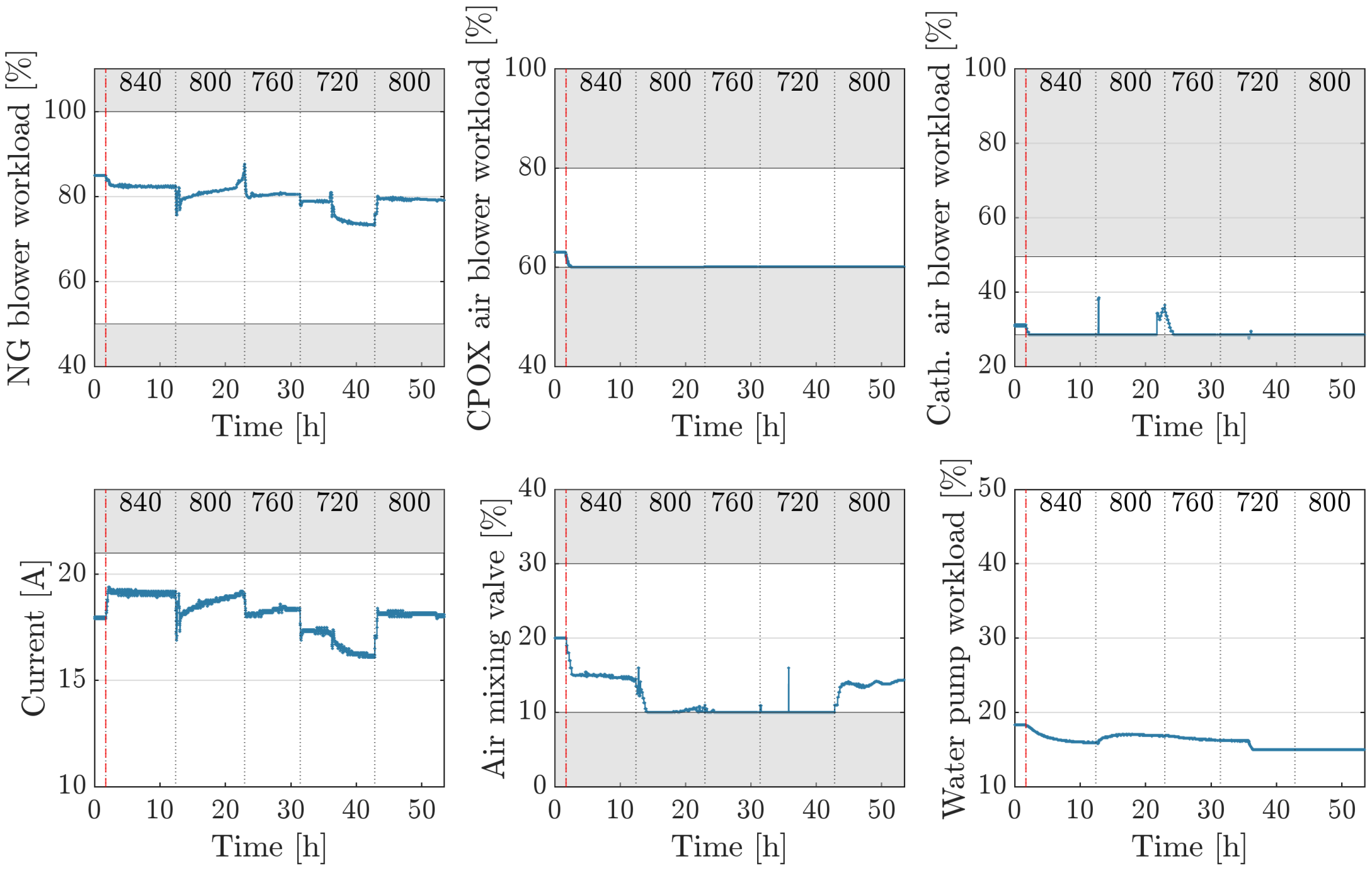

Figure S16. 5-input case for max. electrical efficiency: System inputs during the CA RTO test with natural gas blower workload, CPOX air blower workload, positrode air blower workload, current, and air mixing valve ratio manipulated, where the red dash line indicates the activation of CA RTO algorithm and the numbers segmented by dotted lines denote the power target in W.

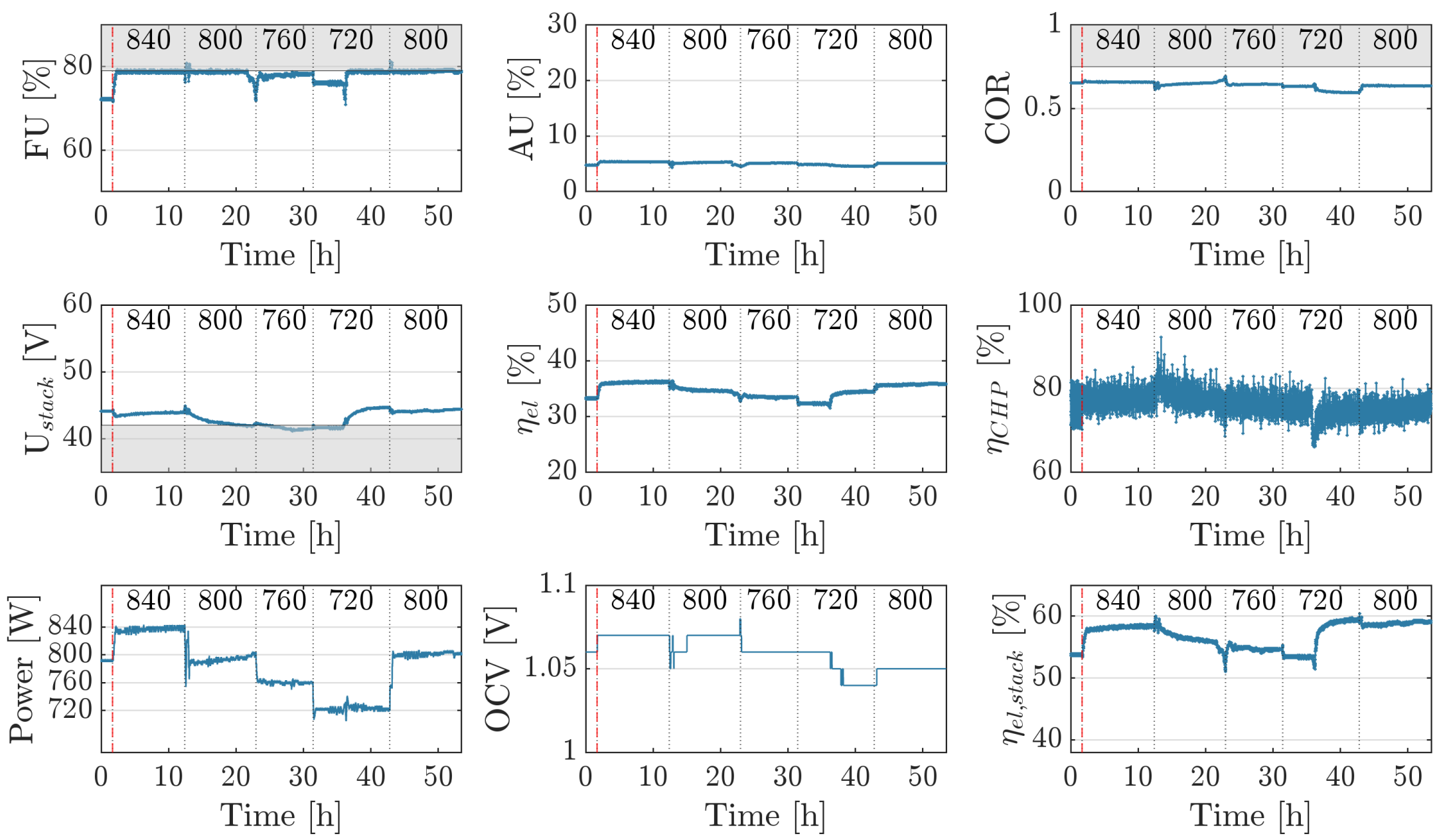

Figure S17. 5-input case for max. electrical efficiency: System key indicators during the CA RTO test with natural gas blower workload, CPOX air blower workload, positrode air blower workload, current, and air mixing valve ratio manipulated, where the red dash line indicates the activation of CA RTO algorithm and the numbers segmented by dotted lines denote the power target in W.

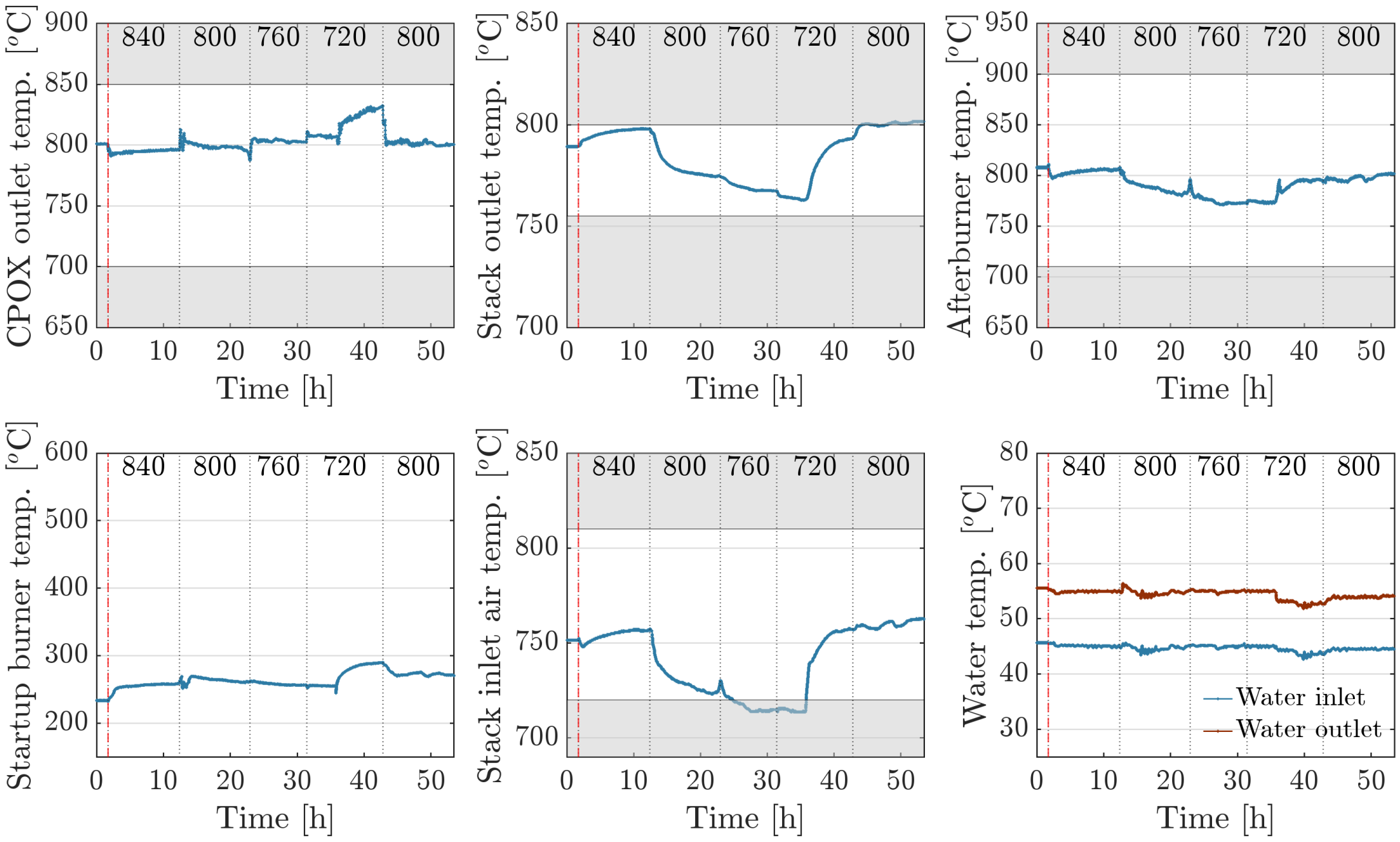

Figure S18. 5-input case for max. electrical efficiency: BoP temperatures during the CA RTO test with natural gas blower workload, CPOX air blower workload, positrode air blower workload, current, and air mixing valve ratio manipulated, where the red dash line indicates the activation of CA RTO algorithm and the numbers segmented by dotted lines denote the power target in W.

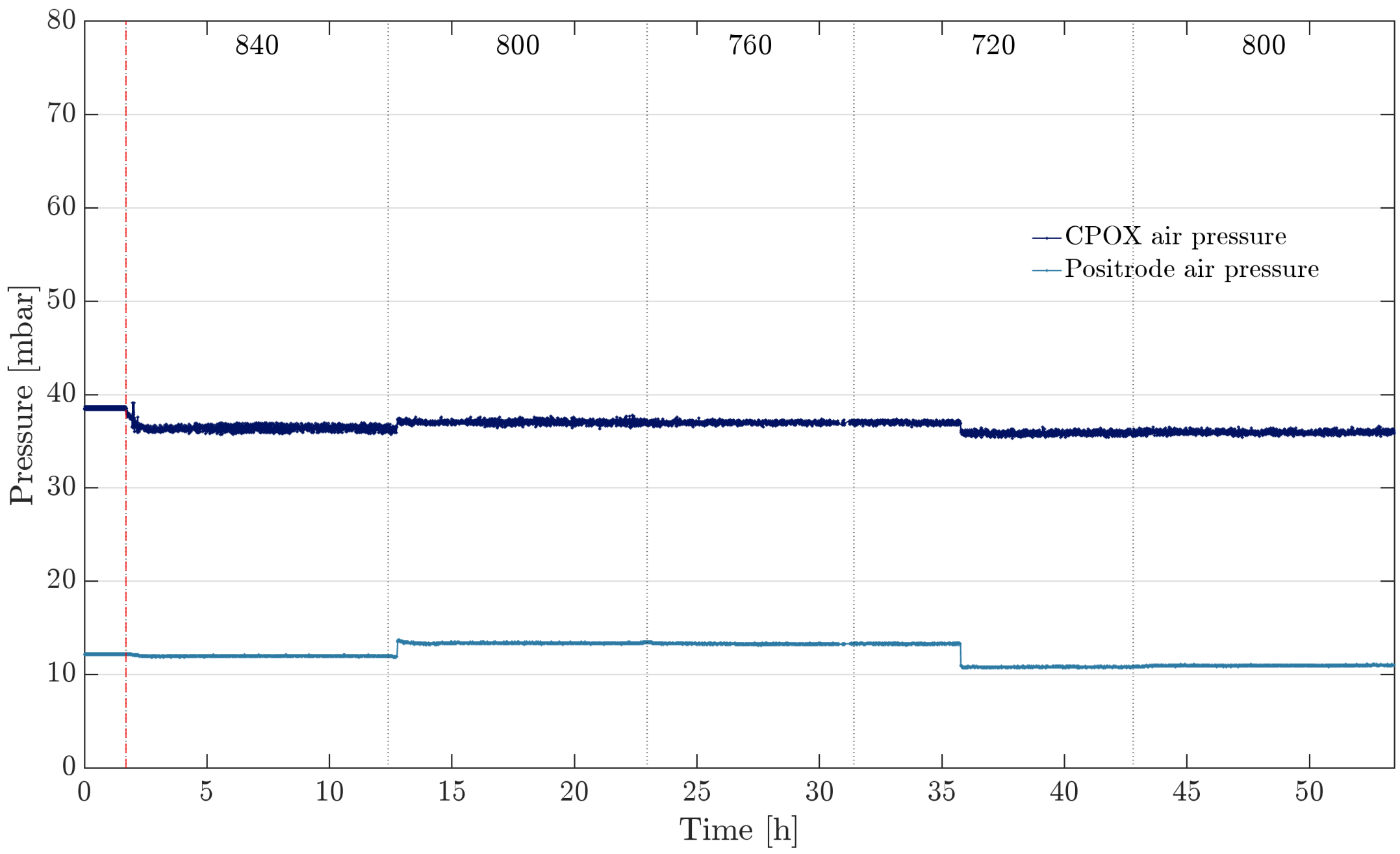

Figure S19. 5-input case for max. electrical efficiency: Outlet pressures of the blowers during the CA RTO test with natural gas blower workload, CPOX air blower workload, positrode air blower workload, current, and air mixing valve ratio manipulated, where the red dash line indicates the activation of CA RTO algorithm and the numbers segmented by dotted lines denote the power target in W.

1.4. 5-input case results with CPOX air blower lower bound at 50%

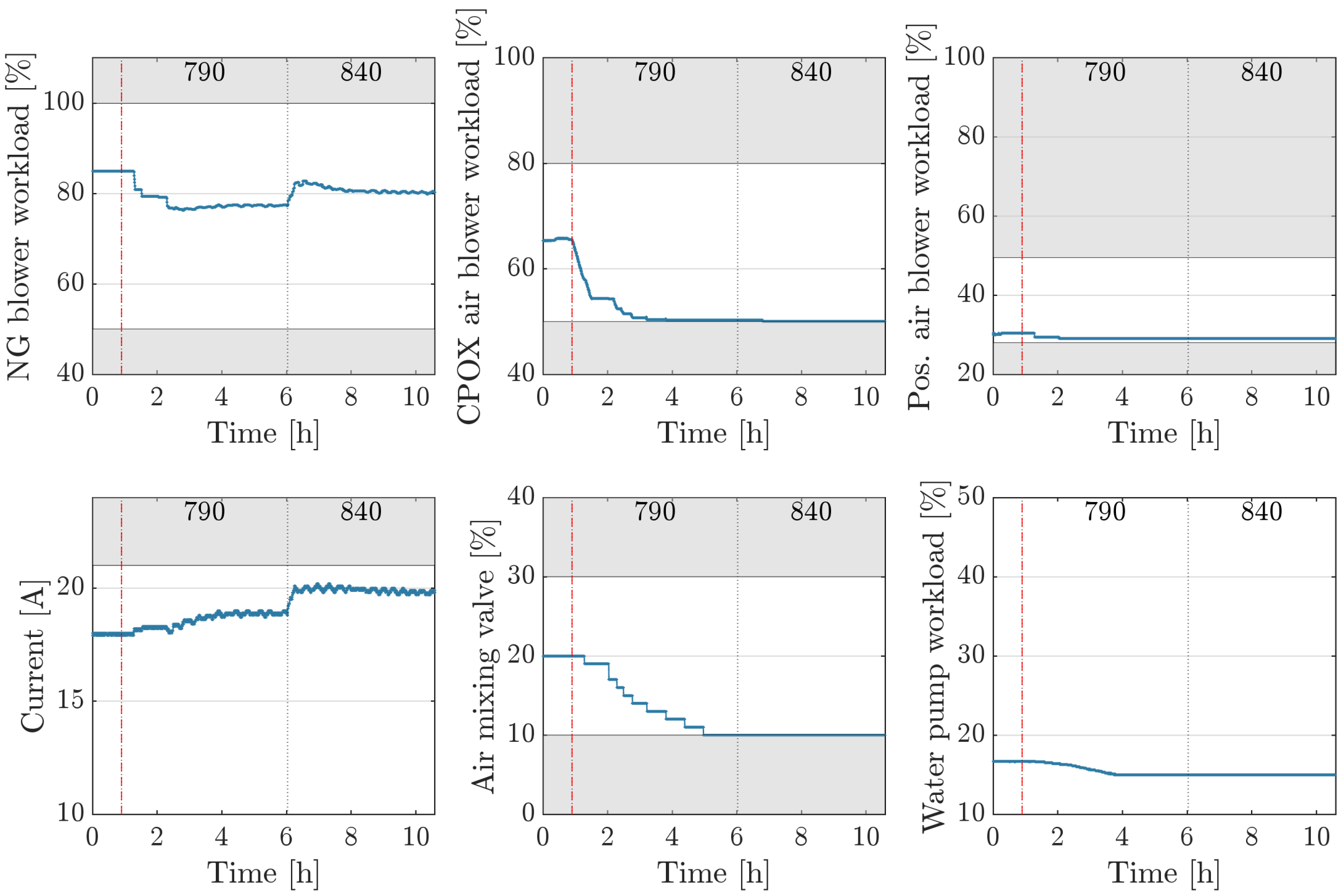

Figure S20. 5-input case for max. electrical efficiency with CPOX air blower lower bound at 50%: System inputs during the CA RTO test with natural gas blower workload, CPOX air blower workload, positrode air blower workload, current, and air mixing valve ratio manipulated, where the red dash line indicates the activation of CA RTO algorithm and the numbers segmented by dotted lines denote the power target in W.

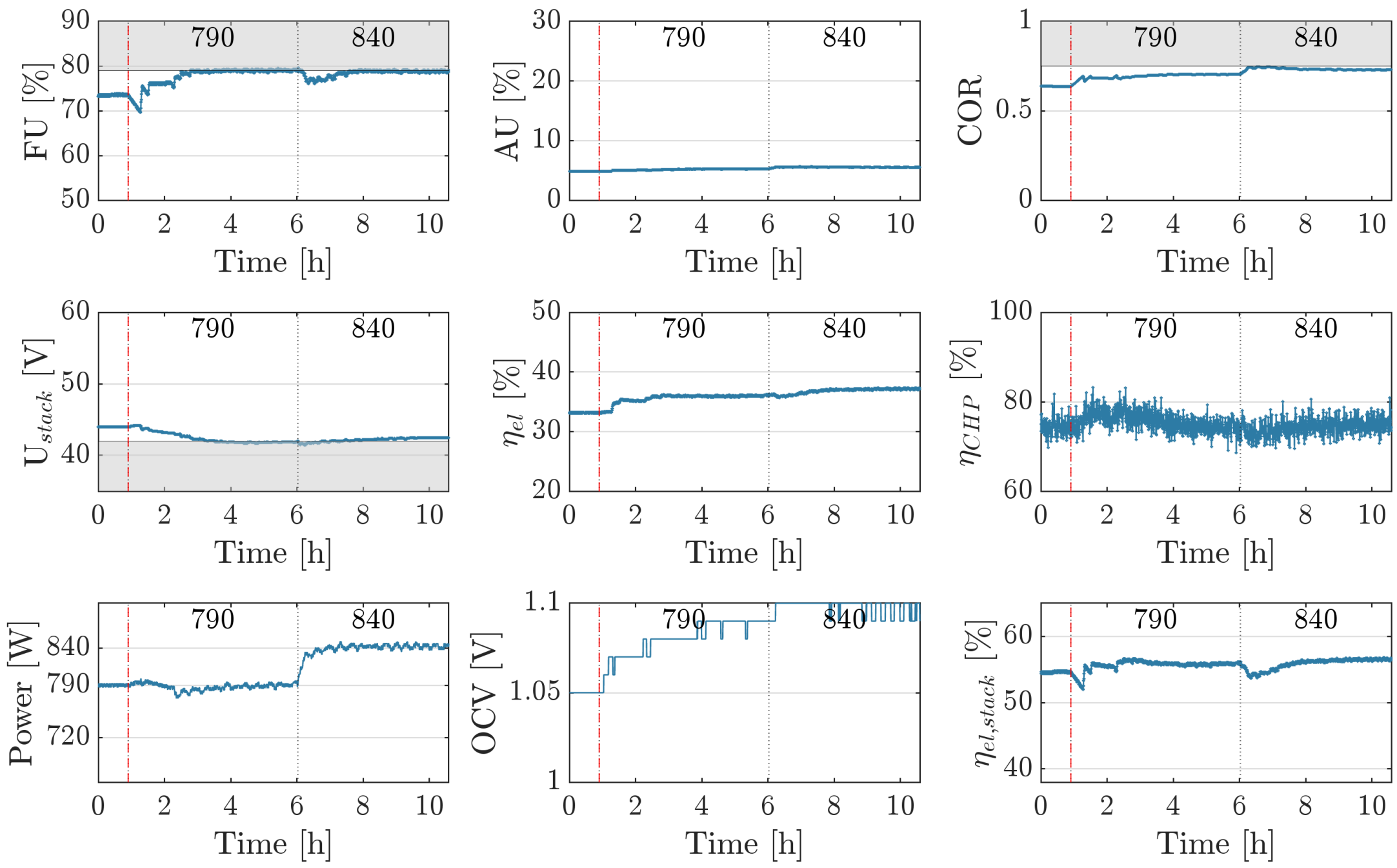

Figure S21. 5-input case for max. electrical efficiency with CPOX air blower lower bound at 50%: System key indicators during the CA RTO test with natural gas blower workload, CPOX air blower workload, positrode air blower workload, current, and air mixing valve ratio manipulated, where the red dash line indicates the activation of CA RTO algorithm and the numbers segmented by dotted lines denote the power target in W.

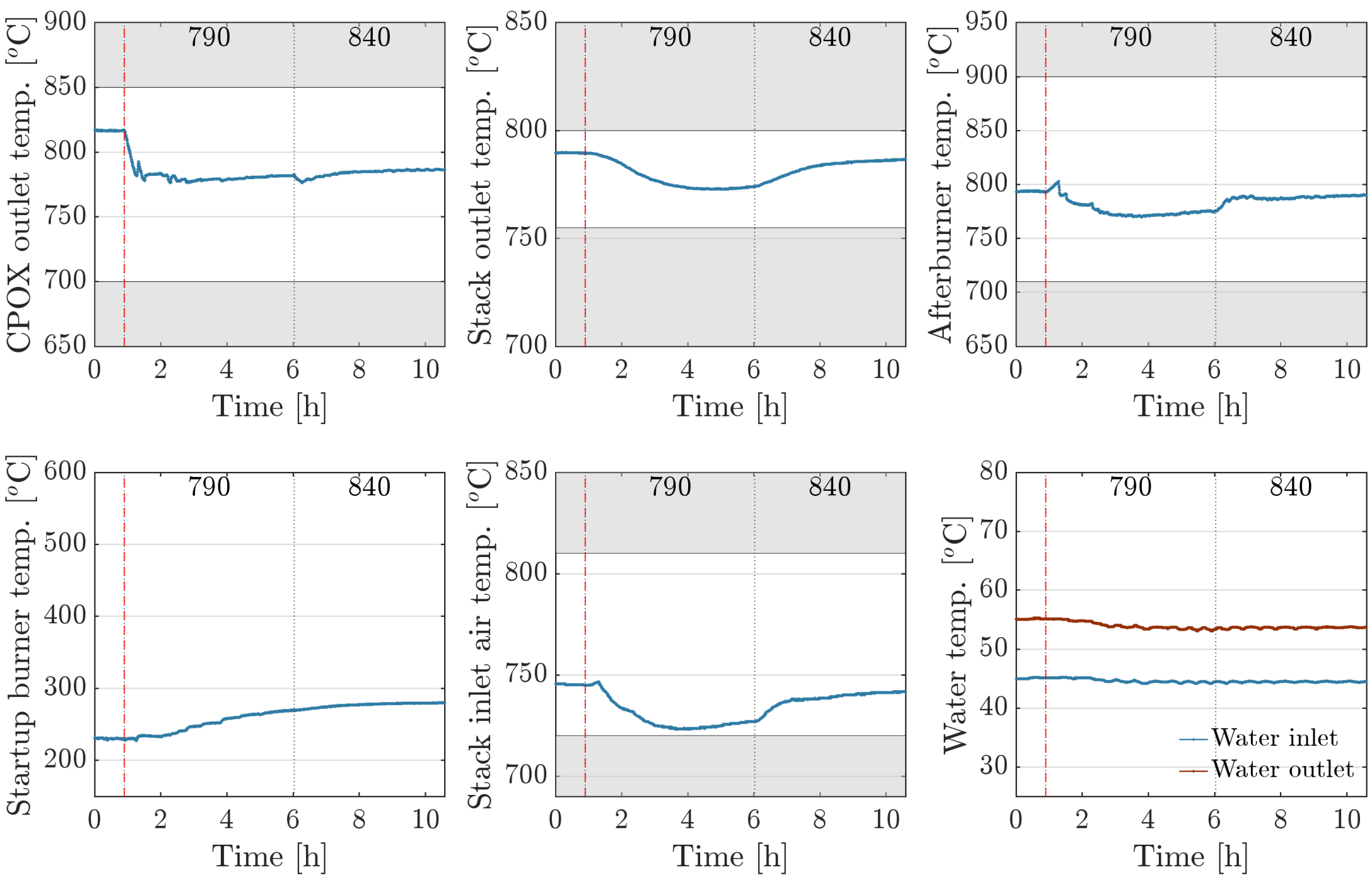

Figure S22. 5-input case for max. electrical efficiency with CPOX air blower lower bound at 50%: BoP temperatures during the CA RTO test with natural gas blower workload, CPOX air blower workload, positrode air blower workload, current, and air mixing valve ratio manipulated, where the red dash line indicates the activation of CA RTO algorithm and the numbers segmented by dotted lines denote the power target in W.

1.5. Evidence of plant-model mismatch and impact from 23-hour self-check

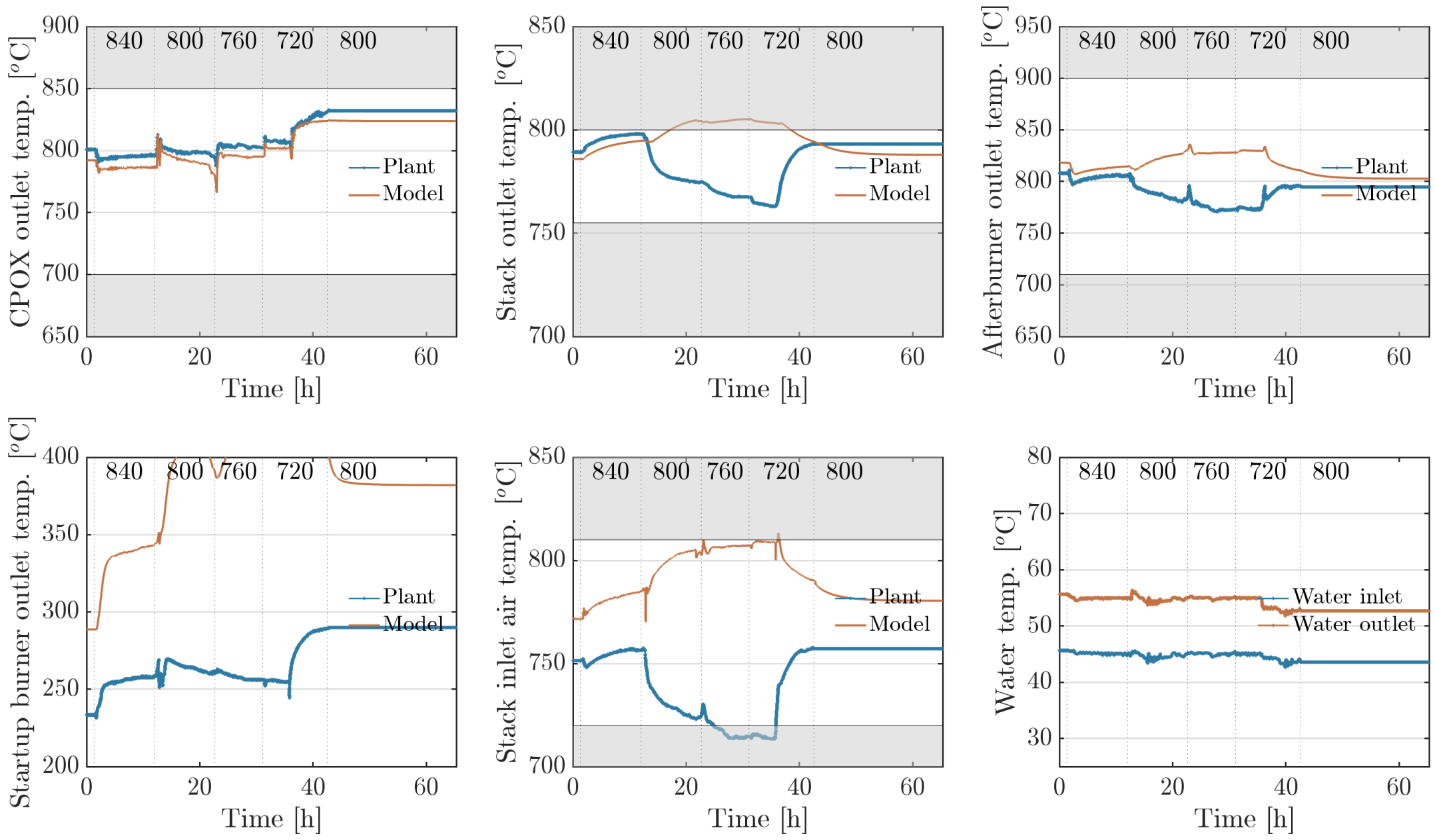

Figure S23. Plant-model mismatch indicated by the temperatures during the CA RTO test with five manipulated inputs, natural gas blower workload, CPOX air blower workload, positrode air blower workload, current, and air mixing valve ratio, where the numbers segmented by dotted lines denote the power target in W.

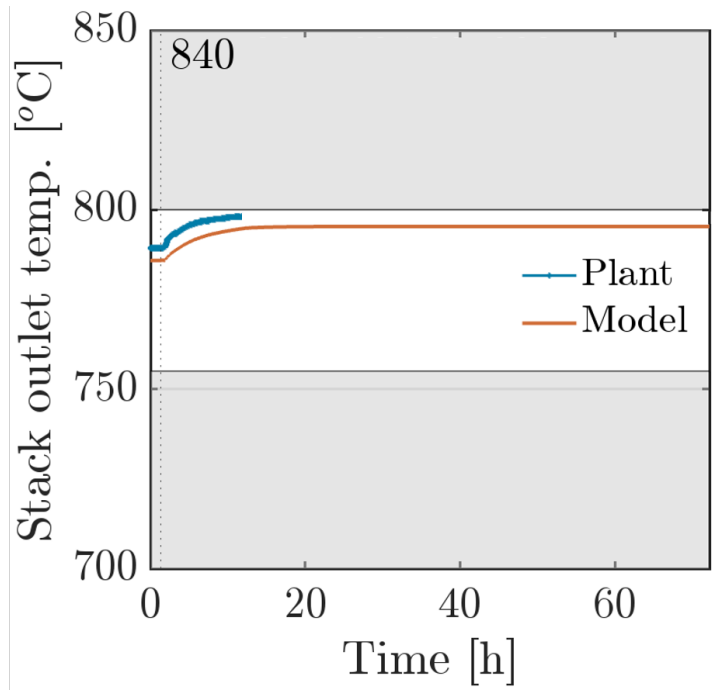

Figure S24. Plant-model mismatch of stack outlet temperature during the CA RTO test at 840 W with five manipulated inputs, natural gas blower workload, CPOX air blower workload, positrode air blower workload, current and air mixing valve ratio, where the system did not reach steady state and the constraint modifier of stack outlet temperature was 3.8 °C with modeled temperature stabilized at 795.3 °C. This indicated the actual temperature would stabilize at around 800 799.1 °C.

2. CHP efficiency as the cost function

2.1. 2 inputs case

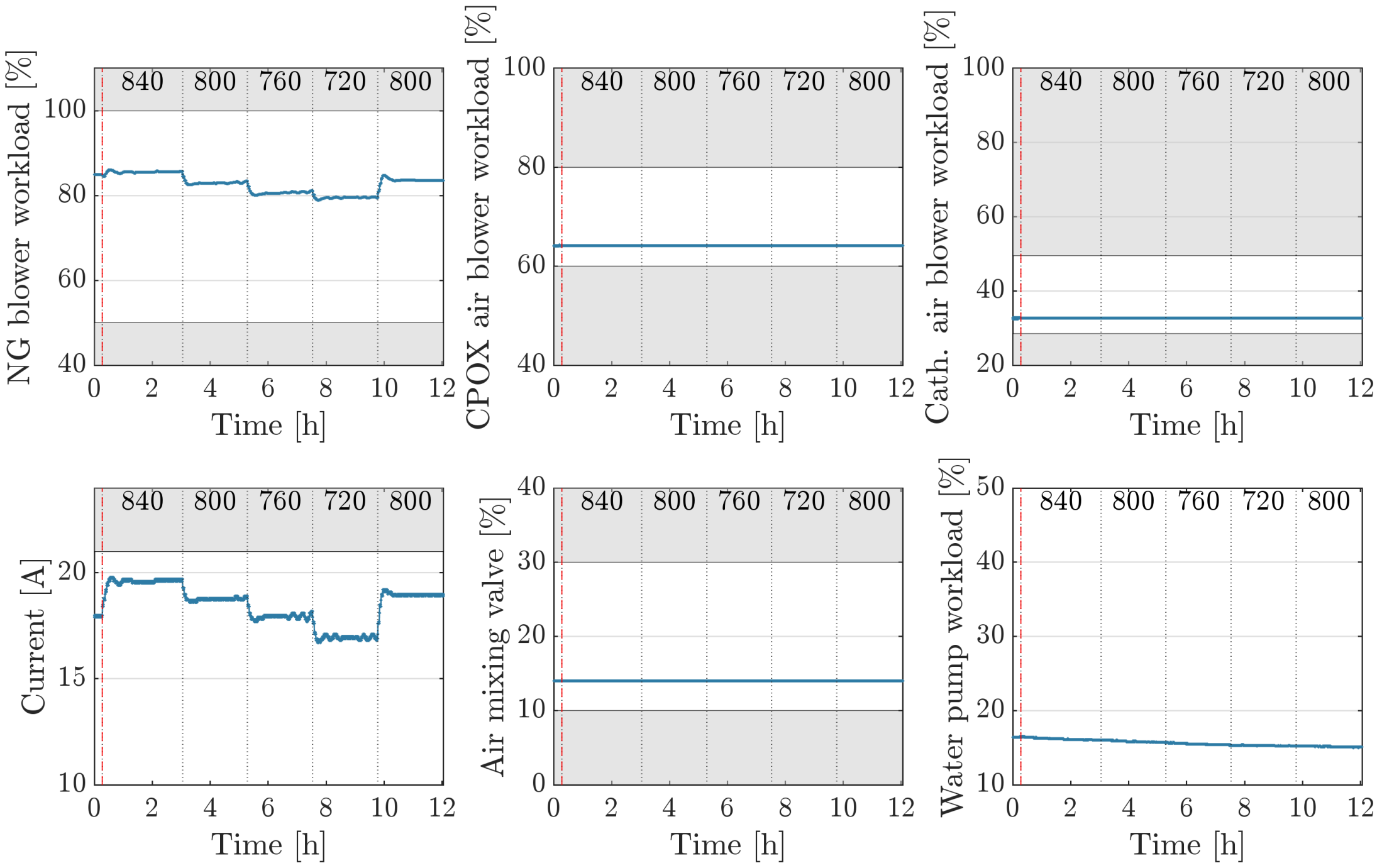

Figure S25. 2-input case for max. CHP efficiency: System inputs during the CA RTO test with natural gas blower workload and current manipulated, where the red dash line indicates the activation of CA RTO algorithm and the numbers segmented by dotted lines denote the power target in W.

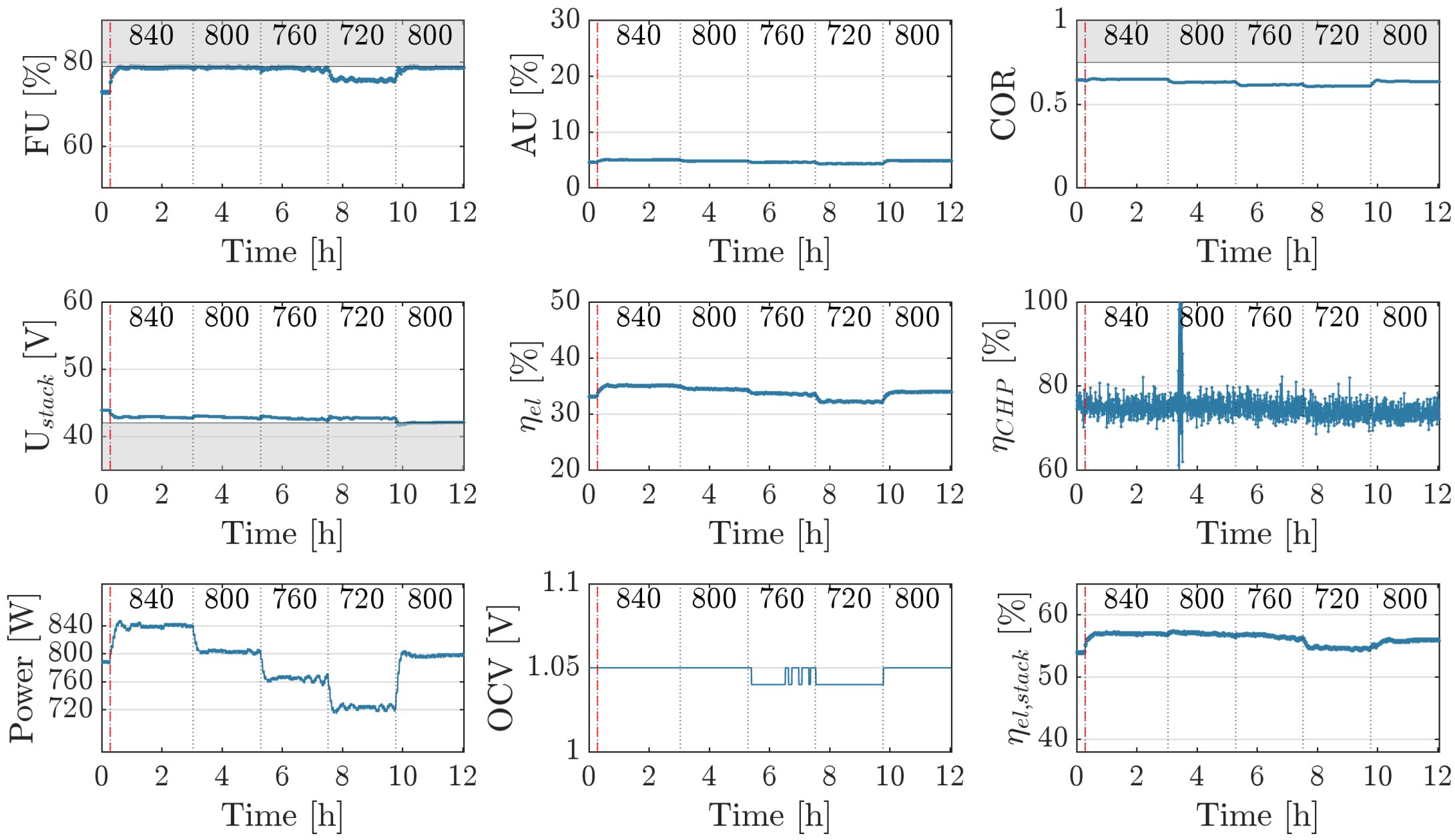

Figure S26. 2-input case for max. CHP efficiency: System key indicators during the CA RTO test with natural gas blower workload and current manipulated, where the red dash line indicates the activation of CA RTO algorithm and the numbers segmented by dotted lines denote the power target in W.

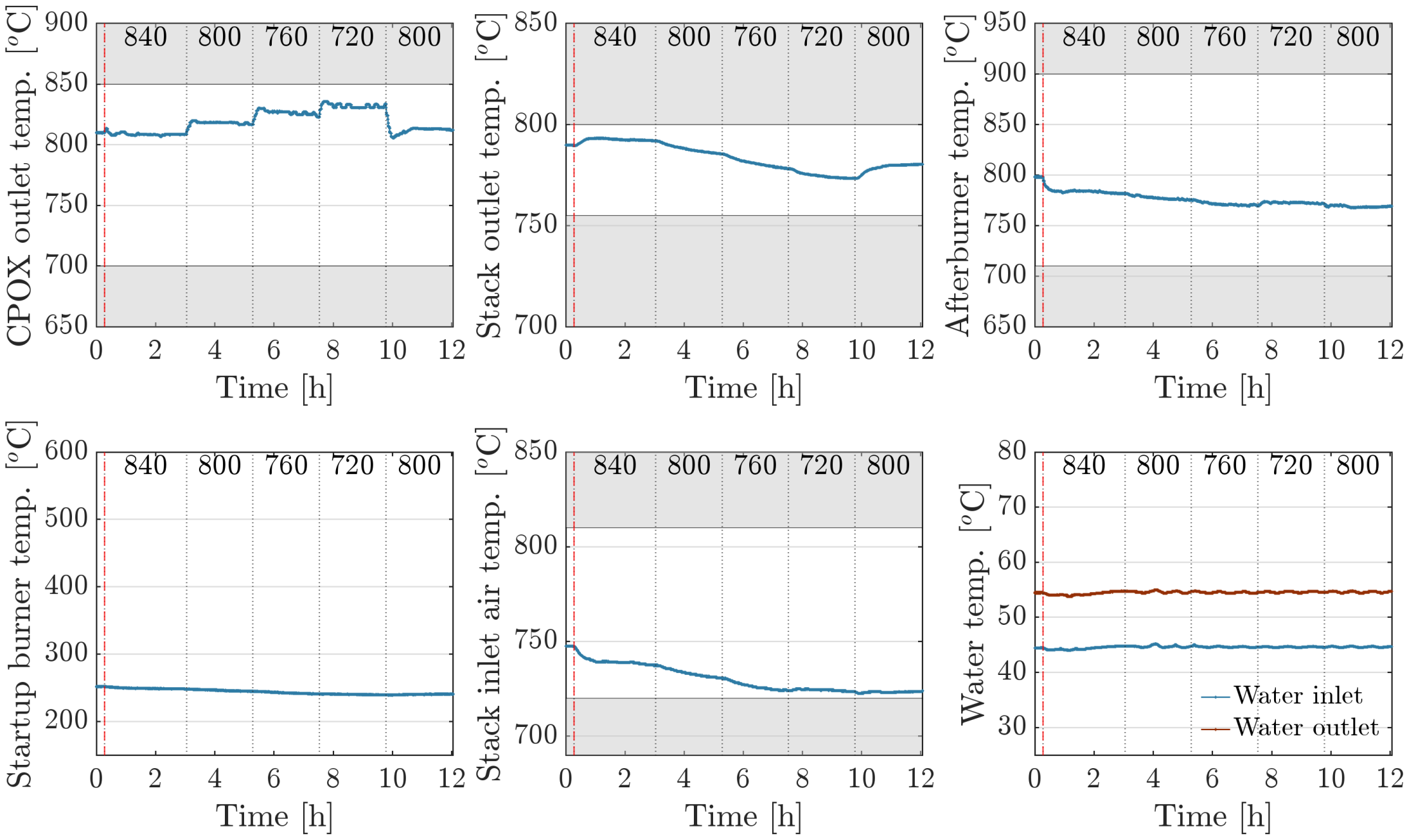

Figure S27. 2-input case for max. CHP efficiency: BoP temperatures during the CA RTO test with natural gas blower workload and current manipulated, where the red dash line indicates the activation of CA RTO algorithm and the numbers segmented by dotted lines denote the power target in W.

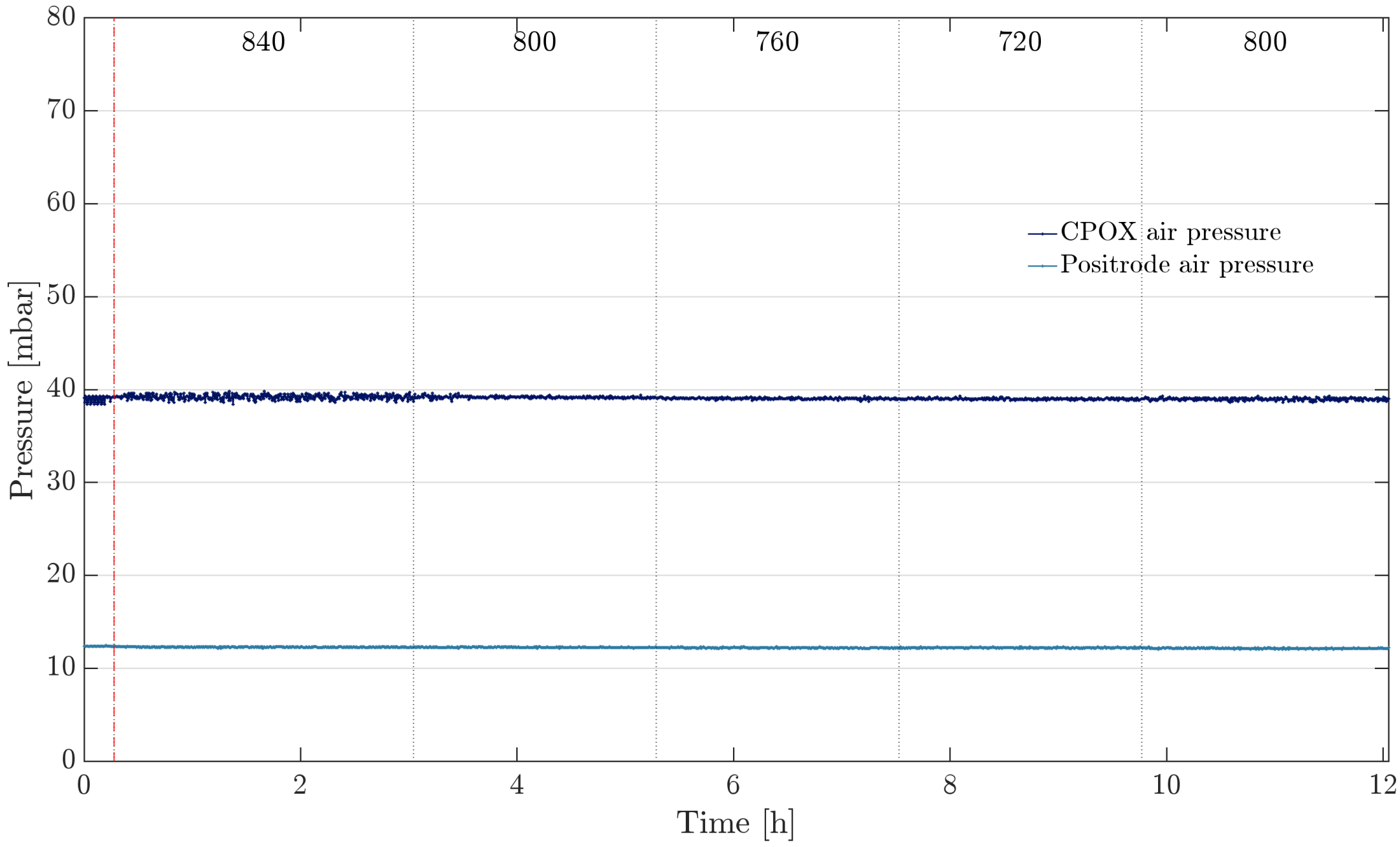

Figure S28. 2-input case for max. CHP efficiency: Outlet pressures of the blowers during the CA RTO test with natural gas blower workload and current manipulated, where the red dash line indicates the activation of CA RTO algorithm and the numbers segmented by dotted lines denote the power target in W.

2.2. 5 inputs case with asynchronous control strategy

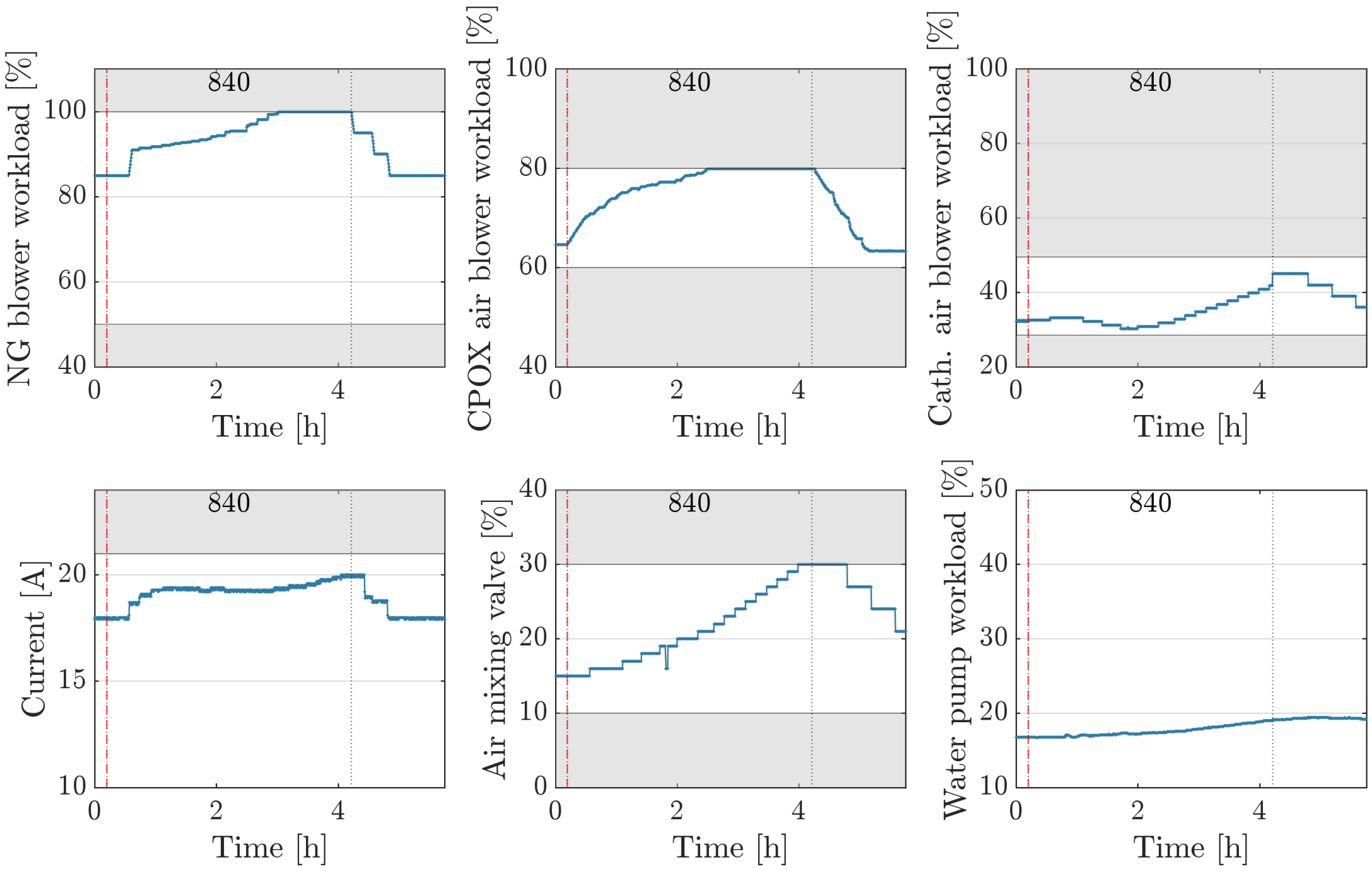

Figure S29. 5-input case for max. CHP efficiency with asynchronous strategy: System inputs during the CA RTO test with natural gas blower workload, CPOX air blower workload, positrode air blower workload, current, and air mixing valve ratio manipulated, where the red dash line indicates the activation of CA RTO algorithm and the numbers segmented by dotted lines denote the power target in W.

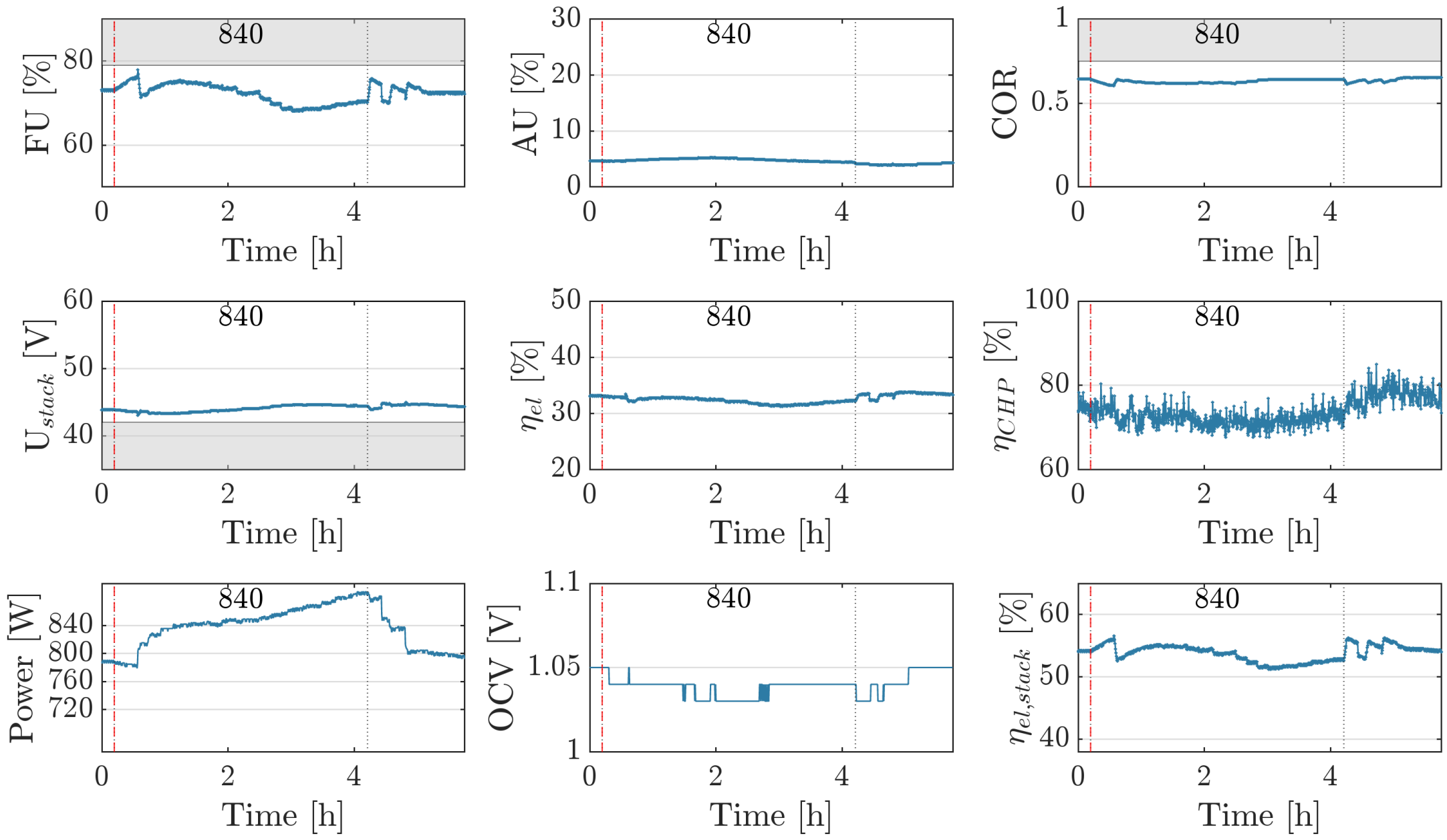

Figure S30. 5-input case for max. CHP efficiency with asynchronous strategy: System key indicators during the CA RTO test with natural gas blower workload, CPOX air blower workload, positrode air blower workload, current, and air mixing valve ratio manipulated, where the red dash line indicates the activation of CA RTO algorithm and the numbers segmented by dotted lines denote the power target in W.

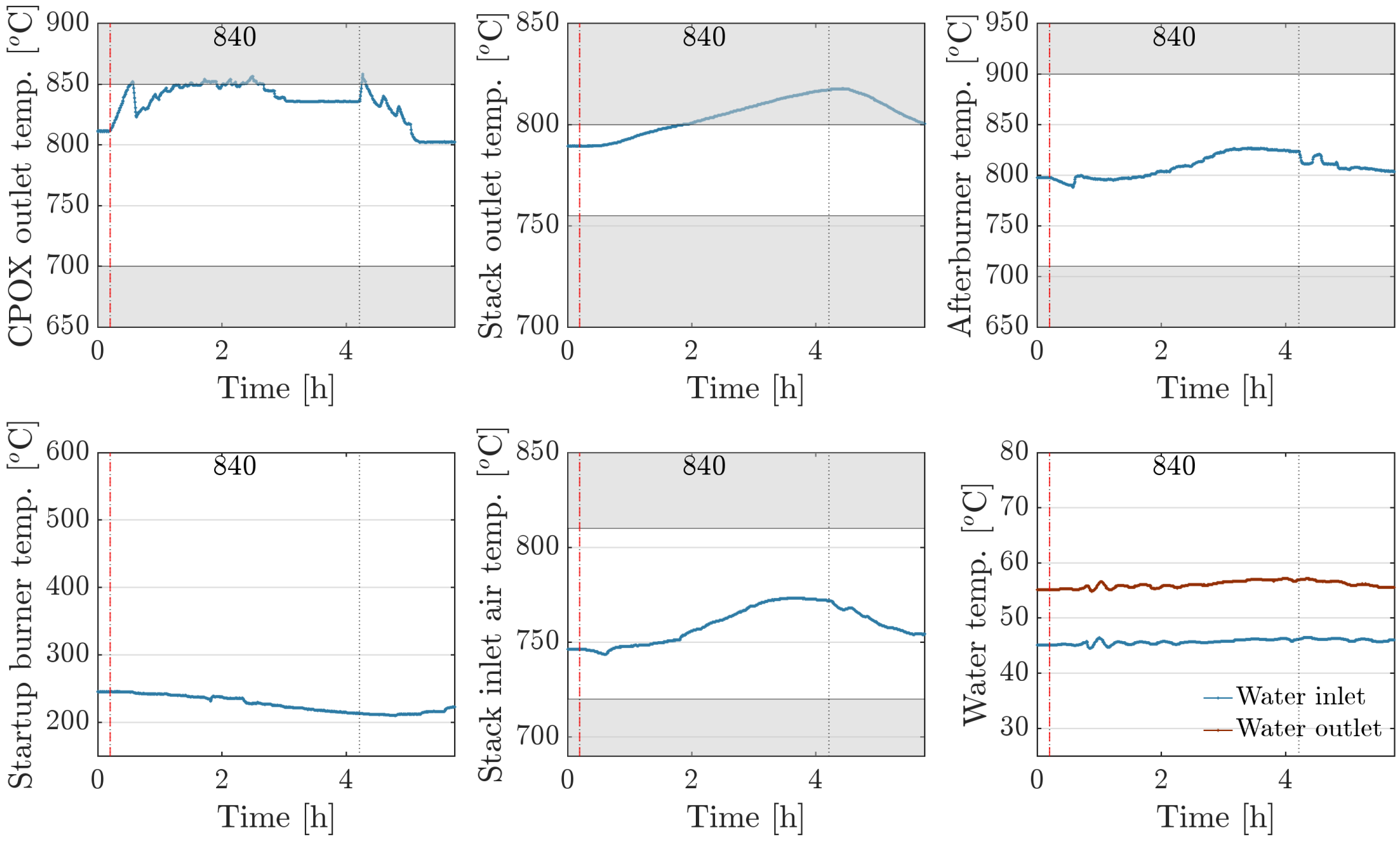

Figure S31. 5-input case for max. CHP efficiency with asynchronous strategy: BoP temperatures during the CA RTO test with natural gas blower workload, CPOX air blower workload, positrode air blower workload, current, and air mixing valve ratio manipulated, where the red dash line indicates the activation of CA RTO algorithm and the numbers segmented by dotted lines denote the power target in W.

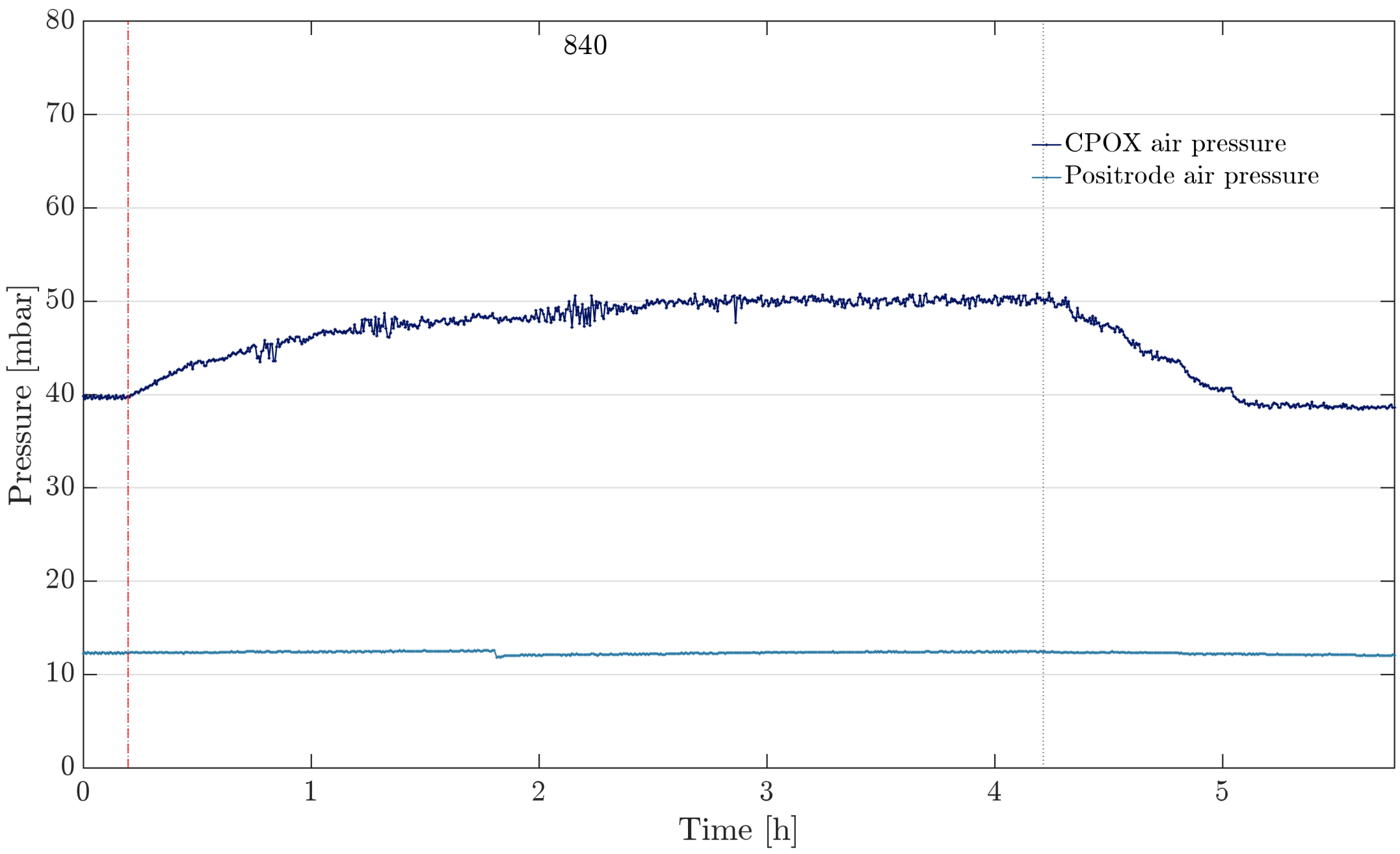

Figure S32. 5-input case for max. CHP efficiency with asynchronous strategy: Outlet pressures of the blowers during the CA RTO test with natural gas blower workload, CPOX air blower workload, positrode air blower workload, current, and air mixing valve ratio manipulated, where the red dash line indicates the activation of CA RTO algorithm and the numbers segmented by dotted lines denote the power target in W.

2.3. 5 inputs case with synchronous control strategy

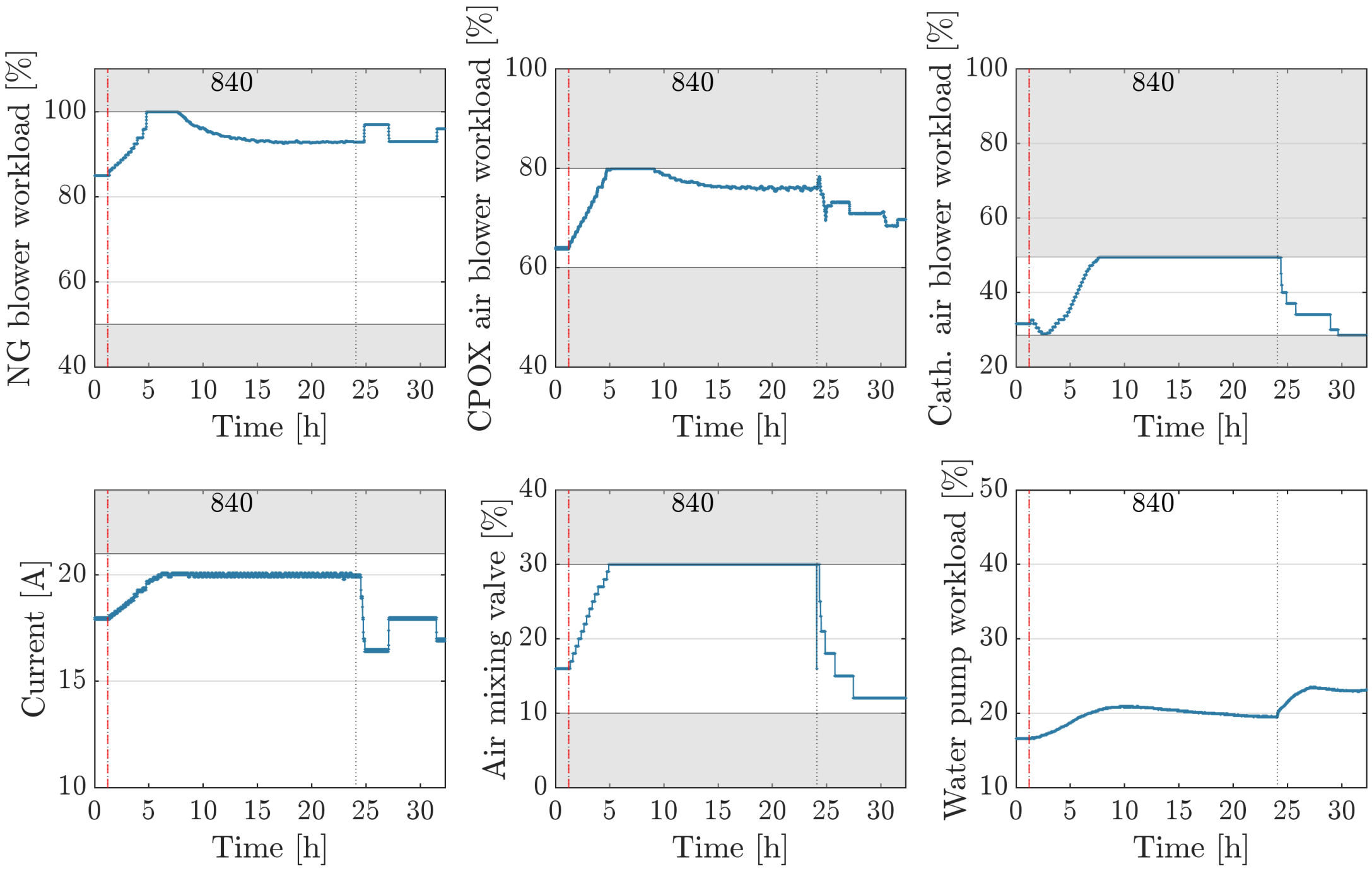

Figure S33. 5-input case for max. CHP efficiency with synchronous strategy: System inputs during the CA RTO test with natural gas blower workload, CPOX air blower workload, positrode air blower workload, current, and air mixing valve ratio manipulated, where the red dash line indicates the activation of CA RTO algorithm and the numbers segmented by dotted lines denote the power target in W.

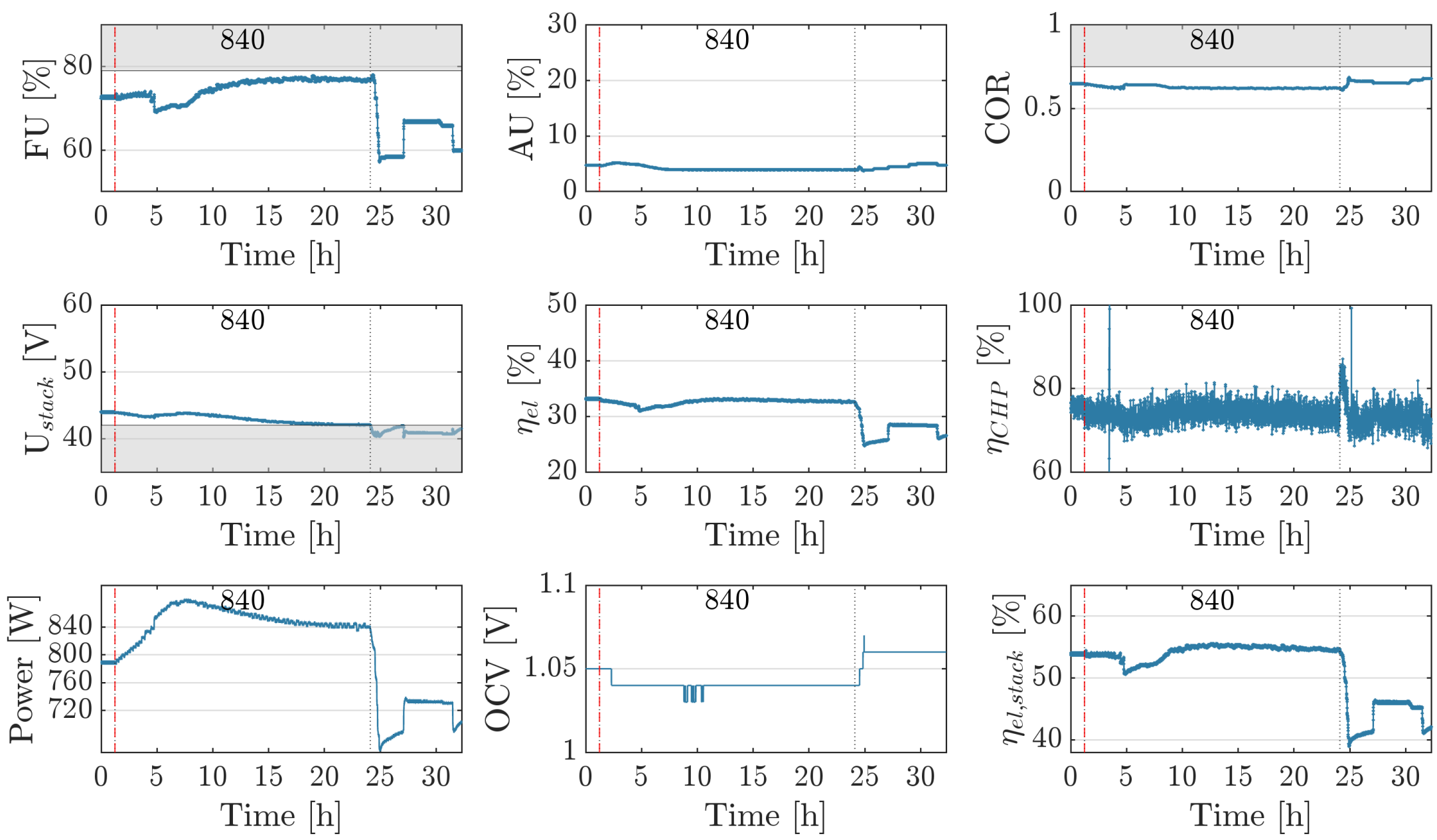

Figure S34. 5-input case for max. CHP efficiency with synchronous strategy: System key indicators during the CA RTO test with natural gas blower workload, CPOX air blower workload, positrode air blower workload, current, and air mixing valve ratio manipulated, where the red dash line indicates the activation of CA RTO algorithm and the numbers segmented by dotted lines denote the power target in W.

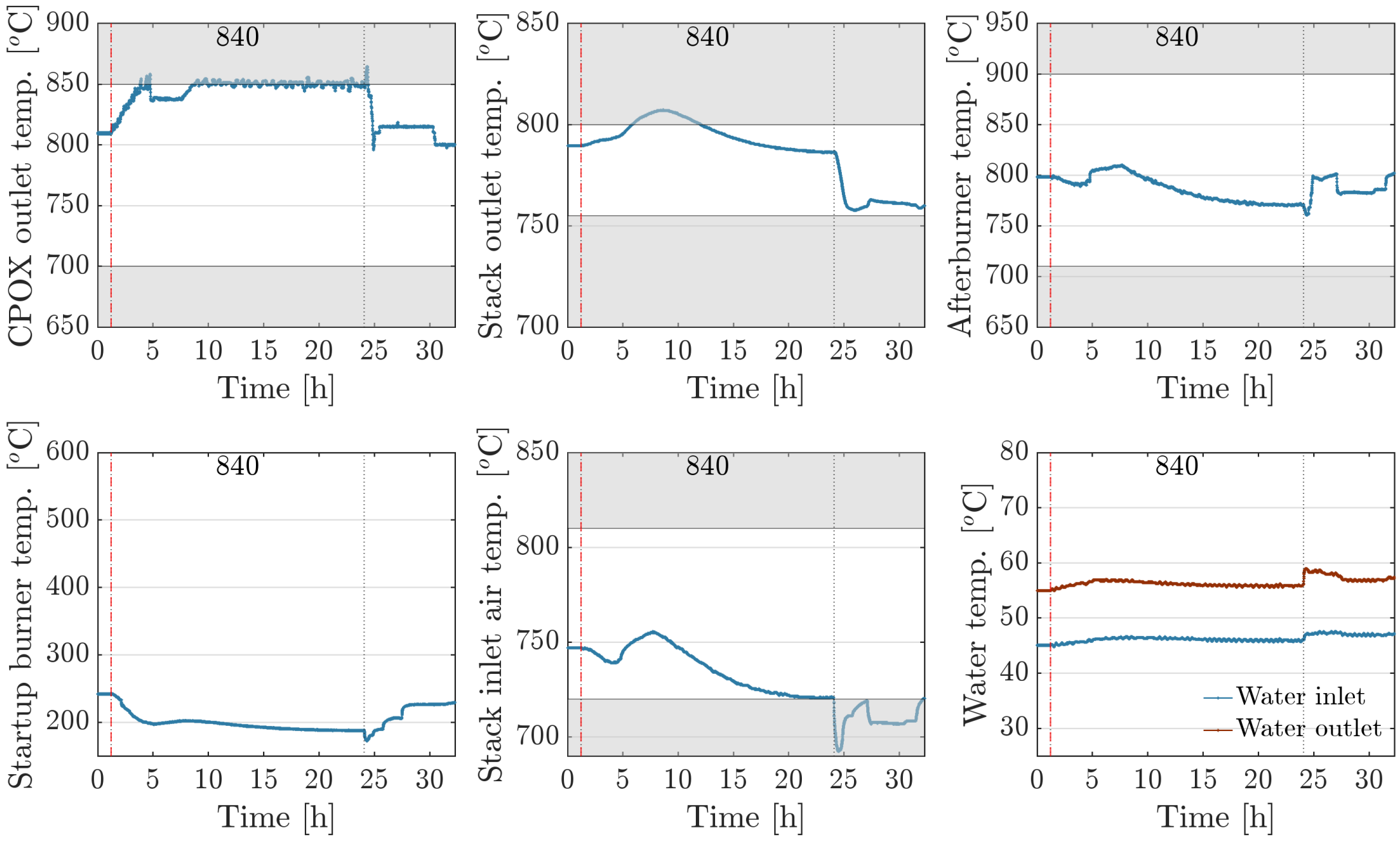

Figure S35. 5-input case for max. CHP efficiency with synchronous strategy: BoP temperatures during the CA RTO test with natural gas blower workload, CPOX air blower workload, positrode air blower workload, current, and air mixing valve ratio manipulated, where the red dash line indicates the activation of CA RTO algorithm and the numbers segmented by dotted lines denote the power target in W.

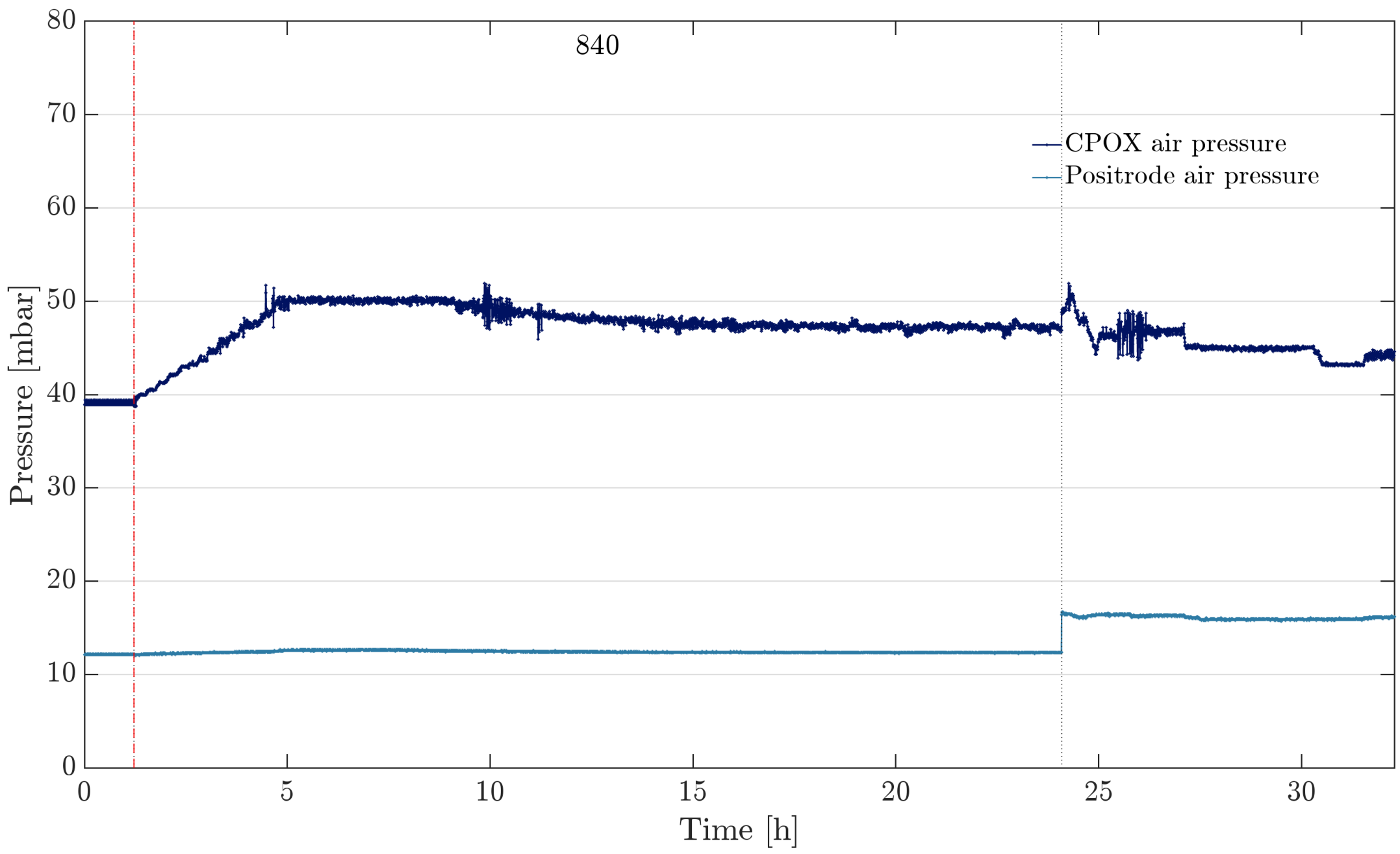

Figure S36. 5-input case for max. CHP efficiency with synchronous strategy: Outlet pressures of the blowers during the CA RTO test with natural gas blower workload, CPOX air blower workload, positrode air blower workload, current, and air mixing valve ratio manipulated, where the red dash line indicates the activation of CA RTO algorithm and the numbers segmented by dotted lines denote the power target in W.
